# Topoisomerase IIβ Activates a Subset of Neuronal Genes that Are Repressed in AT-Rich Genomic Environment

**DOI:** 10.1371/journal.pone.0004103

**Published:** 2008-12-31

**Authors:** Kuniaki Sano, Mary Miyaji-Yamaguchi, Kimiko M. Tsutsui, Ken Tsutsui

**Affiliations:** 1 Department of Neurogenomics, Graduate School of Medicine, Dentistry and Pharmaceutical Sciences, Okayama University, Okayama, Japan; 2 Department of Genome Dynamics, Graduate School of Medicine, Dentistry and Pharmaceutical Sciences, Okayama University, Okayama, Japan; Institute of Genetics and Molecular and Cellular Biology, France

## Abstract

DNA topoisomerase II (topo II) catalyzes a strand passage reaction in that one duplex is passed through a transient brake or gate in another. Completion of late stages of neuronal development depends on the presence of active β isoform (topo IIβ). The enzyme appears to aid the transcriptional induction of a limited number of genes essential for neuronal maturation. However, this selectivity and underlying molecular mechanism remains unknown. Here we show a strong correlation between the genomic location of topo IIβ action sites and the genes it regulates. These genes, termed group A1, are functionally biased towards membrane proteins with ion channel, transporter, or receptor activities. Significant proportions of them encode long transcripts and are juxtaposed to a long AT-rich intergenic region (termed LAIR). We mapped genomic sites directly targeted by topo IIβ using a functional immunoprecipitation strategy. These sites can be classified into two distinct classes with discrete local GC contents. One of the classes, termed c2, appears to involve a strand passage event between distant segments of genomic DNA. The c2 sites are concentrated both in A1 gene boundaries and the adjacent LAIR, suggesting a direct link between the action sites and the transcriptional activation. A higher-order chromatin structure associated with AT richness and gene poorness is likely to serve as a silencer of gene expression, which is abrogated by topo IIβ releasing nearby genes from repression. Positioning of these genes and their control machinery may have developed recently in vertebrate evolution to support higher functions of central nervous system.

## Introduction

In the cell division cycle, gated passage for chromosomal DNA strands is an essential requirement for disentanglement of the DNA link between post-mitotic sister chromatids prior to segregation. The process is catalyzed by the α isoform of topo II that evolved from a single eukaryotic gene through a gene duplication event, which occurred around the time of vertebrate diversification. Topo IIα and its counterpart β (topo IIβ) exhibit similar enzymatic properties on naked DNA *in vitro* [1]. However, as the timing and tissue specificity of expression differs substantially between these isoforms, it is a reasonable assumption that they share separate roles in cellular physiology. Two research groups, including our own, reported the evidence supporting this notion by showing that topo IIβ is required in the late stage of neural differentiation probably through transcriptional induction of neuronal genes [2]–[4]. More recently, other studies demonstrated the regulatory role of topo IIβ in the transcriptional activation of some inducible genes [5], [6]. Use of expression arrays indicated that transcript levels of only a subset of genes are susceptible to depletion of topo IIβ in differentiating neural tissues [3], [7]. Why the enzyme controls only a fraction of genes remained unclear. To answer this question and to elucidate the mechanism of gene regulation by topo IIβ, we hypothesized that relative topographical relationship between topoisomerase action sites and the location of genes controlled by the enzyme should provide an important clue.

A group of topo II inhibitors such as etoposide, often called topoisomerase poisons, have been used to map the topo II action sites on DNA. The drug binds specifically to the enzyme and stabilizes the topo II-DNA covalent complex (cleavable complex) by preventing the ligation of the transiently cleaved DNA strands [8]. Upon addition of strong detergents, the complex is disrupted to liberate DNA fragments with denatured topo II protein covalently attached to their 5′-termini [9]. The drug-induced cleavage sites are then mapped by measuring the distance from end-labeled restriction fragments by Southern blotting. Consensus nucleotide sequences surrounding the cleavage sites have been deduced from *in vitro* experiments with naked DNA and purified enzymes [10]–[12]. As most topo II poisons are permeable into living cells, topo II sites *in vivo* was also mapped with various cell systems [13]. It was suggested that the *in vivo* cleavage sites are highly restricted by the local chromatin structure, being located mostly in the linker segment between phased nucleosomes [14]–[16]. In addition, topo II sites detected *in vivo* do not always coincide with strong naked DNA sites but often correspond to only minor *in vitro* sites. It is clear then that the topo II cleavage consensus *in vitro* is inadequate to predict its *in vivo* sites. It has been generally accepted that topo II cleavage sites are within an AT-rich region often in the vicinity of the matrix attachment region (MAR/SAR) [17]. However, a large-scale or genome-wide mapping of *in vivo* topo II action sites has not been attempted.

The mapping technique used in the present study also utilizes etoposide to enrich the reaction intermediate, but the enzyme-DNA complex was recovered by lysing the cells in milder conditions without strong denaturants [18]. After fragmentation of chromosomal DNA, the topo IIβ -DNA covalent complex was concentrated by immunoprecipitation with specific antibody. Resulting DNA fragments were amplified by ligation-mediated PCR and hybridized to tiling arrays to determine their genomic positions. This protocol is similar to chromatin immunoprecipitation (ChIP) except that the cross-linking is based on the formation of topo II-specific reaction intermediate. Thus, the procedure can be regarded as “functional ChIP”. The use of mild conditions for arresting the intermediate brought about two advantages. First, as topo IIβ was linked to DNA mostly through single strand breakage, the cleavage site resided within the fragment and both ends were available for ligation of amplification primers. Second, the other DNA strand, which is transferred through the cleaved strand, remains associated with the enzyme in the immunoprecipitate. This DNA fragment can be recovered in a separate fraction by a stringent washing when it is not contiguous to the cleaved strand. This topo II site mapping strategy, combined with expression array analysis of topo IIβ-regulated genes, enabled us to identify AT-rich genomic sites that appear to be essential for linking topo IIβ action to its target genes.

Because of complicated terminology used in the study, we provide an explanatory document in Supporting Information to avoid possible confusions (Text S1).

## Results

In the primary culture of newborn cerebellar tissue that we used in this study, only precursor granule neurons can survive after the final division *in vitro* [19]. It is a useful experimental system to study the transcriptional induction of neuronal genes during the post-mitotic differentiation that proceeds spontaneously and almost synchronously. In addition, topo IIβ is the only target of topo II-specific inhibitors since these cells do not express topo IIα [3]. Expression of topo IIβ can be inhibited specifically throughout the culture period if ICRF-193, a topo II-specific inhibitor, was added daily.

### Unique identity of topo IIβ-induced genes

**Figure 1:**
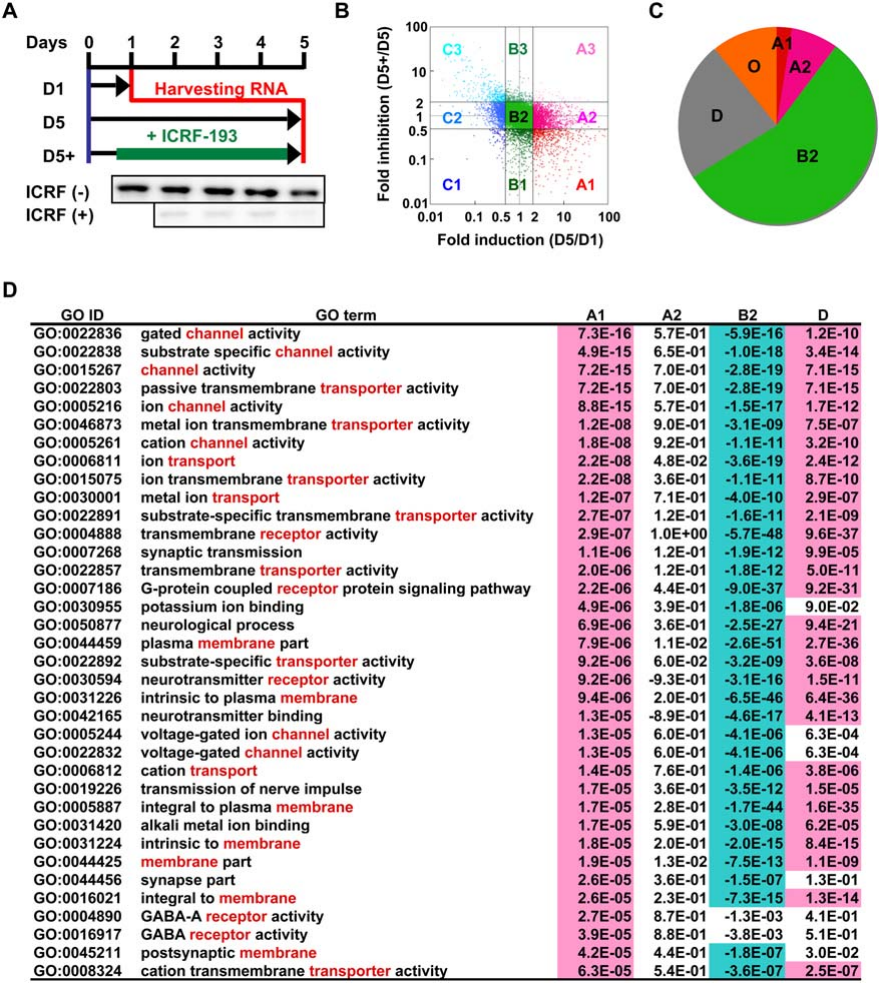
Topo IIβ is required for the induction of selective genes in differentiating cerebellar granule cells. (A) The protocol outline for the analysis of gene expression in rat granule cells in culture. When a specific inhibitor ICRF-193 is added, topo IIβ is degraded rapidly as shown below by immunoblotting. RNA samples were prepared at the time points indicated and subjected to microarray analysis. D1, day 1; D5, day 5; D5+, day 5 in the presence of inhibitor. (B) Grouping of array signals by a scatter graph of induction versus inhibition. Logarithm of signal intensity ratios, (D5)/(D1) vs. (D5+)/(D5), were plotted. Data points separated by 2-fold and 0.5-fold lines are grouped as labeled. (C) A pie graph representation for the rate of genes in each expression group. “D” is unexpressed genes. Other groups of minor population are put together into “O”. (D) Functional characterization of the expression groups by gene ontology (GO) analysis. The result shown here is sorted by *P*-values for A1 group in the decreasing order of statistical significance. Words frequently appear in the term description are shown in red. Overrepresented (*P*<10^−4^) and underrepresented (*−P*<10^−4^) terms are highlighted.

We first categorized the genes expressed in differentiating cerebellar neurons by analyzing the expression array data on the basis of combination of two factors: induction rate during differentiation and susceptibility to ICRF-193. The inhibitor not only inhibits the activity but also depletes the enzyme by selective degradation [20], practically knocking it down at the protein level (Figure 1A). The array data were plotted as a scattered graph of fold-induction versus fold-inhibition and the corresponding genes were classified into 9 groups (Figure 1B). Groups A1, A2, B2, and unexpressed genes (group D) comprised about 90% of analyzed genes (Figure 1C and Figure S5A). The group A1 that makes up only 2.6% is the primary target of the present study since these genes are up-regulated depending on topo IIβ. A2 genes are also up-regulated but are not susceptible to the topo II inhibitor. B2 genes, the largest group, are expressed constitutively and independently of topo IIβ. The array results were confirmed by reverse transcription-quantitative PCR (RT-qPCR) experiments for representative genes from each group (Data S1; Figure S1). Knocking down topo IIβ by RNA interference, instead of ICRF-193, was not suitable for our experimental strategy since some A1 genes got already induced before topo IIβ level was significantly reduced. However, auxiliary use of the inhibitor at the initial step of the culture demonstrated that topo IIβ siRNA is also effective to inhibit the A1 gene induction (Figure S2).

To see whether these expression groups reflect some functional characteristics, the gene ontology (GO) database was used to find over- or under-represented terms in each group (Figure 1D). GO terms related to neuronal function (channel, transporter, receptor activities) were clearly over-represented in A1 genes whereas the same terms were not enriched in A2 and were under-represented in B2 genes. Characteristic GO terms in A2 genes were extracellular matrix and anion transport (Table S5). Thus, A1 and A2 are functionally distinct gene groups although their expression levels both increase during differentiation. As expected, the GO analysis showed that B2 genes are mostly housekeeping genes. Interestingly, unexpressed genes (group D) shared considerable GO terms with A1 genes, suggesting that this gene group might behave like A1 in other neural tissues or in other developmental stages.

### Genomic environment of A1 genes

**Figure 2:**
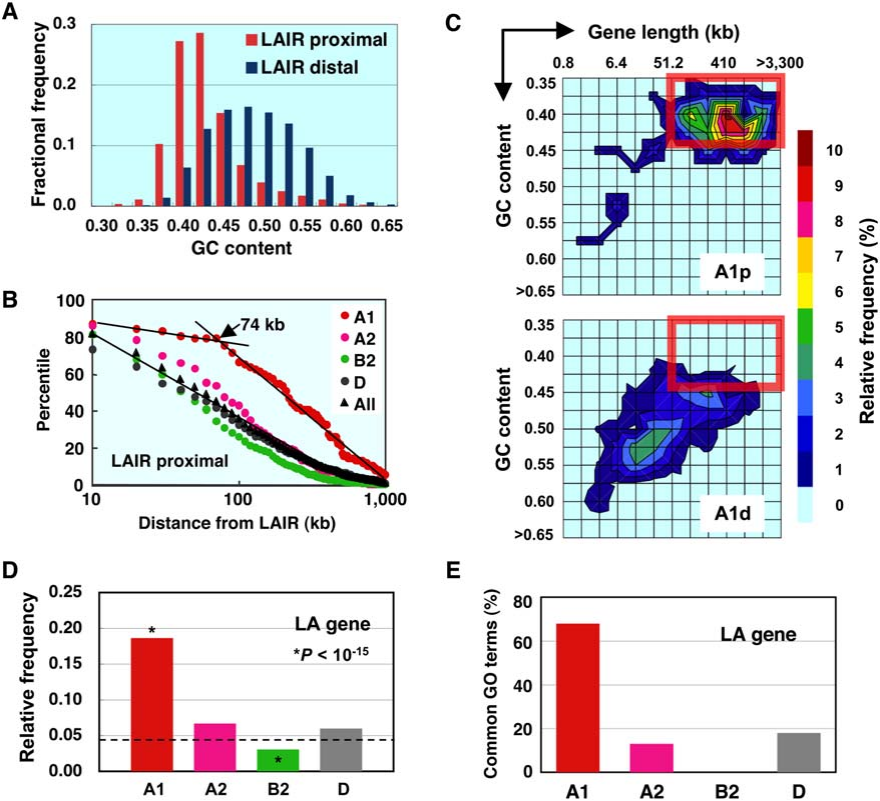
A1 genes occupy a unique position in the genome. (A) Genes juxtaposed to LAIR (LAIR proximal) are more AT-rich than the rest of the genes (LAIR distal). The difference in overall GC contents between the two groups is highly significant (*P*<10^−15^). (B) A considerable proportion of LAIR-proximal A1 genes have a long transcribed region. Percentiles of the LAIR-proximal genes are plotted against the logarithm of distance from the margin of LAIR. (C) Contour maps of frequency distribution for LAIR-proximal (A1p) and LAIR-distal (A1d) A1 genes as a function of logarithmic gene length and GC content. Relative frequency levels are reflected in thermal display. The boxed area represents “long and AT-rich”. (D) Relative frequency of LA genes in various expression groups. The occurrence rate of LA gene in all gene groups (broken line) was used as a reference for chi-squire test. (E) Percentages of GO terms shared between LA gene and various expression groups. Only significant GO terms (*P*<10^−4^) overlapping with those of LA genes were counted.

We next explored how A1 genes are physically different from the other gene groups. When locating A1 genes on genome browser displays, we noticed that they frequently reside close to large gene-poor regions that are mostly AT-rich. For this type of analysis, we first constructed a rat genome compilation (exRefSeq) by combining the data from rat, mouse, and human RefSeq genes (Figure S3). The rat genome was then divided into genic and intergenic regions and they were further classified into four classes each by length and GC content (Figure S4). In the present report, the word “genic” stands for transcribed regions of protein-coding genes. The intergenic region named LAIR (long AT-rich intergenic region) has a large genomic coverage (50% of intergenic and 35% of total genome). About 11% of rat genes were juxtaposed to LAIR (designated LAIR proximal). GC contents of LAIR-proximal genes were clearly biased towards AT-rich side in base composition as compared with genes that are not juxtaposed to LAIR (LAIR distal) (Figure 2A). When compared with other classified gene groups, a higher proportion of A1 genes (24%) were LAIR proximal with statistical significance (Figure S5B). Furthermore, the LAIR-proximal A1 gene contained a distinct population of large genes (>74 kb) as shown in Figure 2B. No such subpopulation was present in other gene groups as well as in LAIR-distal genes (not shown).

Therefore, typical A1 genes can be characterized by three properties: LAIR-proximal, long, and AT-rich. The category of genes with all these characters will be referred to as “LA genes” hereafter. We adopt the average gene length (51 kb) and overall GC content (0.44) to define the thresholds (Figure S4). A two dimensional representation of these variables showed that about 80% of LAIR-proximal but only 10% of LAIR-distal A1 genes are long and AT-rich (Figure 2C). The proportion of LA gene in total A1 genes was significantly higher than that of other gene groups (Figure 2D). The strong link between A1 and LA genes was further demonstrated by the gene ontology analysis (Figure 2E). A higher proportion of A1 genes (68%) shared statistically significant GO terms with LA gene as compared to other gene groups (see also Figure S6).

Based on these results, it should be possible to predict LAIR-proximal A1 genes without knowing their actual response to topo II inhibitors. To test this, from the genes without any assigned array probes (group N), we selected five LA genes that are up-regulated during the cerebellar differentiation and sharing similar GO terms with A1 genes. Expression profiles analyzed by RT-qPCR showed that 4 out of 5 candidate genes indeed behave like A1 genes (Figure S7), corroborating the hypothesis that topo IIβ requirement for transcriptional induction of a gene is closely related to its genomic environment.

### Mapping of topo IIβ action sites

**Figure 3:**
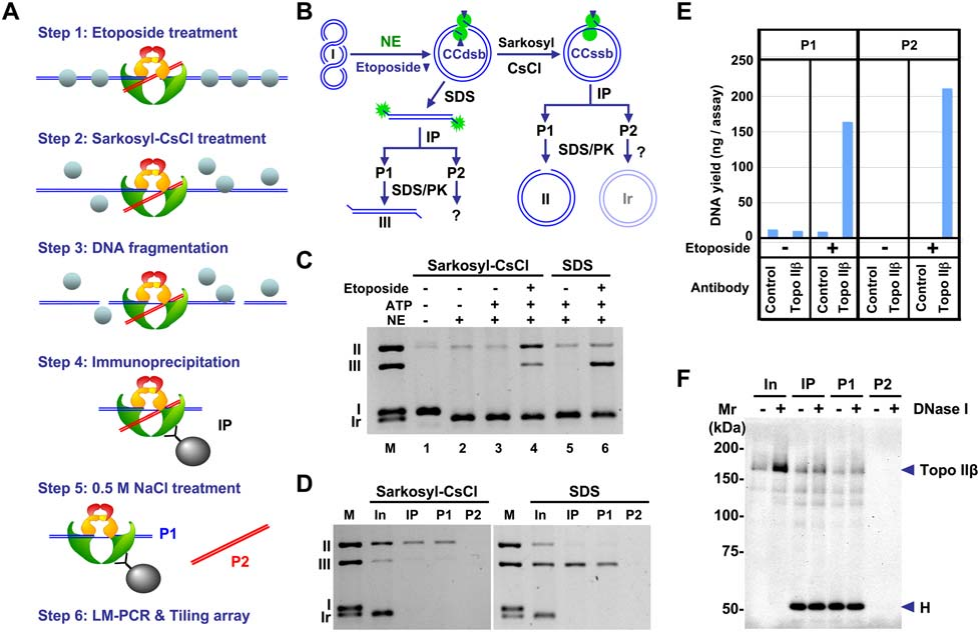
The two segments of DNA interacting with topo IIβ in action can be separated by e-TIP. (A) Schematic representation of the eTIP/tiling array procedure. Type II topoisomerase cleaves a DNA duplex (G-segment, blue) to make a gate for another duplex (T-segment, red) to be transferred through. In the eTIP procedure, both DNA segments are separated together from other DNA fragments in the immunoprecipitation step (Step 4) and separated later into P1 and P2 DNA fractions that are enriched in G-segment and T-segment, respectively (Step 5). Chromatin proteins (depicted as spheres) are largely dissociated from DNA in Step 2. (B) Schematic representation of an eTIP model reaction *in vitro*. Plasmid pUC18 was incubated with rat brain nuclear extract containing topo IIβ, in the presence of etoposide. The reaction was stopped by SDS or Sarkosyl-CsCl and the DNA cross-linked to the enzyme was fractionated by immunoprecipitation under eTIP conditions. NE, nuclear extract; CC_dsb_, cleavable complex with double strand break; CC_ssb_, cleavable complex with single strand break; IP, immunoprecipitation; P1/P2, IP fractions; SDS/PK, Proteinase K treatment in presence of SDS; I, supercoiled DNA; II, nicked circles; Ir, relaxed circles; III, linear DNA. (C) Topo II reactions with or without components indicated were terminated by Sarkosyl-CsCl or SDS. Samples were treated with SDS/PK and separated in 1% agarose gel electrophoresis with 0.5 µg/ml ethidium bromide. (D) After the topo II reaction with etoposide (lane 4 and 6 in Figure 3C), products were fractionated under eTIP conditions and purified DNA was analyzed by agarose gel electrophoresis. M, DNA markers; In, input for immunoprecipitation, IP, immunoprecipitate; P1/P2, IP fractions. (E) The eTIP DNA fractions P1 and P2 were obtained from granule neurons at day 2 under the conditions indicated. The DNA yield shown here is from one plate of culture (∼2.5×10^7^ cells). (F) Detection of topo IIβ in eTIP fractions. Cultured granule neurons treated with etoposide were subjected to eTIP analysis as described. IP samples, with or without DNase I digestion, were directly applied to 6.5% SDS-PAGE and analyzed by immunoblotting with anti-topo IIβ IgG. H, heavy chain of IgG.

As a next step, we intended to locate direct action sites of topo IIβ on DNA, not just binding sites. This can be done by using etoposide, a topoisomerase poison, which stabilizes the reaction intermediate to cross-link the enzyme to DNA at the site of action. The enzyme-DNA complex was recovered by immunoprecipitation with specific antibody. We name this technique eTIP for etoposide-mediated topoisomerase immunoprecipitation (shown schematically in Figure 3A). Treatment of the topo II-DNA complex with strong denaturants like SDS generates a double strand DNA break with denatured enzyme covalently attached to 5′-ends. In the present study, however, we applied milder conditions with a combination of detergent (Sarkosyl) and high salt (CsCl) to preserve the topo II dimer structure attached to a segment of DNA (G-segment) through a single strand break. An important consequence here was that the other segment (T-segment) is also trapped noncovalently and released later by high-salt treatment when it is not contiguous to the cleaved strand (Figure 3A, Step 5).

We confirmed the legitimacy of eTIP procedure by using a model reaction with circular DNA substrate and nuclear extract as enzyme source (Figure 3B). It should be noted that in this system G- and T-segments are contiguous in one strand and no DNA will be released in P2 fraction but complete reversion of the intermediate after immunoprecipitation step would generate closed circles in P2. The nuclear extract contains topoisomerase I activity and supercoiled substrate was completely relaxed in the absence of ATP, whereas nuclease activity in the extract was negligible (Figure 3C, lane 2). When the reaction was stopped by Sarkosyl-CsCl under the conditions used for eTIP, open circular form II DNA was generated depending on etoposide (Figure 3C, lane 4) and immunoprecipitated by topo IIβ antibody (Figure 3D). Thus, form II DNA in P1 can be accounted for by the topo IIβ cleavable complex arrested at single strand breakage (CC_ssb_). No form II or form Ir relaxed DNA was detected in P2 fraction indicating that the enzyme-DNA complex is not dissociated from the beads and reversal of the cleavage does not take place in the stringent washing conditions (Figure 3D). As expected, form III DNA instead of form II DNA was detected in P1 fraction when SDS was used in place of Sarkosyl-CsCl to stop the reaction (Figure 3D).

To evaluate the specificity of eTIP, DNA yields in the eTIP fractions were compared in four conditions (Figure 3E). Cell lysates from granule cells with or without etoposide treatment were immunoprecipitated with either control mouse IgG or anti-topo IIβ monoclonal antibody. The DNA yields in both P1 and P2 fractions were dependent on etoposide treatment and antibody specificity, indicating that DNA in both fractions derived from the arrested intermediate of topo IIβ. To see whether topo IIβ is present in P2 fraction, nuclease-treated fractions were subjected to Western blotting (Figure 3F). Absence of topo IIβ protein in P2 fraction is consistent with the notion that substantial amount of DNA in P2 most likely originates from the T-segment trapped in the topo IIβ CC_ssb_, and not from salt-labile antibody-enzyme interactions.

**Figure 4:**
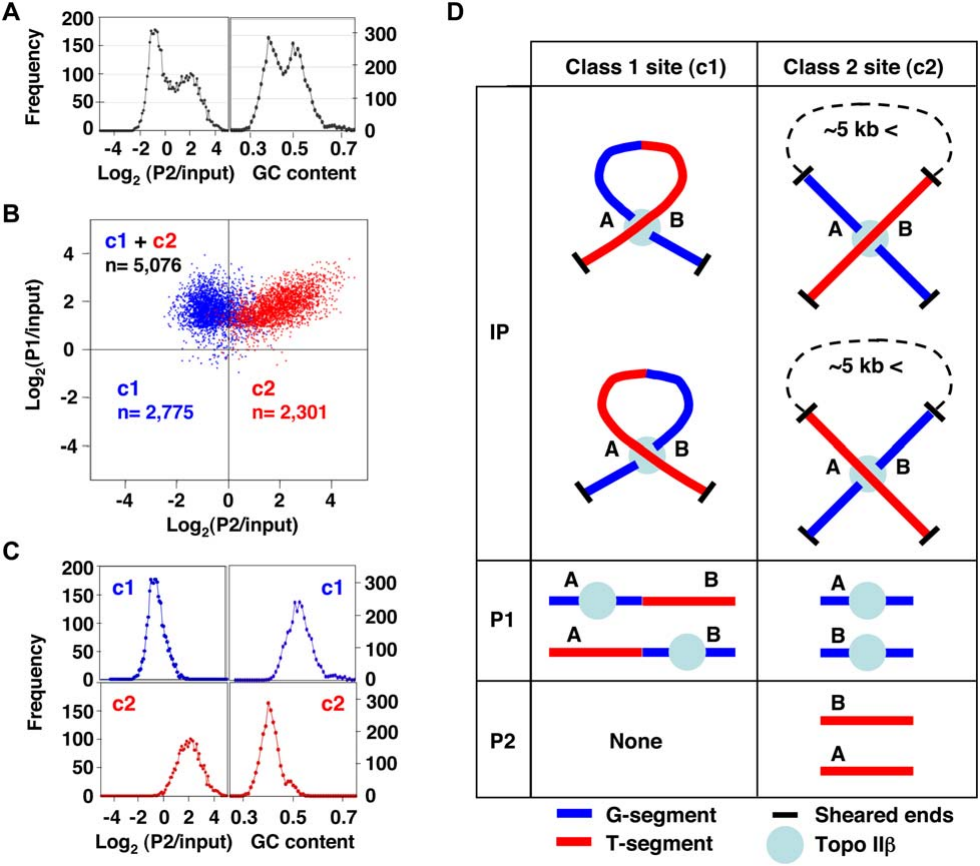
Mapping of topo IIβ action sites reveals two distinctive genomic targets. (A) Analysis of eTIP DNA fractions on tiling arrays showed a binary nature of the composition of P1 DNA as plotted against P2/input or local GC content around toposites (±250 bp from the probe centre). (B) A plot of significant P1 signals scattered along the P1 and P2 axes after clustering with P2/input and local GC content (Ward's method). Two classes of toposites (c1, c2) are distinguished and their numbers (n) are indicated. (C) Frequency distribution of classified toposites against P2/input or local GC content, demonstrating the successful classification of toposites by the clustering approach. (D) Diagram of the relationship among G/T-segments, P1/P2 IP fractions, and c1/c2 toposites. Unique genomic sites involved in the reaction with topo IIβ are labeled A and B.

For genome-wide mapping of topo IIβ action sites (termed toposites), the eTIP DNA fractions were prepared at the second day of granule cell differentiation. We chose this timing because mRNA expression is maximally induced by day 3 in many A1 genes (see Figure S1), indicating that the involvement of topo IIβ in gene induction starts before day 3. In addition, our previous study showed that topo IIβ is required only in the early period of culture [3]. DNA fragments purified from P1 and P2 fractions were hybridized to tiling arrays. In designing the array, we selected 7 genomic regions (about 79 Mb in total), each containing at least one A1 gene. A correlational analysis with statistically significant P1 signals from the array data suggested that P1 DNA is composed of two components (Figure 4A). Multi-factor clustering of P1 signals against P2 signals and local GC content clearly separated the toposites into 2 classes (Figure 4B). The scatter graph indicates that one of the classes, labeled c1, was detected only in P1 fraction whereas c2 was present in both P1 and P2 fractions. The frequency distribution also showed that the two factors used for the clustering are well discriminated in the resulting classes: the c1 site depleted from P2 is GC-rich and the c2 site enriched in both P1 and P2 is AT-rich (Figure 4C). These results were consistent with the preliminary observation obtained by shotgun cloning of eTIP DNA fractions (Figure S8).

To avoid possible confusions, the relationship among the terms used in the study is represented schematically in Figure 4D. Unique positions on the genome are labeled A or B, which can be either G-segment or T-segment (both cases are depicted). The c1 toposite shown on the left column does not generate free DNA in P2 fraction. This situation corresponds to the model experiment with plasmid DNA (Figure 3B). If the distance between A and B are larger than ∼5 kb, these sites are likely to reside on separate fragments since the average fragment size is less than 5 kb. In this case both genomic regions (A and B) are detected in both DNA fractions (P1 and P2) and the action site is classified as c2.

Positions of c1 and c2 toposites in all the analyzed chromosomal regions are illustrated in Figure S9. As expected, the pattern of global toposite distribution appeared to reflect the local GC content. Although less frequently, toposites were detected also in unexpressed genes (group D) and in genes whose expression is independent of topo IIβ. It is worth noting that *Ndst4*, an A1 gene predicted from the positional information (Figure S7), is located in the tiled region on chromosome 2. *Ndst4* and the adjacent LAIR are both enriched with c2 toposites.

### Topographical link between toposites and A1 genes

**Figure 5:**
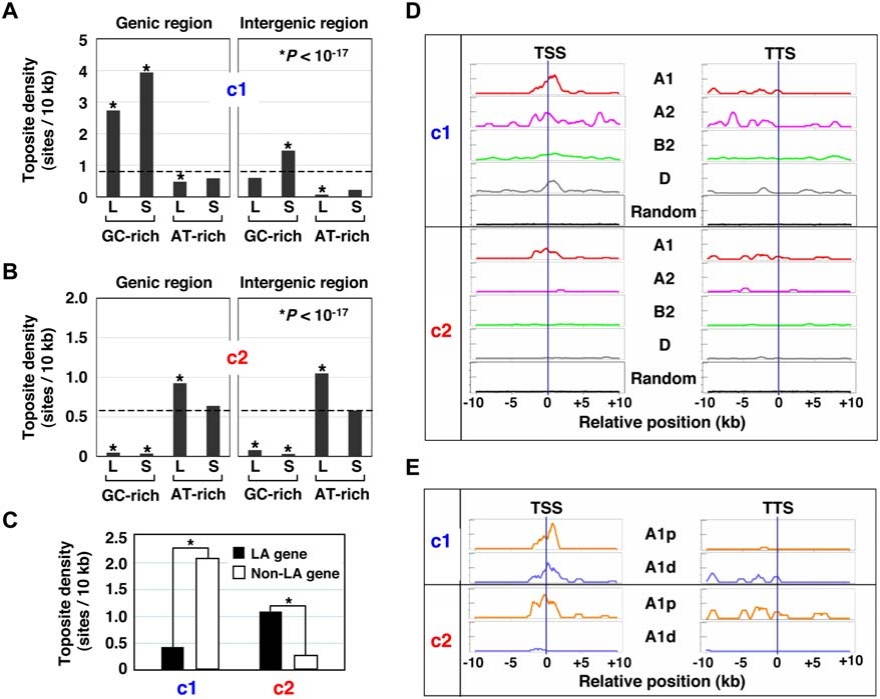
Patterns of toposite distribution corroborate the involvement of topo IIβ in the transcriptional regulation of A1 genes. Regional frequency of toposites expressed by number of sites per 10 kb (site density). Subgenomic regions are classified into 8 classes by their length and GC content (Figure S4). L, long; S, short. (A) Density distribution of c1 toposite. (B) Density distribution of c2 toposite. (C) Reciprocal distribution of the two classes of toposites in LA gene and the rest of the genes (non-LA gene). The difference between these gene groups is highly significant (*P<10^−79^). (D) Clustering of toposites around the border of transcription units. Toposite densities expressed as scan units are shown on the vertical axis (full scale = 2.0). The randomly generated toposite controls for each gene group are plotted together (overlaid) on the bottom. (E) LAIR-proximal A1 genes are characterized by the presence of c2 toposites around the boundaries of transcription units. A1p, LAIR-proximal A1; A1d, LAIR-distal A1.

It is reasonable to assume a certain topographical relationship between genomic locations of toposites and the regulated genes, namely A1 genes. As a whole, less than one toposite was present in every 10 kb for both c1 and c2 sites (Figure 5A, B). These sites, however, were concentrated or depleted in particular genomic regions depending on their regional GC contents. As defined in Figure S4, genomic regions were classified into 8 classes. c1 sites are clearly enriched in GC-rich genic regions (Figure 5A). The c1 site enrichment in short GC-rich intergenic regions may reflect promoters within gene-dense genomic areas (Figure S9). For both genic and intergenic regions, c2 sites are largely depleted from GC-rich regions and concentrated in long AT-rich regions (Figure 5B). The tiled region contained 318 genes, of which 140 (44%) harbored more than one toposite per gene and 32 (10%) were LA genes. A large proportion of LA gene (81%) contained toposites as compared to non-LA genes (40%). When classified toposite densities in the genic region were compared, c1 sites were significantly enriched in non-LA genes, whereas c2 sites were concentrated in LA genes (Figure 5C).

To see whether toposites are clustered in a particular region with respect to genes, we finally analyzed the site distribution at the boundary of transcription units that were aligned either at the transcription start site (TSS) or at the termination site (TTS) (Figure 5D). A discrete peak of c1 site was detected around TSS in A1 and D groups, while A2 gene exhibited multiple c1 peaks around TSS and inside the gene. Distribution of c1 sites in the 3′ region of A1 and A2 genes was more irregular but appeared to be confined within the transcription unit. In contrast to c1 sites, c2 sites were basically restricted to A1 genes. When A1 genes were sorted into two groups, LAIR proximal (A1p) and LAIR distal (A1d), it is now clear that c2 sites are located exclusively around A1p genes (Figure 5E). All the LAIR-proximal A1 genes found in the tiled region were LA genes whose promoters were present inside the adjacent LAIR (Table 1). Thus, c2 sites are likely to be essential for the regulated expression of LA genes. Meanwhile, LAIR-distal A1 genes appear to be regulated by c1 sites.

**Table 1:** List of A1 genes located in the region analyzed by tiling array.

	Gene name	Chr	Length	GC	Long	AT-rich	LA gene	5′-LAIR
**LAIRp**	PKIA	2	75,379	0.377	1	1	1	1
	NDST3	2	168,066	0.416	1	1	1	1
	NDST4	2	337,446	0.381	1	1	1	1
	CAMK2D	2	269,505	0.414	1	1	1	1
	KCND2	4	507,449	0.382	1	1	1	1
	CNTN4	4	856,312	0.392	1	1	1	1
	PDE4B	5	468,195	0.407	1	1	1	1
	GABRB2	10	224,006	0.383	1	1	1	1
	SGCD	10	404,807	0.407	1	1	1	1
	CDH11	19	156,643	0.389	1	1	1	0
**LAIRd**	ANK2	2	321,155	0.424	1	0	0	0
	TSPAN33	4	23,520	0.478	0	0	0	0
	D330017J20RIK	4	52,406	0.449	1	0	0	0
	ITPR1	4	324,832	0.443	1	0	0	0
	CPNE9	4	23,676	0.474	0	0	0	0
	RAVER2	5	79,562	0.448	0	0	0	0
	SLC35D1	5	46,149	0.440	0	0	0	0
	DAB1	5	150,599	0.443	1	0	0	0
	ADAM19	10	92,490	0.469	0	0	0	0
	RRAD	19	3,241	0.536	0	0	0	0
	PDP2	19	5,688	0.472	0	0	0	0
	CAR7	19	9,393	0.509	0	0	0	0
	MT3	19	1,849	0.534	0	0	0	0

Binary discriminants designate: Long, gene length longer [1] or shorter [0] than 51 kb; AT-rich, GC content lower [1] or higher [0] than 0.44; LA gene, LA gene [1] or non-LA gene [0]; 5′-LAIR, LAIR is present [1] or absent [0] upstream.

## Discussion

Type II DNA topoisomerases catalyze the strand passage events between the two segments of DNA, termed G- and T-segments [21]. The G-segment contains the transient gap generated by the enzyme for the passage of T-segment. Under the conditions used in the eTIP procedure, the enzyme-DNA intermediate is converted mainly to a form with a single-strand breakage at the site of action, suggesting that the cleavage sites on G-segments are concentrated in the P1 DNA fraction (Figure 3A). The DNA yield experiments demonstrate the specificity of recovered DNA fragments in terms of etoposide treatment and binding to topo IIβ. The absence of DNA in the P2 fraction without etoposide treatment is a strong evidence for the assumption that P2 DNA is directly associated with the reaction intermediate of topo IIβ, not just with the enzyme. It also indicates that little nonspecific DNA is contaminating the P2 DNA fraction, implying that the stringency of washing conditions in Step 4 is sufficient. Thus, the DNA in P2 fraction is closely involved in the topoisomerase reaction and most likely representing the T-segments that are trapped between the subunits of the enzyme through noncovalent interactions. The successful retention (Step 2) and release (Step 5) of T-segments in the eTIP procedure may be accounted for by the difference in the chaotropic potential of the cations used in these steps: Cs^+^ and Na^+^, respectively.

**Figure 6:**
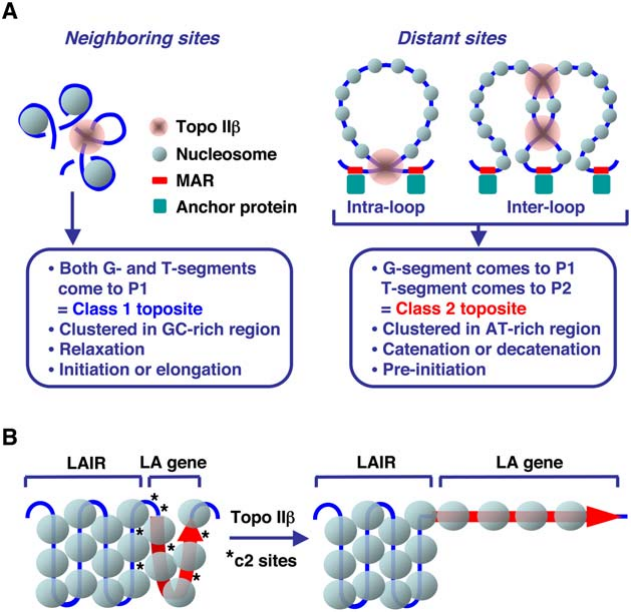
A model that explains the topographical relationship of *in vivo* toposites and target genes. (A) Possible link between the reaction mechanism of type II DNA topoisomerases and the generation of two classes of toposites. MAR stands for matrix attachment region. Nuclear matrix proteins interacting with MAR are designated by “anchor protein”. (B) Activation of LA genes by topo IIβ. Red arrow and asterisks designate LA gene and c2 toposites, respectively.

The characteristic partitioning of DNA fragments into eTIP fractions (P1 and P2) provided an important clue to understanding the mechanism of topo IIβ action *in vivo*. A working hypothesis implied by the present study is summarized in Figure 6A. We suppose that 1) the enzyme can recruit any part of the genome as G- or T-segment. 2) The enzyme has little preference as to which segment should be G or T. 3) If G- and T-segments are on different DNA fragments, the eTIP procedure sorts these DNA segments into P1 and P2 fractions, respectively. When G- and T-segments are closely positioned on the genome, for instance less than 5 kb, both segments will be recovered in P1 and the site is classified as class 1 (c1). In this case, the most probable topological change caused by the reaction is the relaxation of superhelical turns that are built up locally [22]. This reaction may facilitate the initiation or elongation steps of transcription. In contrast, when these DNA segments are located distantly, they are inevitably separated into P1 and P2 fractions interchangeably and the site is classified as class 2 (c2). An essential prerequisite for this type of reaction is the formation of a DNA crossing, which is preferentially recognized by topo II [23]. Since the crossed DNA segments can either be G- or T-segment by chance, they are fractionated into both IP fractions, P1 and P2. These crossings should occur more frequently at the base of chromatin loops that are maintained by the interaction between MAR and the nuclear matrix. This is consistent with the fact that MAR is usually found in AT-rich region like c2 toposites. The strand passage at inter-loop c2 sites causes catenation or decatenation between the loops that are equivalent to topologically independent DNA circles. These topological changes may induce the decondensation of local chromatin structure [24], leading to the formation of active initiation complex for productive transcription.

Transcriptional profiling of topo IIβ knockout mouse embryos showed that the enzyme regulates only a small fraction of genes (1–4%) in the late embryonic brain cells [7]. When the down-regulated genes in the knockout mice were compared to our results, a significant proportion of them were A1 genes or LA genes, being consistent with the idea that similar subsets of genes with similar genomic locations are controlled by topo IIβ in the late stages of neuronal development (Figure S10). Involvement of topo IIβ has also been reported in nuclear receptor-mediated regulation of inducible genes [5], [6]. In these cases, however, the enzyme mostly targets the promoter region of non-LA genes. We further noticed a striking parallelism between our findings and the widespread monoallelic expression of human autosomal genes [25]. Large fraction of monoallelically expressed genes encodes cell surface and membrane proteins. All the GO terms over-represented in these genes with high statistical confidence (Table S3 in the report) coincided with those terms for LA gene (Table S5). Re-analysis of their data indeed showed a high incidence of LA genes in the monoallelic expression group (Figure S11). Similarly, a large proportion of autism-related genes identified in the recent report [26] were LA genes (data not shown). Since LA genes contain many neuronal genes (Table S5), their monoallelic expression would predict a cellular mosaicism and may explain a phenotypic polymorphism of psychotic conditions or even personality variations in normal individuals. Apparent similarity between LAIR and gene deserts [27] or lamina-associated domains (LADs) [28] is also worth noting.

The c2 toposite identified in this study is unique in that it involves two segments of DNA from distant genomic locations (Figure 6A). According to the model, it is possible that the TSS region of LA gene is in contact with internal sites of the adjacent LAIR (Figure 6B). This type of interaction may repress the transcription of LA genes by condensing the chromatin structure of promoter region. This is consistent with the fact that promoters for 9 out of 10 LAIR-proximal A1 genes in the tiled region are on the LAIR side (Table 1). The same situation is noted in the promoters of LAD-associated genes that are positioned adjacent to LADs [28]. A long-distance physical contact between inactive genes and the gene–poor or inactive genomic regions has also been demonstrated for the inactive β-globin gene in mouse embryonic brain, but not the one actively transcribed in the liver, by a chromosome conformation capture technique (4C) [29]. We propose that LA genes are a distinct category of genes and topo IIβ plays a novel regulatory role by liberating them from the transcriptional repression exerted by juxtaposed LAIR. While the activation mechanism of lamina-associated genes is totally unknown, topo IIβ would be the first candidate for such factors that are actively involved in the gene activation process. In the present study we focused on c2 sites and LA genes but roles of c1 sites in the control of non-LA genes are less clear at present and should be addressed in future studies.

## Materials and Methods

### Animal handling

All experiments were carried out under the control of the Animal Research Control Committee in accordance with the Regulation for Animal Experiments of Okayama University Medical School. All efforts were made to minimize the number of animals used and their suffering.

### Primary culture

Cerebellar tissue was isolated from the Wistar rat 8 days after birth. Minced tissue was treated briefly with trypsin-EDTA and DNase I in PBS. Dispersed cells were plated at 2.5×10^7^ cells per 100 mm plastic dish pre-coated with poly-L-lysine. The cells were incubated at 37°C in DMEM containing 10% FCS, 25 mM KCl, and 60 µg/ml kanamycin sulfate. After 15–16 h, medium was replaced with fresh medium supplemented with 10 µM cytosine arabinoside to suppress the glial growth. The medium change was repeated at 24 h and at every 48 h afterwards. When it is required, expression of topo IIβ was inhibited specifically throughout the culture period by daily addition of 10 µM ICRF-193.

### Immunoblotting

Cells grown on culture dishes were lysed directly in SDS-PAGE sample buffer (50 mM Tris-HCl: pH 6.8, 2 mM EDTA, 2% SDS, 1% 2-mercaptoethanol, 8% glycerol, 0.025% bromophenol blue) and boiled for 5 min. To normalize the loading amounts, concentration of nucleic acids in sample was determined by a fluorometric procedure with Quant-iT RiboGreen RNA Assay Kit (Molecular Probes, Invitrogen). GENios multi-detection microplate reader (Tecan) was used for detection. Samples equivalent to 0.5–2.0 µg of nucleic acids were subjected to SDS-PAGE and then transferred to a PVDF membrane by electroblotting. The membrane was incubated with blocking buffer (5% skim milk, 0.1% Tween 20, 20 mM Tris-HCl: pH 7.6, 150 mM NaCl) for at least 2 h at room temperature. Immunodetection with first antibodies and horseradish peroxidase-conjugated second antibodies (listed in Table S4) was performed according to standard procedure. The peroxidase activity was detected by a chemiluminescence method using an ECL kit (GE Healthcare) and recorded on VersaDoc MP 5000 Imaging Systems (Bio-Rad).

### siRNA experiment

To knockdown topo IIβ in cultured granule cells, transfection of rat topo IIβ siRNA was carried out using an electroporation equipment, Nucleofector, and Rat Neuron Nucleofector Kit (Amaxa). The cDNA sequence of rat topo IIβ (GenBank Accession AB262979) was used to design siRNAs as siGenome SMARTpool siRNA (Dharmacon, GE Healthcare). The control siRNA was Negative Control siRNA (Qiagen). Freshly isolated cerebellar cells were resuspended in 100 µl of Nucleofector solution and mixed with 3 µg of siRNA (7.2×10^6^ cells per reaction). The cell suspension was transferred to the Nucleofector cuvette and the transfection sequence was started using program G-13. Immediately after transfection, 0.5 ml of pre-warmed culture medium was added to the cuvette. Cells were then transferred to a 6-well plate containing 1.5 ml of culture medium and incubated in a humidified incubator (37°C, 5% CO_2_). Three hours later, the medium was replaced with fresh medium. During the time before the RNAi effect becomes significant, topo IIβ was suppressed by 10 µM ICRF-193 between 16 h and 2.5 days, and then the medium was replaced with fresh medium without inhibitor. Our previous study suggested that the effect of ICRF-193 is fully reversible and after removing the inhibitor the normal gene induction sequence proceeds with delays [3]. Total RNA was isolated at the 5th day to assess the expression of selected genes by RT-qPCR.

### Reverse transcription-quantitative PCR (RT-qPCR)

Total RNA was isolated from the granule cells using the RNeasy Mini Kit (Qiagen) according to supplier's instructions. Three micrograms of total RNA was reverse-transcribed in 60-µl reactions as recommended by manufacturer, using 5 µM random hexamer primer, 0.5 mM deoxyribonucleoside triphosphates (dNTPs), 1 unit/µl RNase inhibitors (Takara Bio) and 10 units/µl M-MLV reverse transcriptase, RNase H minus (Takara Bio). Real time qPCR was performed on the GeneAmp 5700 Sequence Detection System (Applied Biosystems) using SYBR Green I chemistry in triplicate reactions. Primer sets for qPCR were based on the rat gene sequences retrieved from the UCSC genome browser site (listed in Table S3). The amplification was carried out in a reaction mixture (30 µl) containing 1× SYBR Green PCR Master Mix (Applied Biosystems), 0.5 µM primers and cDNA equivalent to 15 ng of RNA. Thermal conditions (for all targets) were 50°C for 2 min, 95°C for 9 min, followed by 40 cycles of 95°C for 20 sec and 60°C for 1 min. Specific amplification of correct targets was confirmed by dissociation curve analysis. To calibrate the reaction, the cDNA template was serially diluted and amplified each time along with every gene targets.

### Construction of exRefSeq

Using the rat genome database (rn3, Jun. 2003), refSeq (refSeqAli, refGene, refFlat) and xenoRefSeq (xenoRefSeqAli, xenoRefGene, xenoRefFlat) coordinates were downloaded from UCSC genome bioinformatics (http://hgdownload.cse.ucsc.edu). The annotation date for these files was 29-Jul-2007. Available programs (MySQL, R, and Microsoft Excel) were used to process the data. All the data sets for refSeq and xenoRefSeq were first deposited in a MySQL database and then rat, mouse, and human data were extracted. Items with uncertain genomic position (designated Un or random) were excluded. Non-rat olfactory and vomeronasal receptor genes were also eliminated. Furthermore, the following items were filtered out: RefSeq of low transcript-genome matching (<60%), mouse RefSeq of low homology (<85%), and human RefSeq of low homology (<80%). All the gene names in the remaining items were converted to the mouse format using the programs g:Convert and g:Orth at the g:Profiler website (http://biit.cs.ut.ee/gprofiler/) [30]. Overlapping transcription units with the same gene name, genomic position, and the direction of transcription were unified into a longest hypothetical transcript and the resulting compilation of 17,799 rat genes was named “exRefSeq” that designates “extended RefSeq”. The genic region was defined as a longest stretch of overlapping transcripts (including reverse directions) in exRefSeq. The procedure is illustrated schematically in Figure S3.

### Expression array

Expression microarray experiments were performed mostly at Bio Matrix Research, Inc. (Tokyo, Japan). The labeling procedure was carried out as described by the manufacturer (Affymetrix, http://www.affymetrix.com). One microgram of total RNAs from culture day 1 (D1), day 5 (D5), and day 5 with inhibitor (D5+) were reverse-transcribed to cDNAs by GeneChip One-Cycle cDNA Synthesis Kit. The cDNAs were transcribed to cRNAs *in vitro* with biotinylated nucleotides using GeneChip IVT Labeling Kit. The Test3 array was used for quality control. Biotinylated cRNAs were hybridized to GeneChip Rat Genome 230 2.0 for 16 h at 45°C in GeneChip Hybridization oven 640. After hybridization, arrays were washed and stained with Streptavidin-Phycoerythrin in GeneChip Fluidics Station 450, and scanned on GeneChip Scanner 3000. GeneChip Operating Software (GCOS; Affymetrix) was used for image processing and data acquisition. The expression array data were deposited in GEO (GSE13112).

GCOS was also used to normalize the signal levels between different arrays by applying 50th percentiles and to calculate signal intensities or reliabilities of each probe. Probes with absent call on both D1 and D5 arrays or absent on both D5 and D5+ arrays were relegated to unexpressed genes (group D) and excluded from further classification. Expressed probes were classified into 9 groups (designated A1, A2, A3, B1, B2, B3, C1, C2, C3) by applying 2-fold and 0.5-fold thresholds to the combination of two parameters, induction (D5/D1) and inhibition (D5+/D5) (illustrated in Figure 1B). Both unexpressed and expressed probes were converted to corresponding exRefSeq by g:Profiler. The exRefSeq genes without assigned probes were grouped as “N” (5,090 genes). The following rules were applied for grouping the exRefSeq genes with multiple probes that represent conflicting group assignments. If an A1 group probe is present in the gene in question, it is grouped as “A1” regardless of other probes. If A1 probe is absent but A2 probe is present, that gene is grouped as “A2”. Genes without A1 and A2 probes but having other combination of different probes are grouped as “Mix”. Group D probes in a gene with an unambiguously assignable probe are neglected. In some cases minor groups (A3, B1, B3, C1, C2, C3, Mix) are combined and referred to as “Others”. The gene group classification is summarized in Figure S5A.

### GO analysis

To analyze the gene ontology (GO) annotation of selected rat genes, gene names of exRefSeq were converted to corresponding human gene names using the programs g:Convert and g:Orth at the g:Profiler site. GO statistics were examined by the program GOstat2 to identify the GO terms that are enriched or depleted between two sets of genes (http://gostat.wehi.edu.au/cgi-bin/goStat2.pl) [31]. In the present study, a set of individual gene group was compared to its complementary set. The length of GO path parameter was set to 1. Statistical significance for over- or under-represented GO terms in a group of genes was corrected by the method of Benjamini and Hochberg. As a featured group of genes, neuronal genes were selected by using the NCBI Gene database (http://www.ncbi.nlm.nih.gov/sites/entrez?dbGene) with search terms: neuron* OR neural* OR neurite OR axon AND human[ORGN]. Out of 2,844 human neuronal genes, 1,429 genes that possess rat exRefSeq counterparts were used for GO analysis.

### eTIP procedure

Primary cultures of rat cerebellar granule cells at day 2 were treated with 0.5 mM etoposide (VP-16) in serum-free medium for 15 min. The treated cells on 100 mm dishes were lysed with 750 µl of a buffer containing 1% Sarkosyl, 10 mM Tris-HCl (pH 7.5), 10 mM EDTA, and protease inhibitor mixture (Complete Mini, Roche) by passing through a 23G needle 10 times. Concentrated CsCl (7 M) was added to a final concentration of 0.5 M. To fragment DNA, the lysates were sonicated with VP-5S sonicater (Taitec) for 30 sec at power setting 3. Under these conditions, length of DNA fragments, as determined by agarose gel electrophoresis, varied from 0.5 kb to 5 kb. Before immunoprecipitation (IP), 3 volumes of a buffer containing 10 mM Tris-HCl (pH 7.5), 10 mM EDTA, 100 mM NaCl, 0.1% Triton X-100, and protease inhibitor mixture was added to 1 volume of lysates and the diluted lysates were centrifuged for 15 min at 15,000 rpm at 4°C. The supernatant was pre-cleared with 10 µl of Dynabeads Protein G (DYNAL, Invitrogen) by rotating the tubes for 2 h at 4°C. Unbound fraction recovered by magnetic separation was used as an input for IP reactions. The reaction was initiated by the addition of 10 µl of Dynabeads protein G, which had been pre-incubated with 5 µg of specific antibody (3B6) or control antibody (mouse IgG). The beads suspension was incubated overnight at 4°C and the IP fraction was recovered by magnetic separation followed by washing 3 times with TEST-150 (10 mM Tris-HCl: pH 7.5, 10 mM EDTA, 150 mM NaCl, 0.1% Triton X-100, protease inhibitor). The IP-beads were then eluted 3 times with 500 µl of TEST-500 (10 mM Tris-HCl: pH 7.5, 10 mM EDTA, 500 mM NaCl, 0.1% Triton X-100, protease inhibitor) by magnetic separation each time and the eluates were pooled (designated as P2 fraction). DNA fragments still bound on the beads (designated as P1 fraction) were treated with 50 µg/ml RNase A at 55°C for 30 min, 200 µg/ml Proteinase K at 55°C overnight, and purified by phenol/chloroform extractions followed by ethanol-precipitation with 10 µg of glycogen. The DNA in IP input and P2 fraction was also purified and DNA amounts in each fraction were determined by Quant-iT PicoGreen dsDNA Quantitation Kit (Molecular Probes, Invitrogen) according to the manufacture's protocol.

### eTIP control experiments

Nuclei were prepared from 2-week-old rat brain (without cerebellum) by the high-density sucrose method. By removing the cerebellar tissue, it was possible to eliminate topo IIα from the preparation [3]. Nuclear pellet was extracted at 5-µg nucleic acids/ml with the extraction buffer (20 mM Tris-HCl: pH 7.5, 300 mM NaCl, 140 mM 2-mercaptoethanol, 50 µg/ml BSA) on ice for 30 min. In the final volume of 20 µl, plasmid pUC18 (0.5 µg) was incubated with nuclear extract (2 µl) at 37°C in the topo II reaction buffer (50 mM Tris-HCl: pH 8.0, 120 mM KCl, 10 mM MgCl_2_, 0.5 mM DTT, 0.5 mM EDTA, 0.5 mM ATP, 30 µg/ml BSA). In some reactions, 0.5 mM etoposide was added. After 15 min, the reaction was terminated either by Sarkosyl-CsCl (1% Sarkosyl, 0.5 M CsCl, 10 mM EDTA, 10 mM Tris-HCl: pH 7.5, 1× Complete mini) or SDS (1% SDS, 10 mM EDTA, 50 mM Tris-HCl: pH 8.0, 1× Complete mini). Samples were digested with SDS/Proteinase K and subjected to electrophoresis in 1% agarose gel in the presence of 0.5 µg/ml ethidium bromide.

For immunoprecipitation, supercoiled pUC18 DNA (7.5 µg) was incubated with the nuclear extract at 37°C in the presence of 0.5 mM etoposide (300 µl reaction volume). After 15 min, equal volume of 2× stop solution (20 mM Tris-HCl, pH 7.5, 2% Sarkosyl, 20 mM EDTA, 2× Complete mini) was added and incubated on ice for 15 min, then 7 M CsCl was added to a final concentration of 0.5 M. Before immunoprecipitation, 3 volumes of dilution buffer (10 mM Tris-HCl, pH 7.5, 10 mM EDTA, 100 mM NaCl, 1× Complete mini) were added. Immunoprecipitation procedures were the same as in eTIP. Purified DNA fractions (IP input, IP, P1 and P2) were analyzed by agarose gel electrophoresis as described above.

Cultured granule neurons were treated with etoposide and eTIP fractions (IP input, IP, P1, and P2) were prepared as described in the eTIP procedure. Protein samples were subjected to Western blotting to examine the presence of topo IIβ. Before electrophoresis, DNAs cross-linked to topo IIβ were digested with RNase-free DNase I in a buffer supplemented with 10 mM MgCl_2_. After incubation at 30°C for 45 min, 0.5 M EDTA was added to a final concentration of 15 mM to stop the reaction. Proteins in these fractions were recovered by acetone precipitation with 2 µg of BSA as a carrier and subjected to 6.5% SDS-PAGE and immunoblotting with topo IIβ-specific antibody (3B6).

### Tiling array

Purified DNA fractions (IP input, P1 and P2) were digested with *Mbo*I and amplified by ligation-mediated PCR (LM-PCR) performed as described [32] with minor modifications in that TaKaRa LA Taq (Takara Bio) was used for PCR amplification of 26 cycles. Designing of oligonucleotide probes and preparation of arrays were performed by NimbleGen. We selected 7 chromosomal regions for the analysis: chr2:91,018,106 -98,323,667; chr2:217,827,330-227,366,232; chr4:42,866,895-57,916,607; chr4:135,995,247-150,677,540; chr5:119,800,001-126,350,000; chr10:20,098,221-34,042,054; chr19:1-12,000,000. Rat DNA sequences (version 3.1, produced by the Atlas group at Baylor Human Genome Sequencing Center (HGSC) as part of the Rat Genome Sequencing Consortium) were retrieved using the UCSC Genome Browser (http://genome.ucsc.edu). The arrays were tiled with isothermal 50 to 75-mer oligos that were placed at 1 probe/100 bp on repeat-masked genomic sequence. Hybridization and scanning of the fluorescence intensities were performed at NimbleGen (http://www.nimblegen.com/). Briefly, amplified DNA preparations were labeled with Cy5 (P1 and P2) or Cy3 (IP input) and were hybridized competitively to the array with Cot-1 DNA. Scanned data were processed by the supplier's standard protocol for ChIP-chip analysis to obtain the scaled log_2_ (Cy5/Cy3 ratio).

The log_2_ (P1/input) and log_2_ (P2/input) values were used for the computational analysis named ACME (Algorithm for Capturing Microarray Enrichment) [33] to identify genomic sites enriched for topo IIβ action sites. The window size was set to 1,000 bp and the threshold to 90%. We calculated the probability values based on hypergeometric distribution instead of chi-square distribution, which is used in the original ACME. The array probes associated with probability values smaller than 10^−4.5^ were adopted as toposites. To classify the toposites, significant P1 signals were subjected to cluster analysis (Ward's method) between two factors, log_2_ (P2/input) and local GC content around toposites. Toposites were clearly separated into two clusters of comparable size: low P2 signal-high GC content (class 1) and high P2 signal-low GC content (class 2). The tiling array data were deposited in GEO (GSE13145).

We scanned the distribution of class 1 (c1) and class 2 (c2) toposites along the genome by using a sliding-window algorithm. With respect to the probes that were recognized as a toposite by ACME, the likelihood of being toposite was split and distributed evenly to every nucleotide across the probe. Namely, the inverse of probe length in number of nucleotides was defined as a “positional toposite potential” assigned to every nucleotide position within the toposite probe. To assess the toposite density around the transcription start sites (TSS) and termination sites (TTS) of 318 transcription units located in the tiled region, toposite potentials within ±10 kb from these sites were counted. The potential scores were then added up in every gene groups of interest that were aligned at TSS or TTS. Resulting figures were divided by the number of genes constituting the group in order to enable the comparison between different gene groups. Entire region of the 20 kb-potential map was scanned with a moving window of 1-bp step and 1,000-bp width. Sum of the potential scores within the window was plotted at the centre position as a toposite density. The highest density score would be 10 by definition. For a control, the same numbers of toposites were picked up randomly as Monte Carlo simulation of 10-times repetition and the toposite density was calculated as stated above.

### Shotgun cloning and sequencing

The eTIP DNA fractions (P1 and P2) amplified by LM-PCR were blunt-ended with T4 DNA polymerase (New England Biolabs), ligated into the *Eco*RV site of a zero-background cloning vector pZErO-2.1 (Invitrogen) and transformed into *Escherichia coli* TOP10F' competent cells (Invitrogen). The transformants were screened by colony PCR using AmpliTaq Gold DNA polymerase (Applied Biosystems) with M13RV and M13(-21) vector primers. The nucleotide sequences of eTIP clones were determined by direct sequencing of the amplified fragments on the DNA sequencer 310 according to standard methods (Applied Biosystems). Genomic positions of resulting sequences were determined on the rat genome (rn3) by BLAT search performed at the UCSC genome browser website (http://genome.ucsc.edu). The complete list of eTIP clones is given in Table S1.

## Supporting Information

Text S1 Glossary of unconventional words and phrases
(0.03 MB DOC)

Data S1 Supplementary data containing Figures S1, S2, S3, S4, S5, S6, S7, S8, S9, S10, S11 and Tables S1, S2, S3, S4
(2.81 MB PDF)

Figure S1 Confirmation of the array-based gene grouping by RT-qPCR and immunoblotting
(2.58 MB TIF)

Figure S2 Suppression of transcriptional induction of A1 genes by topo ΙΙβ siRNA
(1.51 MB TIF)

Figure S3 Schematic representation of the procedure for construction of exRefSeq
(1.36 MB TIF)

Figure S4 Classification of rat subgenomic regions by length and GC content
(3.02 MB TIF)

Figure S5 Comparison of expression groups in terms of gene's position, length, and GC content
(1.12 MB TIF)

Figure S6 Functional similarity between A1 genes and LA genes as revealed by a GO matrix
(1.70 MB TIF)

Figure S7 Prediction of A1 genes from positional and functional information
(1.65 MB TIF)

Figure S8 Characterization of eTIP DNA fractions by shotgun cloning and sequencing
(1.57 MB TIF)

Figure S9 Overview of the topography of toposites and genes in the seven chromosomal regions analyzed by tiling arrays
(7.31 MB TIF)

Figure S10 Similarity of expression patterns and genomic locations of relevant gene groups between embryonic brain and cultured granule cells
(1.45 MB TIF)

Figure S11 A high incidence of LA genes in monoallelically expressed autosomal genes
(1.10 MB TIF)

Table S1 Genomic location of eTIP DNA clones
(0.10 MB XLS)

Table S2 eTIP PCR primers
(0.03 MB XLS)

Table S3 RT-qPCR primers
(0.03 MB XLS)

Table S4 Antibodies used for Western blotting
(0.02 MB XLS)

Table S5 Gene Ontology (GO) analysis
(5.98 MB XLS)
