# Supplementary material for: Topoisomerase IIβ Activates a Subset of Neuronal Genes that Are Repressed in AT-Rich Genomic Environment
Source: PLoS One. 2008 Dec 31;3(12):e4103. doi: 10.1371/journal.pone.0004103 (PMC2605559; doi:10.1371/journal.pone.0004103)
Supplement: Text S1 — Glossary of unconventional words and phrases (0.03 MB DOC) [file pone.0004103.s001.doc]

**Glossary of Unconventional Words and Phrases**

Numbers of arbitrary terms or abbreviations are used in the present study. Although these are defined in each context, here we reiterate or rephrase them in a single list to alleviate possible confusions.

**Gene groups classified by expression profiling**

Levels of mRNA in cultured granule neurons were analyzed by expression arrays and the data were compared between undifferentiated (day 1) and differentiated (day 5) states in the presence or absence of a topo II-specific inhibitor (ICRF-193). The array data were plotted as a scattered graph of fold-induction versus fold-inhibition and the corresponding genes were classified into 9 groups by applying 2-fold and 0.5-fold boundary lines (Figure 1B). Groups A1, A2, B2, and unexpressed genes (group D) comprised about 90% of analyzed genes (Figure 1C and Figure S5A). The group A1 that makes up only 2.6% is the primary target of the present study since these genes are up-regulated depending on topo IIβ. A2 genes are also up-regulated but are not susceptible to the topo II inhibitor. B2 genes, the largest group, are expressed constitutively and independently of topo IIβ. Other groups were not analyzed further in this study. In some experiments, however, genes without assigned array probes (group N) were subjected to expression analysis by RT-qPCR.

**exRefSeq**

A rat genome compilation constructed by combining the sequence data of rat, mouse, and human RefSeq genes (Figure S3). After several steps of matching and cleaning, overlapping transcription units with the same gene name, genomic position, and the direction of transcription were unified into a longest hypothetical transcript and the resulting compilation of 17,799 rat genes was named exRefSeq for “extended RefSeq”.

**Classification of subgenomic regions**

Using the rat exRefSeq, genic region was first defined as a longest stretch of overlapping transcripts of protein-coding genes. The rat genome was then divided into genic and intergenic regions. They were further classified into four classes each by regional length and GC content (Figure S4). These factors have a certain biological relevance because the genomic distribution of topo IIβ action sites showed a strong correlation with regional GC content. A simple dual dichotomic approach was used here by employing the average regional length and overall GC content as boundary values. Although this scheme is apparently arbitrary, we found it satisfactorily sound in an empirical sense, after trying other statistical parameters. Thus, the boundary values adopted were 51 kb / 44% GC for genic region and 110 kb / 42% GC for intergenic region. The 4 areas demarcated by the boundary lines in the scatter plot are designated as LA (Long AT-rich), LG (Long GC-rich), SA (Short AT-rich), and SG (Short GC-rich). Accordingly, classified subgenomic regions are referred to as LAGR (Long and AT-rich Genic Region), LGGR (Long and GC-rich Genic Region), SAGR (Short and AT-rich Genic Region), SGGR (Short and GC-rich Genic Region) for genic regions (GR), and LAIR (Long and AT-rich Intergenic Region), LGIR (Long and GC-rich Intergenic Region), SAIR (Short and AT-rich Intergenic Region) and SGIR (Short and GC-rich Intergenic Region) for intergenic regions (IR).

**LAIR, LAIR-proximal and LAIR-distal genes**

By definition LAIR stands for Long (110 kb <) and AT-rich (< 42% GC) Intergenic Region. In the present study we regard LAIR as a genomic element imposing a suppressive effect on neighboring genes, not just an intergenic region. LAIR is likely to overlap and share properties with other genomic regions described as “gene desert” and “lamina-associated domain (LAD)” (see Discussion). Genes positioned right next to LAIR is designated LAIR proximal (LAIRp) and other genes are all LAIR distal (LAIRd). Genes classified into expression groups (A1, A2, B2, D) are discriminated similarly by the suffix designating their positions relative to LAIR (as in A1p and A1d).

**LA gene**

LA gene is defined as a Long (51 kb <) and AT-rich (< 44% GC) gene adjacent to LAIR (in other words, LAIR-proximal LAGR). It appears to be well conserved in mammals when the same criteria for the boundary values were applied to other genomes. LA genes show remarkable bias towards membrane/cell surface proteins. Many of them are essential for nerve cell development or mature neuronal functions. We propose that LA genes are a distinct category of genes and topo IIβ liberates them from the transcriptional repression exerted by juxtaposed LAIR. Merely long and AT-rich genes should not be confused with LA genes that are exclusively LAIR-proximal genes. LA genes share a strong functional similarity with A1 genes that are controlled by topo IIβ (Figure S6). A high incidence of LA genes in the monoallelically expressed gene groups (Figure S11) and in the genes associated with autism and schizophrenia suggests that their stochastically determined monoallelic expression would cause a cellular mosaicism and may explain a phenotypic polymorphism of psychotic conditions or even personality variations in normal individuals. Thus, LA genes may serve as a useful set of candidate genes for the search of psychosis-related genes. We hypothesize that LA genes and their control machinery evolved relatively recently in the evolutionary history of vertebrates and are involved in higher functions of central nervous system. This category of genes will be important in elucidating human evolution as well as etiology of psychosis.

**G- and T-segments**

Type II DNA topoisomerases catalyze the strand passage events between the two segments of DNA, termed G- and T-segments (representing Gate and Transfer). The G-segment contains the transient gap generated by the enzyme for the passage of T-segment. For the reaction to occur, the two segments should come close each other, which is easily achieved in circular DNAs with superhelical turns. In this intramolecular reaction, both segments of DNA reside in the same molecule within a short distance in the primary sequence, as well. In theory, however, these segments can be vastly separated as long as they can make a close contact in the 3-dimensional nuclear space. Although this type of reaction is unique to type II topoisomerases and must be important in living cells, the *in vivo* situation has been largely unknown, due to the lack of technique capable of discriminating the G-segment and T-segment.

**eTIP**

The novel technique eTIP (etoposide-mediated topoisomerase immunoprecipitation) can locate direct action sites of topo IIβ on DNA, not just binding sites (Figure 3A). The enzyme-DNA covalent complex stabilized by etoposide *in vivo* was recovered by lysing the cells in mild conditions without strong denaturants. The enzyme-DNA intermediate is converted mainly to a form with a single-strand breakage at the site of action. After fragmentation of chromosomal DNA, the topo IIβ -DNA complex was concentrated by immunoprecipitation with specific antibody. Resulting DNA fragments were amplified by ligation-mediated PCR and hybridized to tiling arrays to determine their genomic positions. The use of mild conditions for arresting the intermediate brings about two advantages. First, as topo IIβ is linked to DNA mostly through single strand breakage, the cleavage site resides within the fragment and both ends are available for ligation of amplification primers. Second, the other DNA strand, which is transferred through the cleaved strand (T-segment), remains associated with the enzyme in the immunoprecipitate. When it is not contiguous to G-segment, the T-segment can be recovered in a separate fraction by a stringent washing. The recovery of T-segment is a key component of successful identification of distinctive classes of topoisomerase action sites.

**eTIP DNA fractions P1 and P2**

In the eTIP procedure, DNA fragments immunoprecipitated with topo IIβ was fractionated by high salt wash (0.5 M NaCl) into eluted DNA (P2) and residual DNA, which is still bound to the enzyme (P1). Relationship between G-segment vs. T-segment, P1 fraction vs. P2 fraction, and class 1 toposite (c1) vs. class 2 toposite (c2) is very confusing. The illustration shown in Figure 4D should be useful to clarify the situation.

**Toposites c1 and c2**

“Toposites” stand for topoisomerase action sites detected by the eTIP-tiling array analysis. Comparison of hybridized signals for P1 and P2 DNA fractions on genomic tiling arrays revealed the presence of two classes of distinctive toposites (c1, c2) that clearly differs in local GC contents. Toposites are compared schematically in Figure 6A. When mapped positions of toposites are examined, it should be noted that c2 sites on the map are basically half sites as they always have paring sites elsewhere distant in the genome. The GC-rich c1 toposites are enriched in GC-rich genic regions, whereas AT-rich c2 toposites are concentrated in LA genes and adjacent LAIR. Thus, c2 sites are likely to be essential for the regulated expression of LA genes while LAIR-distal A1 genes appear to be regulated by c1 sites. Although we focused on c2 sites and LA genes in the present study, roles of c1 sites in the control of nonLA genes are less clear at present and should be addressed in future studies. The c2 site also suggests the presence of interaction between distant genomic sites, which is to be modulated by the enzyme. This view certainly expands the ascribed roles of type II DNA topoisomerases into an emerging picture of transcriptional regulation through distant chromosomal interactions.
