# Supplementary material for: Topoisomerase IIβ Activates a Subset of Neuronal Genes that Are Repressed in AT-Rich Genomic Environment
Source: PLoS One. 2008 Dec 31;3(12):e4103. doi: 10.1371/journal.pone.0004103 (PMC2605559; doi:10.1371/journal.pone.0004103)
Supplement: Data S1 — Supplementary data containing Figures S1, S2, S3, S4, S5, S6, S7, S8, S9, S10, S11 and Tables S1, S2, S3, S4 (2.81 MB PDF) [file pone.0004103.s002.pdf]

Supplementary Data

Topoisomerase IIβ Activates a Subset of Neuronal Genes That Are Repressed in AT-rich Genomic Environment

Kuniaki Sano[1], Mary Miyaji-Yamaguchi[1], Kimiko M. Tsutsui[1] & Ken Tsutsui[2]

[1]Department of Neurogenomics, [2]Department of Genome Dynamics, Graduate School of Medicine, Dentistry and Pharmaceutical Sciences, Okayama University, Okayama, Japan

Contents

Figure S1. Confirmation of the array-based gene grouping by RT-qPCR and immunoblotting
Figure S2. Suppression of transcriptional induction of A1 genes by topo IIβ siRNA
Figure S3. Schematic representation of the procedure for construction of exRefSeq
Figure S4. Classification of rat subgenomic regions by length and GC content
Figure S5. Comparison of expression groups in terms of gene's position, length, and GC content
Figure S6. Functional similarity between A1 genes and LA genes as revealed by a GO matrix
Figure S7. Prediction of A1 genes from positional and functional information
Figure S8. Characterization of eTIP DNA fractions by shotgun cloning and sequencing
Figure S9. Overview of the topography of toposites and genes in the seven chromosomal regions analyzed by tiling arrays
Figure S10. Similarity of expression patterns and genomic locations of relevant gene groups between embryonic brain and cultured granule cells
Figure S11. A high incidence of LA genes in monoallelically expressed autosomal genes

Table S1. Genomic location of eTIP DNA clones
Table S2. eTIP PCR primers
Table S3. RT-qPCR primers
Table S4. Antibodies used for Western blotting

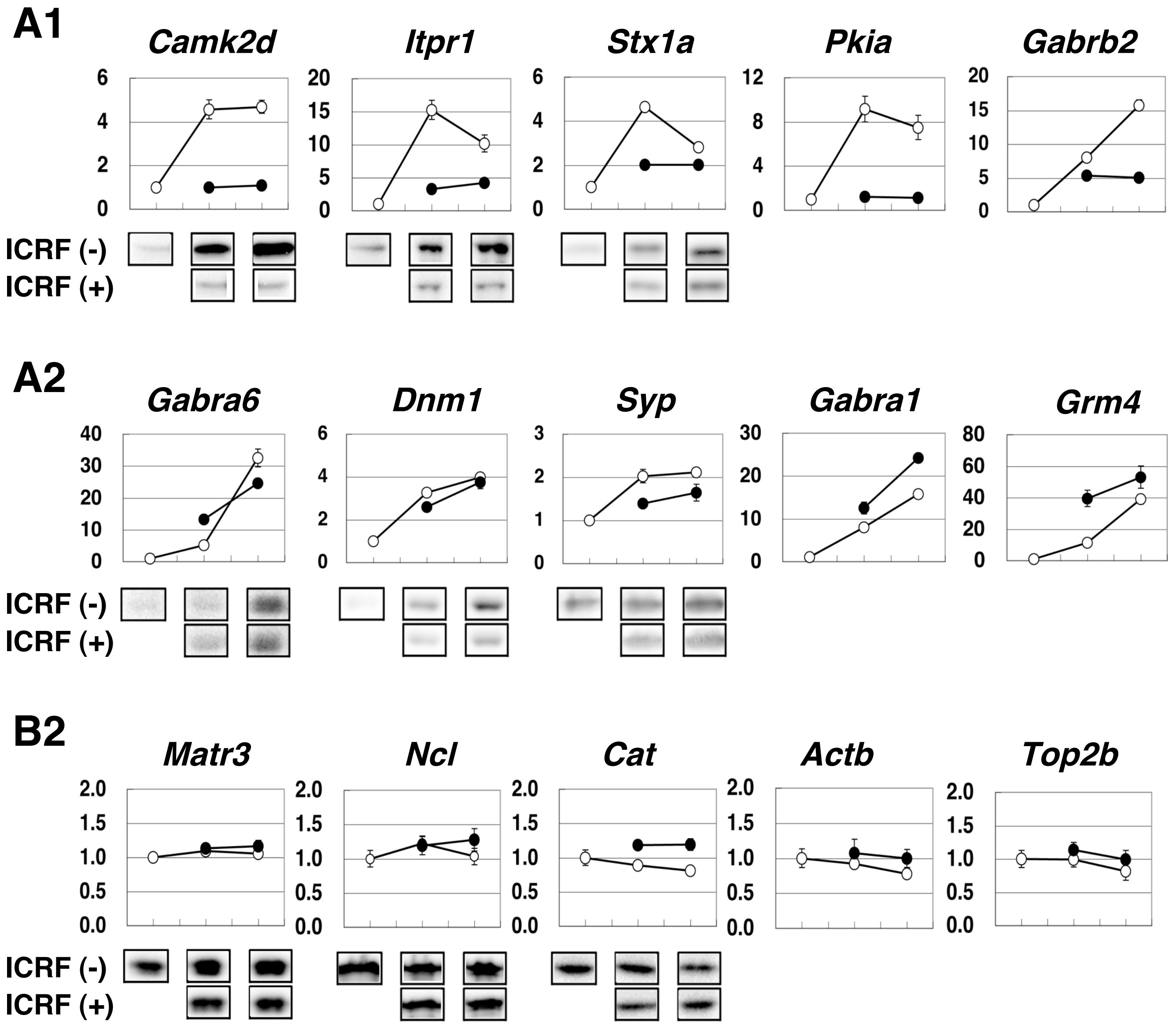

Figure S1. Confirmation of the array-based gene grouping by RT-qPCR and immunoblotting. RNA samples were prepared from the granule cells in culture at day 1, day 3, and day 5 in the presence (filled circles) or absence (open circles) of 10 μM ICRF-193. Total RNA was used as a template for the cDNA synthesis with M-MLV reverse transcriptase and primer (random hexamer). Representative genes (5 each) from A1, A2, and B2 gene groups that were classified by the expression array experiments were subjected to RT-qPCR analysis to estimate their transcript levels. Constant amounts of cDNA (equivalent to 15 ng of template RNA) were used for qPCR amplification and relative copy numbers of the product with respect to day 1 are plotted (mean ± s.d., $n = 3$). Sequences of primer pairs used for the amplification are listed in Table S3. Protein levels of some gene products, for which antibody is available, were also estimated by immunoblotting (antibodies and dilutions are given in Table S4). Gene names: ***Camk2d***, calcium/calmodulin-dependent protein kinase type IIδ; ***Itpr1***, inositol 1,4,5-trisphosphate receptor type 1; ***Stx1a***, syntaxin 1A; ***Pkia***, cAMP-dependent protein kinase inhibitor, α form; ***Gabrb2***, gamma-aminobutyric acid receptor ($GABA_A$), β2 subunit; ***Gabra6***, gamma-aminobutyric acid receptor ($GABA_A$), α6 subunit; ***Dnm1***, dynamin 1; ***Syp***, synaptophysin; ***Gabra1***, gamma-aminobutyric acid receptor ($GABA_A$), α1 subunit; ***Grm4***, metabotropic glutamate receptor 4; ***Matr3***, matrin 3; ***Ncl***, nucleolin; ***Cat***, catalase; ***Actb***, beta-actin; ***Top2b***, DNA topoisomerase IIβ.

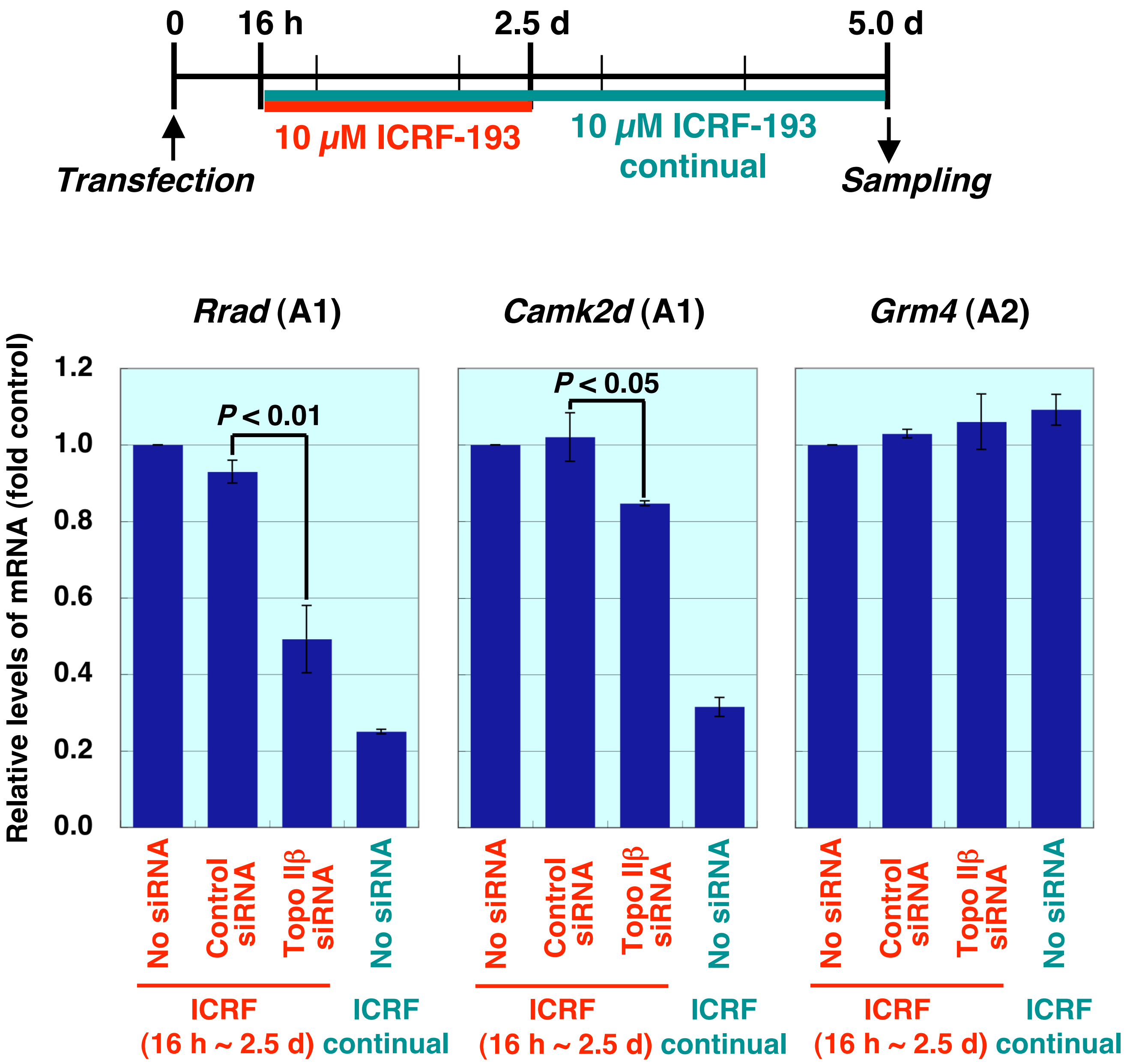

Figure S2. Suppression of transcriptional induction of A1 genes by topo IIβ siRNA. Effects of topo IIβ siRNA on mRNA levels of *Rrad* (group A1), *Camk2d* (group A1), and *Grm4* (group A2) genes were examined. Because of the delayed action of siRNA, it was mandatory to use ICRF-193 in the early phase of culture (until 2.5 days) in order to observe the RNAi effect. Since the drug effect is completely reversible, removal of the drug increases the transcript to a native level by day 5 (data not shown). As a positive control, ICRF-193 was added continuously to the culture. At the culture start, siRNAs were transfected to dispersed cerebellar granule cells. ICRF-193 was added after 16 h and removed after 2.5 days. RNA samples were prepared at day 5 and analyzed by RT-qPCR as in Figure S1. Levels of mRNA expression relative to the siRNA-minus control were plotted. Vertical bars designate mean ± s.d. (n = 3). Gene name: ***Rrad***: RAS associated with diabetes (*Rad1*); ***Camk2d***, calcium/calmodulin-dependent protein kinase type IIδ; ***Grm4***, metabotropic glutamate receptor 4. Note that the difference between control and topo IIβ siRNAs is statistically significant in A1 genes (*Rrad* and *Camk2d*) but not in A2 gene (*Grm4*).

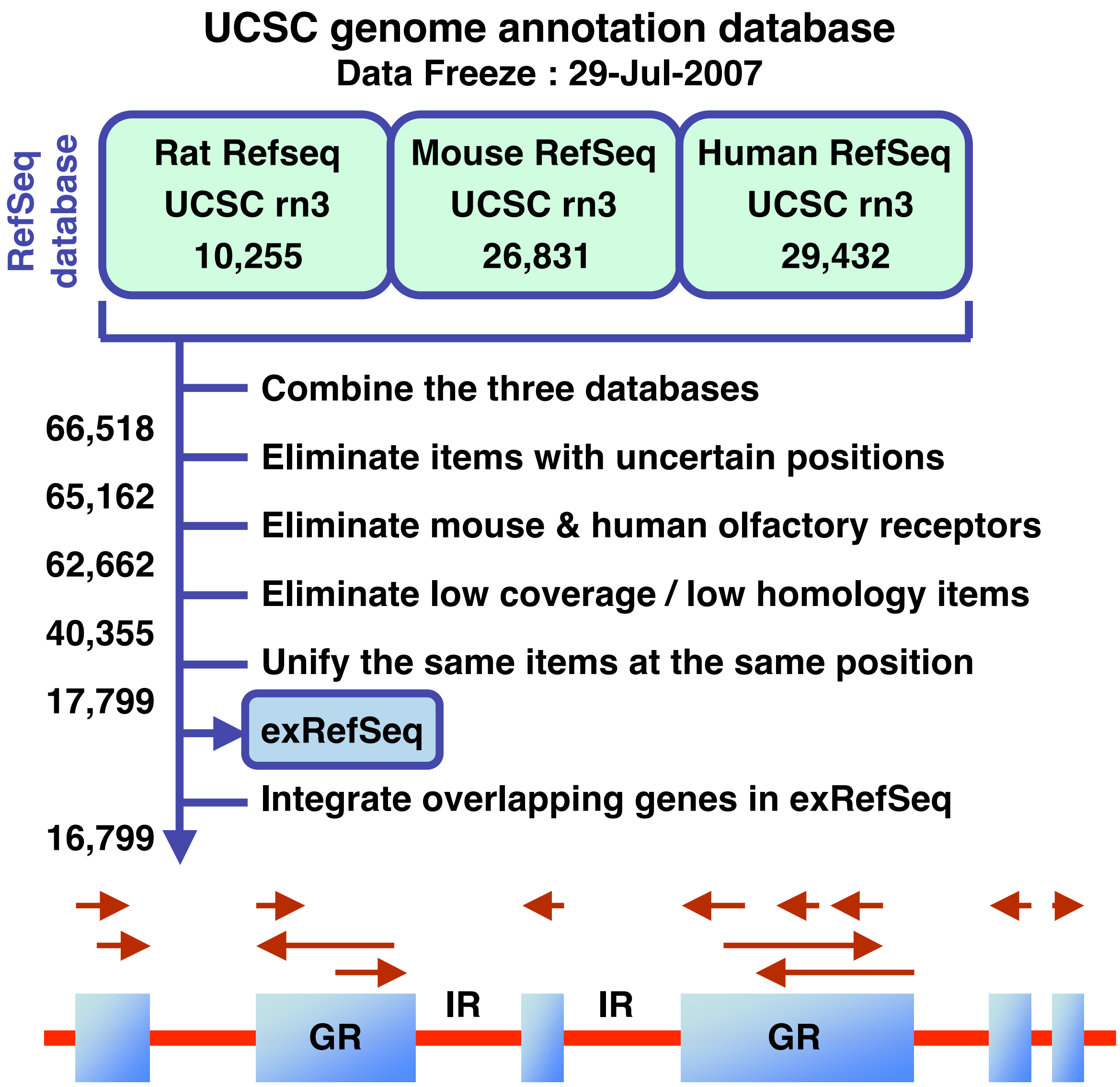

	Genic region (GR)	Intergenic region (IR)
Total number	16,799	16,820
Genome coverage	863 Mb (31.7%)	1,857 Mb (68.3%)

Figure S3. Schematic representation of the procedure for construction of exRefSeq. Using the UCSC genome annotation database (Data freeze: 29-Jul-2007) as the main source, the coordinates for rat, mouse, and human RefSeq were first integrated into one, and then multiple sifting and cleaning steps were applied to construct a rat genome compilation termed exRefSeq. As illustrated here, the genic region was defined as a longest stretch of overlapping transcripts (including reverse directions) in exRefSeq. In the present study, the word “genic” stands for transcribed regions of protein-coding genes. Numbers on the left of the vertical arrow stand for total numbers of remaining items at each step. Detailed description for this procedure is given in Materials and Methods.

	Genic	Intergenic
Number<br>Genome Coverage (%)<br>Average Length<br>GC content	16,799<br>863,065,498 bp (31.7)<br>51,376 bp<br>43.7%	16,820<br>1,856,856,955 bp (68.3)<br>110,396 bp<br>42.2%
Number / Coverage (%)		
Class: LA	2,234 / 428 Mb (13.3 / 49.6)	1,189 / 925 Mb (7.1 / 49.8)
LG	1,786 / 244 Mb (10.6 / 28.3)	1,834 / 600 Mb (10.9 / 32.3)
SA	3,749 / 66 Mb (22.3 / 7.7)	2,929 / 85 Mb (17.4 / 4.6)
SG	9,030 / 124 Mb (53.8 / 14.4)	10,868 / 246 Mb (64.6 / 13.3)

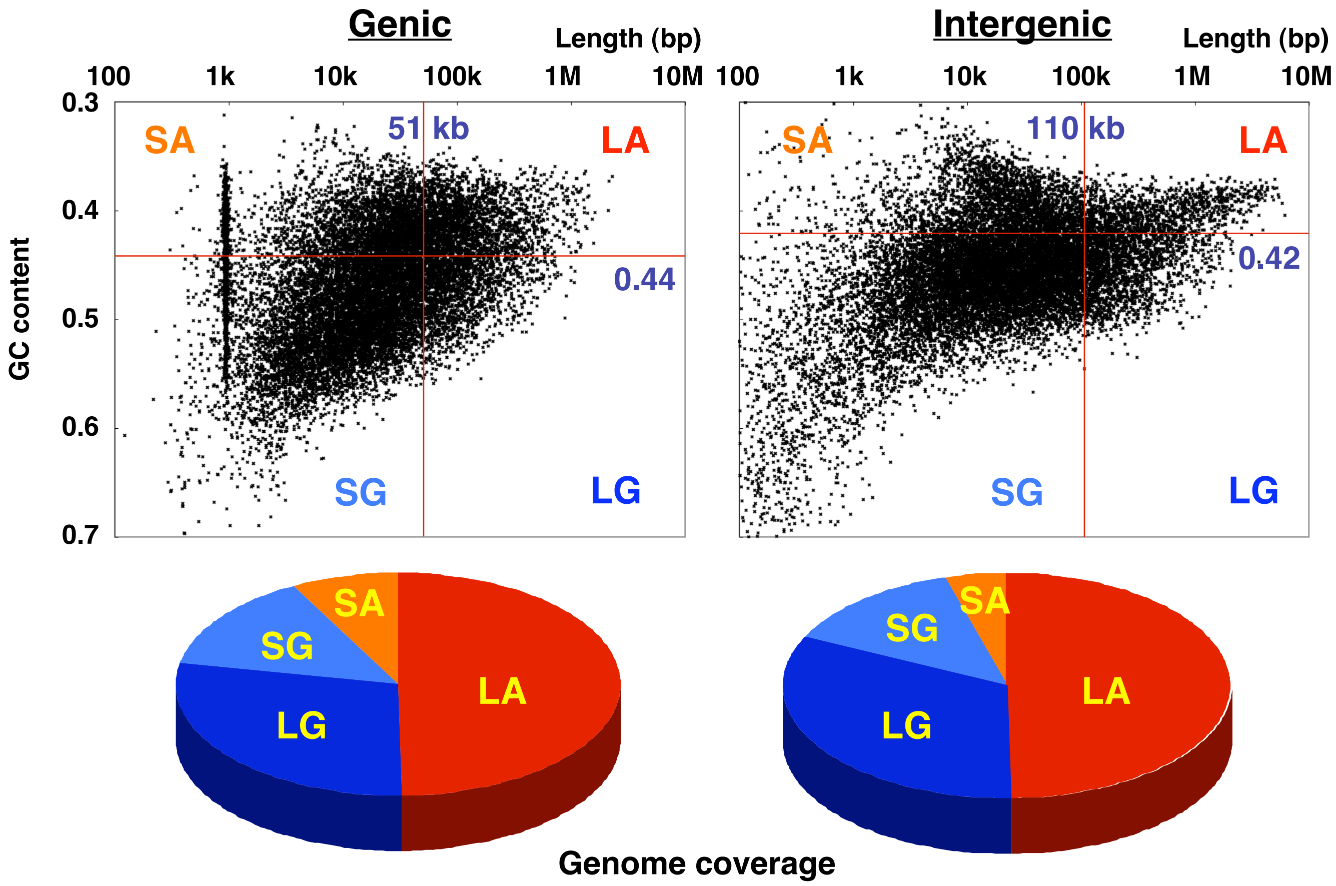

Figure S4. Classification of rat subgenomic regions by length and GC content. Genic and intergenic regions were classified into four classes by their average length (51 kb and 110 kb, respectively) and overall GC content (0.44 and 0.42, respectively). These classes are designated as LA (Long AT-rich), LG (Long GC-rich), SA (Short AT-rich), and SG (Short GC-rich). Accordingly, classified genic regions (GR) and intergenic regions (IR) will be referred to as LAGR, LGGR, SAGR, SGGR, LAIR, LGIR, SAIR and SGIR, respectively.

A

	Number (%)	LAIRp	LAIRd	LAGR	LAIRp & LAGR	LAIRd & LAGR
A1	327 (2.6)	77	250	87	61	26
A2	987 (7.8)	129	858	142	66	76
B2	7,084 (55.7)	521	6,563	929	217	712
D	2,918 (23.0)	309	2,609	292	175	117
Others	1,393 (11.0)	216	1,177	293	84	209
Total	12,709 (100)	1,252	11,457	1,743	603	1,140

B

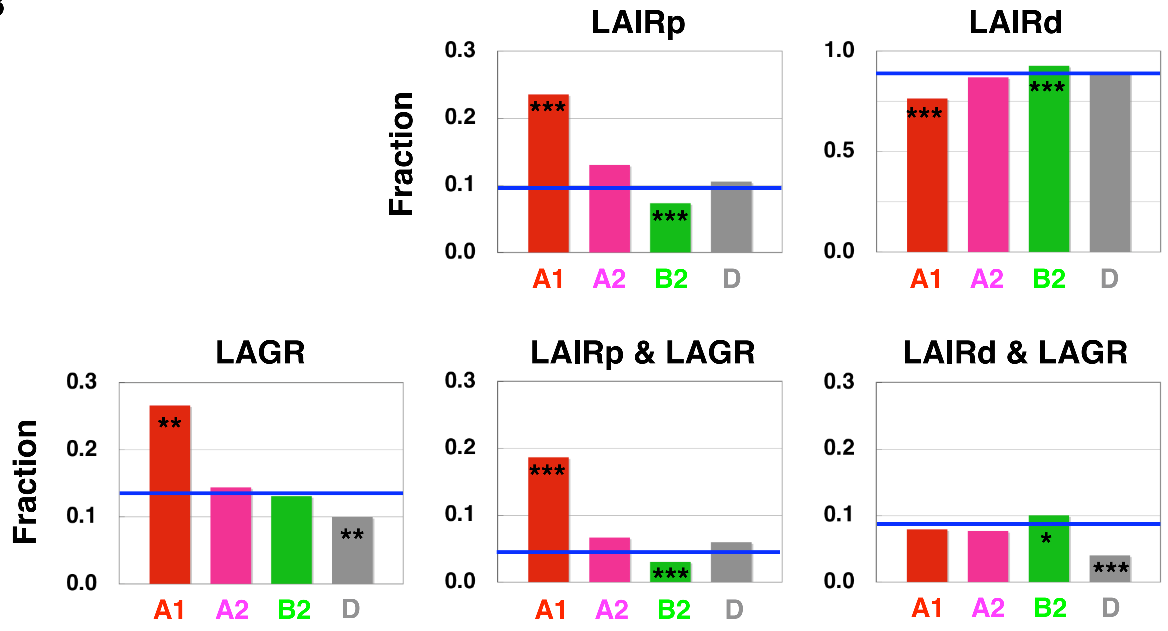

Figure S5. Comparison of expression groups in terms of gene's position, length, and GC content. A, The expression array data merged to exRefSeq were used to count the number of genes with features indicated in the table. LAIRp, LAIR-proximal; LAIRd, LAIR-distal; LAGR. Total genes in exRefSeq after subtracting the genes without assigned array probes (termed group N) were used for the analysis. "Others" stands for the sum of other minor groups (A3, B1, B3, C1, C2, C3, and Mix). **B**, Fractions of the grouped genes that belong to the featured categories are illustrated in the bar graphs. Levels for total genes are shown by blue lines, which were used as a base for the calculation of statistical significance by chi-square test. *$P < 10^{-5}$, **$P < 10^{-10}$, ***$P < 10^{-15}$.

A

	A1	A2	B2	D	A1p	A2p	B2p	Dp	A1d	A2d	B2d	Dd	LAIRp	LAIRd	LAGR	LA gene	Neuro
A1	38	1	0 [-36]	31	20	0	0	6	21	10	4 [-8]	36	16	0 [-22]	2	26	30
A2	1	16	0 [-10]	4	1	1	0	2	1	13	3 [-1]	7	1	0 [-1]	0	2	2
B2	0 [-7]	0 [-14]	115	0 [-76]	0 [-2]	0	0	0 [-8]	3	11 [-1]	104	4 [-11]	0 [-10]	8	8 [-2]	0 [-6]	3 [-8]
D	31	4	0 [-122]	174	17	1	0	27	28	29	15 [-25]	161	24	0 [-30]	2	31	67
A1p	20	1	0 [-22]	17	24	0	0	3	11	9	5 [-6]	22	13	0 [-17]	2	19	20
A2p	0	1	0 [-1]	1	0	1	0	1	0	1	0 [-1]	1	0	0	0	0	0
B2p	0	0	0	0	0	0	0	0	0	0	0	0	0	0	0	0	0
Dp	6	2	0 [-27]	27	3	1	0	28	5	8	2 [-10]	25	10	0 [-10]	0	8	17
A1d	21	1	3 [-29]	28 [-3]	11	0	0	5	33	19	12 [-3]	33	11	0 [-16]	0	19	28
A2d	10 [-4]	13 [-2]	11 [-44]	29 [-12]	9 [-2]	1	0	8 [-4]	19	101	58 [-1]	70	14 [-4]	4 [-17]	7 [-1]	19 [-4]	51 [-4]
B2d	4 [-7]	3 [-22]	104 [-18]	15 [-85]	5 [-2]	0	0	2 [-8]	12	58	541	109 [-7]	8 [-10]	8 [-9]	18 [-2]	7 [-6]	104 [-9]
Dd	36 [-1]	7	4 [-155]	161 [-5]	22	1	0	25 [-1]	33	70	109 [-17]	443	30 [-1]	1 [-37]	9	40 [-1]	164 [-1]
LAIRp	16	1	0 [-31]	24	13	0	0	10	11	14	8 [-9]	30	34	0 [-33]	5	29	27
LAIRd	0 [-6]	0 [-4]	8	0 [-8]	0 [-2]	0	0	0 [-8]	0	4	8	1	0 [-8]	8	0	0 [-6]	0 [-6]
LAGR	2	0	8 [-7]	2 [-12]	2	0	0	0	0	7	18	9 [-1]	5	0 [-5]	38	8	14
LA gene	26	2	0 [-40]	31	19	0	0	8	19	19	7 [-10]	40	29	0 [-36]	8	47	34
Neuro	30	2	3 [-83]	67 [-6]	20	0	0	17	28	51	104 [-15]	164	27	0 [-33]	14	34	302

B

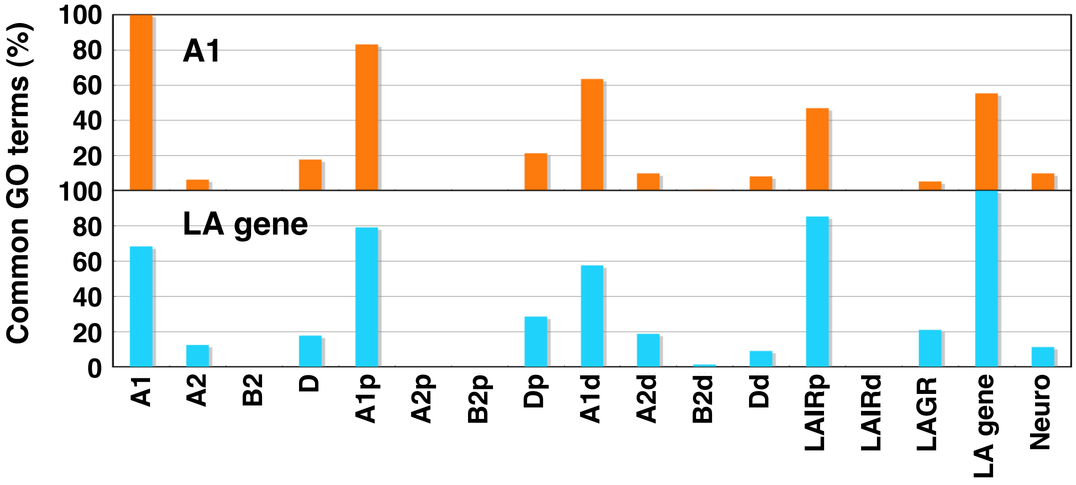

Figure S6. Functional similarity between A1 genes and LA genes as revealed by a GO matrix. **A**, A matrix composed of the numbers of overlapping GO terms was created. GO terms over-represented in the gene groups that were classified by expression or genomic locations were first retrieved by the GOstat program ($P < 10^{-4}$) and their numbers were placed on the diagonal position of the matrix (colored yellow). "Neuro" stands for the neuronal genes selected as described in Materials and Methods. Numbers of common GO terms in every combination of gene groups were then calculated and filled into the matrix at the corresponding non-diagonal positions. Numbers in brackets are under-represented terms. The complete list of GO terms can be found in Table S5. **B**, The entire matrix was converted to relative values by dividing the figures in every rows with the diagonal elements (resulting matrix not shown). The columns for A1 and LA gene (highlighted by orange and light blue, respectively) were plotted in bar graphs representing percentages of overlapping GO terms in various gene groups with respect to A1 gene (upper) or LA gene (lower). The overlap patterns are very similar each other, indicating that these gene groups are functionally similar. No such relationship was observed in other combinations.

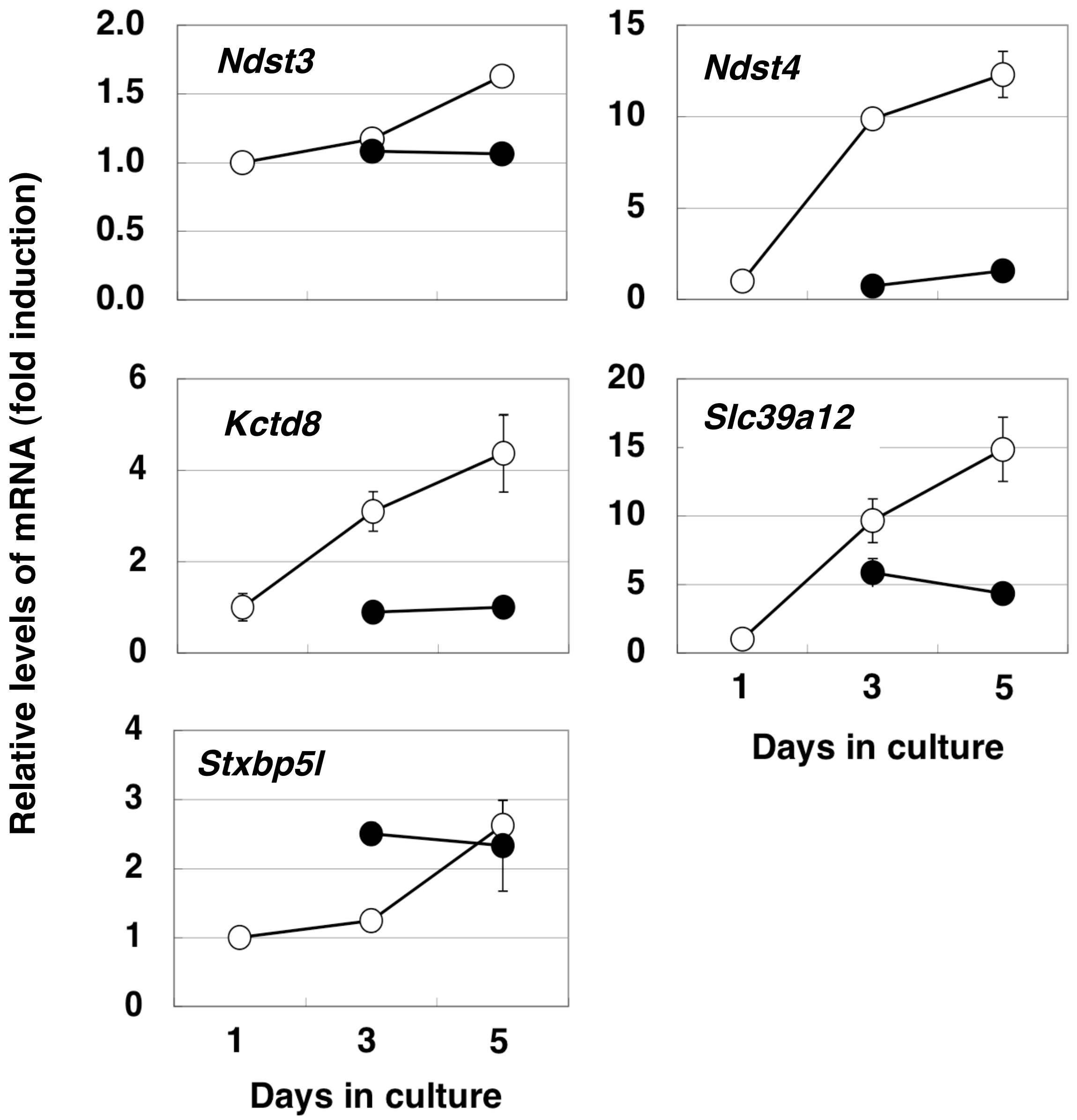

Figure S7. Prediction of A1 genes from positional and functional information. Expression kinetics of five A1 candidate genes selected from group N genes was examined by RT-qPCR using total RNA samples obtained from granule neurons cultivated in the presence (filled circles) or absence (open circles) of ICRF-193. Vertical bars designate mean ± s.d. (n = 3). The following criteria were employed for the selection of candidate genes. 1) LA gene (LAIR-proximal and LAGR), 2) Genes sharing at least 2 GO terms with A1 genes ($P < 10^{-3}$), 3) Genes whose expression is induced to a maximum level in cerebellar granule cells at 7-14 days after birth. To obtain this information, RIKEN Cerebellar Development Transcriptome Database (CDT-DB) was searched. Except for Stxbp5l, the genes tested here behave like group A1 as expected. It is worth noting that *Ndst3* and *Ndst4* are located on chromosome 2, flanking a long LAIR enriched with c2 toposites (see Figure S9 for the map). Gene names: ***Ndst3***, N-deacetylase/N-sulfotransferase (heparan glucosaminyl) 3; ***Ndst4***, N-deacetylase/N-sulfotransferase (heparan glucosaminyl) 4; ***Kctd8***, Potassium channel tetramerization domain containing 8; ***Slc39a12***, solute carrier family 39 (zinc transporter); ***Stxbp5l***, syntaxin binding protein 5-like.

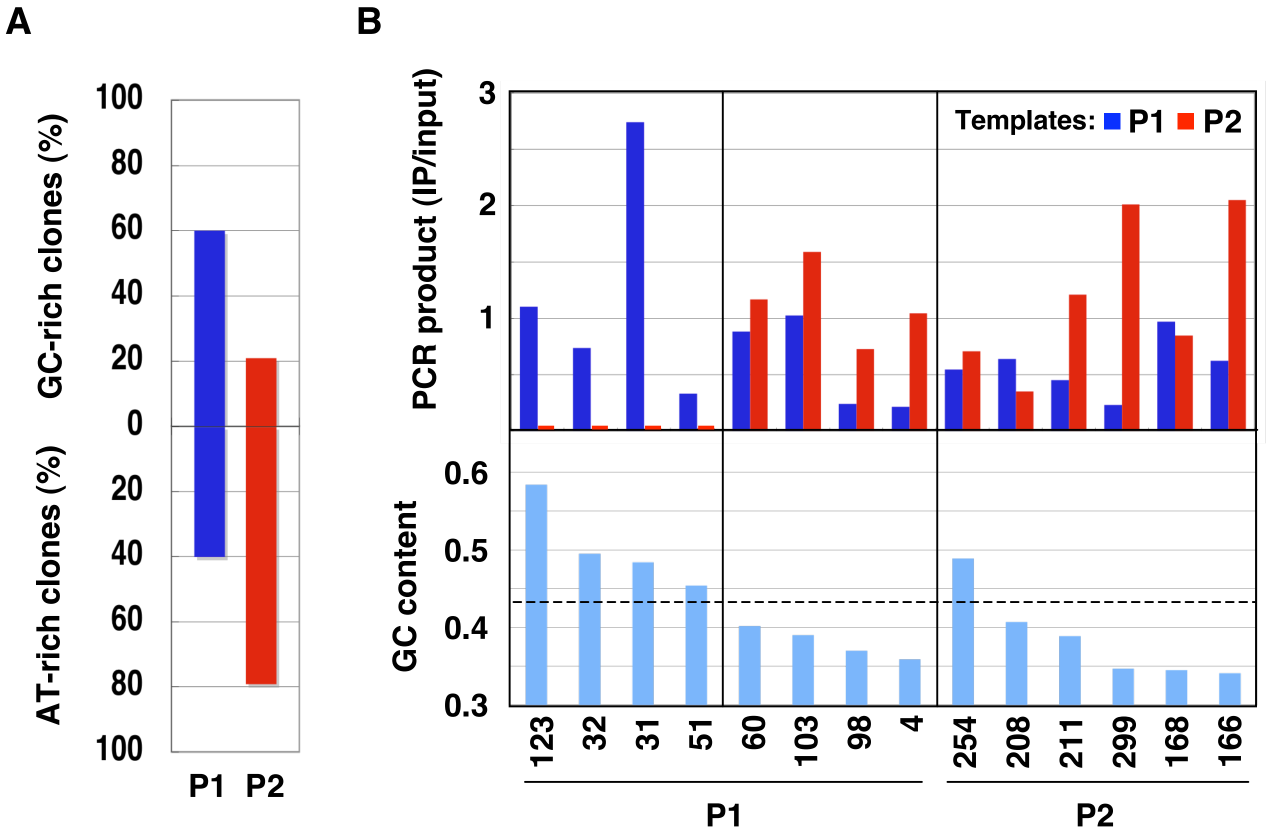

Figure S8. Characterization of eTIP DNA fractions by shotgun cloning and sequencing. The eTIP experiment was performed at the 2nd day of granule cell differentiation. DNA fragments purified from P1 and P2 fractions were cloned into a vector and sequenced as in Materials and Methods. The BLAT search identified 135 and 177 genomic locations for P1 and P2 DNA clones, respectively (complete data is presented in Table S1). **A**, Analysis of nucleotide compositions suggested that P1 DNA is composed of two different populations with distinct GC contents, whereas P2 DNA clones are mostly AT-rich ($P < 10^{-8}$). Local GC contents were calculated for 1,000-bp span (± 500 bp from the center of cloned region) and the numbers of clones with GC contents higher (GC-rich) or lower (AT-rich) than that of entire rat genome (0.427) were counted. The apparent enrichment of AT-rich sequences in P2 clones was estimated by chi-square test. **B**, PCR amplification of eTIP DNA fractions with primers complementary to the cloned fragments. Sequences for eTIP clones with various local GC contents were selected and used to design the primers (summarized in Table S2). Template DNAs from P1 and P2 fractions or IP input (before immunoprecipitation) were amplified with these primer sets. Relative amounts of PCR products for 8 clones from P1 and 6 clones from P2 are shown here. Numbers at the bottom stand for the clone number (listed in Table S1). Local GC contents calculated as above are plotted in the lower panel. Overall GC content of rat genome (0.427) is indicated by broken line. The results confirmed the notion stated above in retrograde: the GC-rich clones from P1 fraction were found exclusively in P1 template, whereas AT-rich clones from P1 or P2 were amplified with both P1 and P2 templates.

chr2:91,018,106-98,323,667

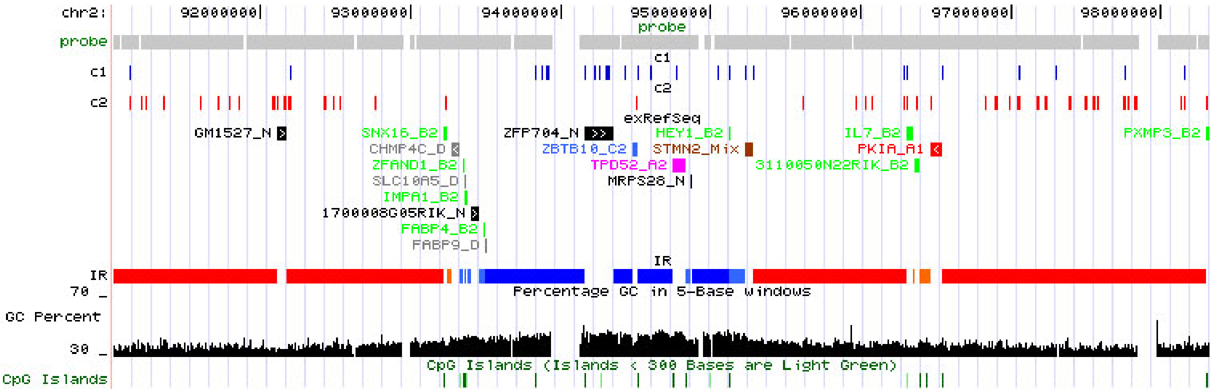

chr2:217,827,330-227,366,232

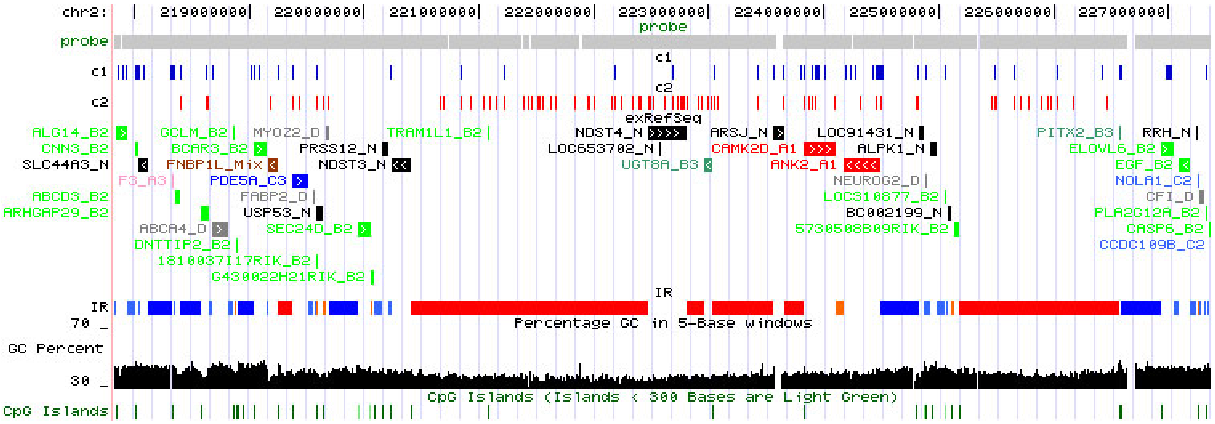

chr4:42,866,895-57,916,607

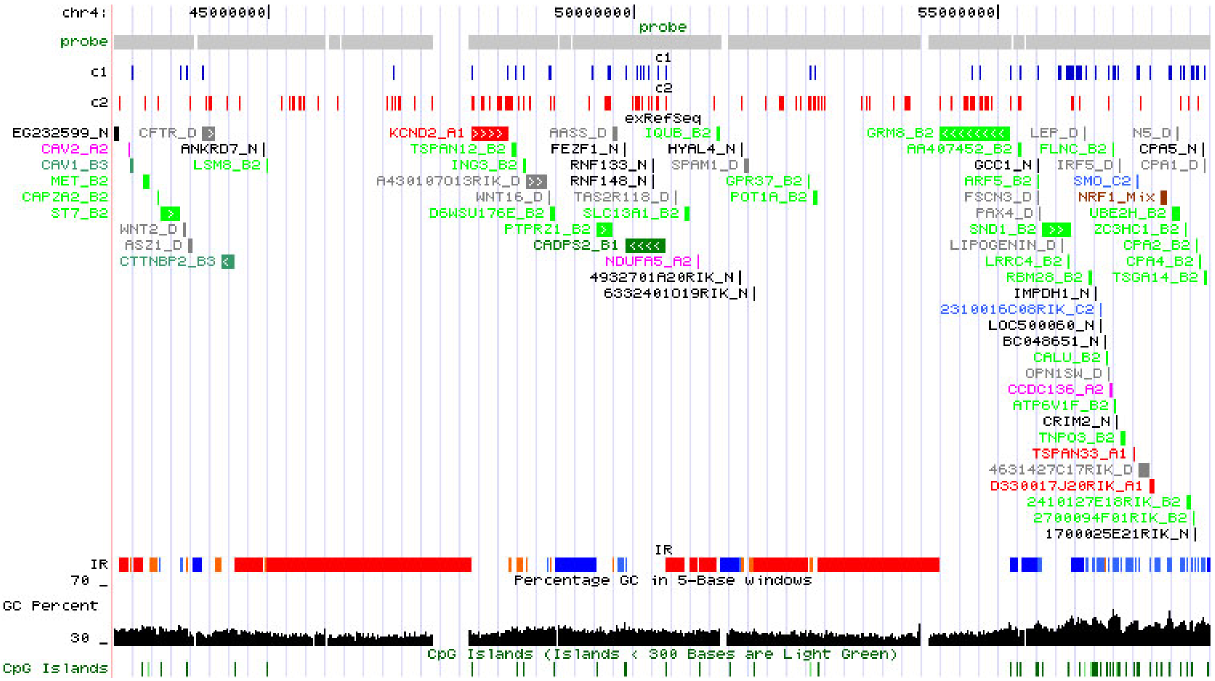

chr4:135,995,247-150,677,540

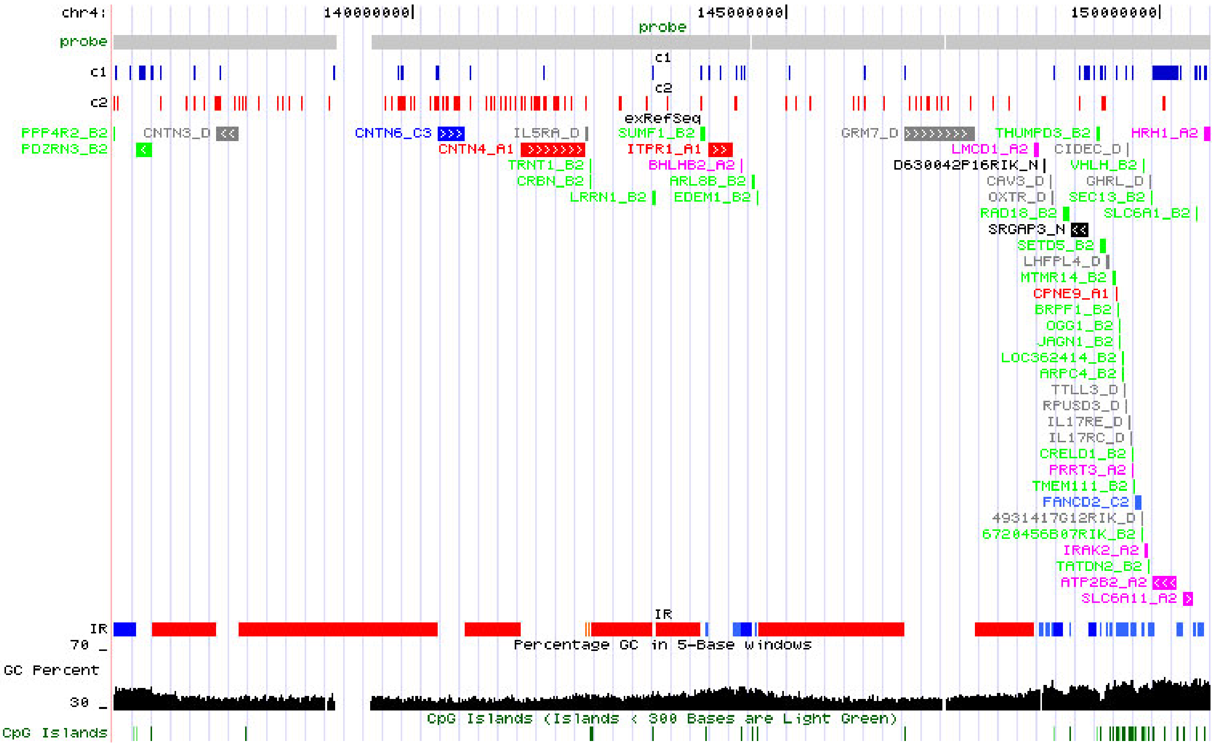

chr5:119,800,001-126,350,000

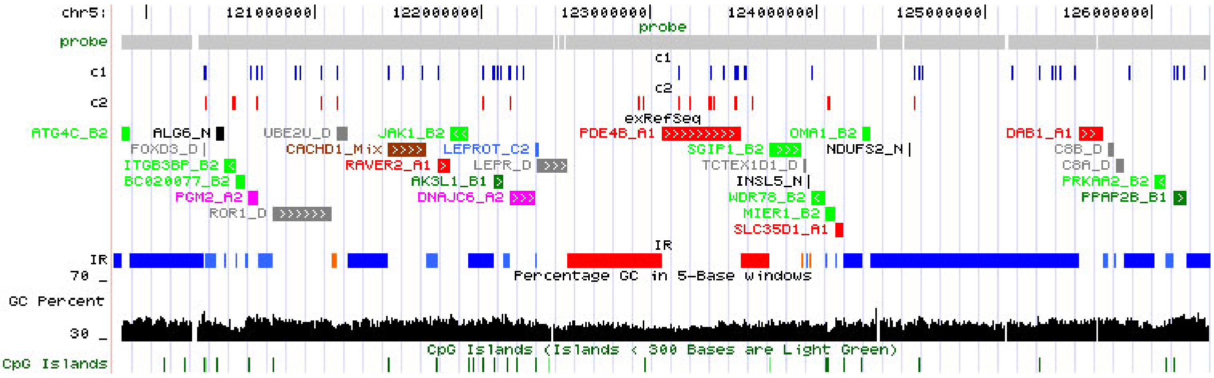

chr10:20,098,221-34,042,054

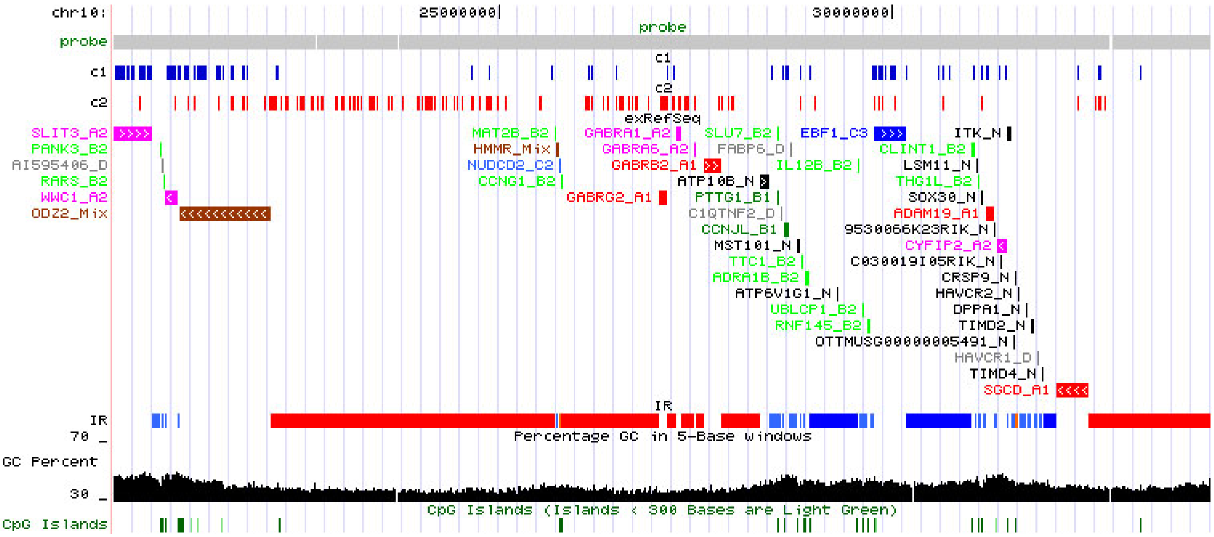

chr19:1-12,000,000

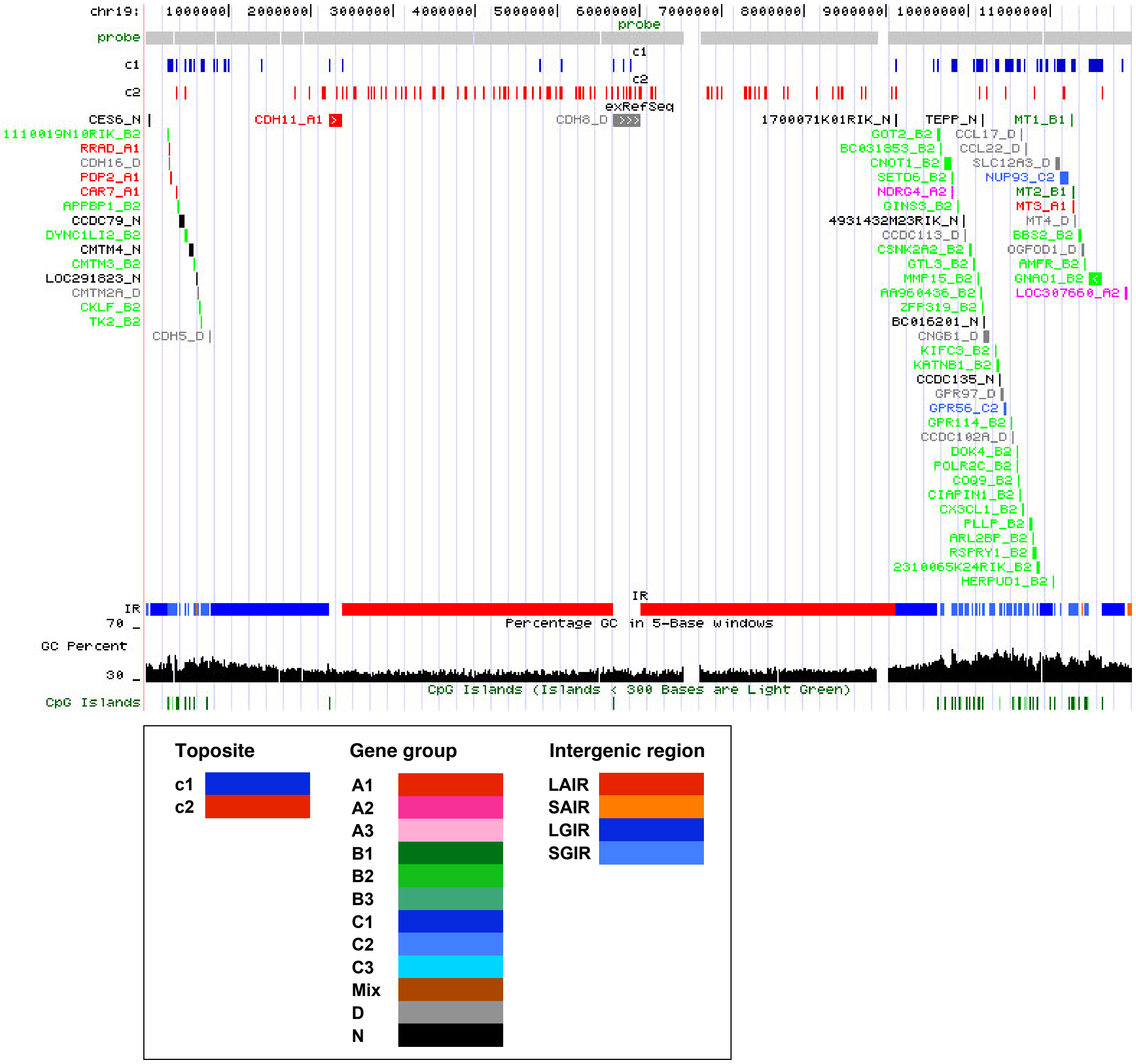

Figure S9. Overview of the topography of toposites and genes in the seven chromosomal regions analyzed by tiling arrays. Positional data for exRefSeq genes and toposites were uploaded and displayed in the custom track of UCSC genome browser. Gray bars on the top row (labeled "probe") depict the position of array probes. Areas without assigned probes or sequence gaps are blank. Classified toposites (c1, c2) are shown below. The exRefSeq genes, labeled by gene names and the expression group assignments, are discriminated by color codes shown on the bottom of the figure. Toposites and intergenic regions (IR) are also color-coded.

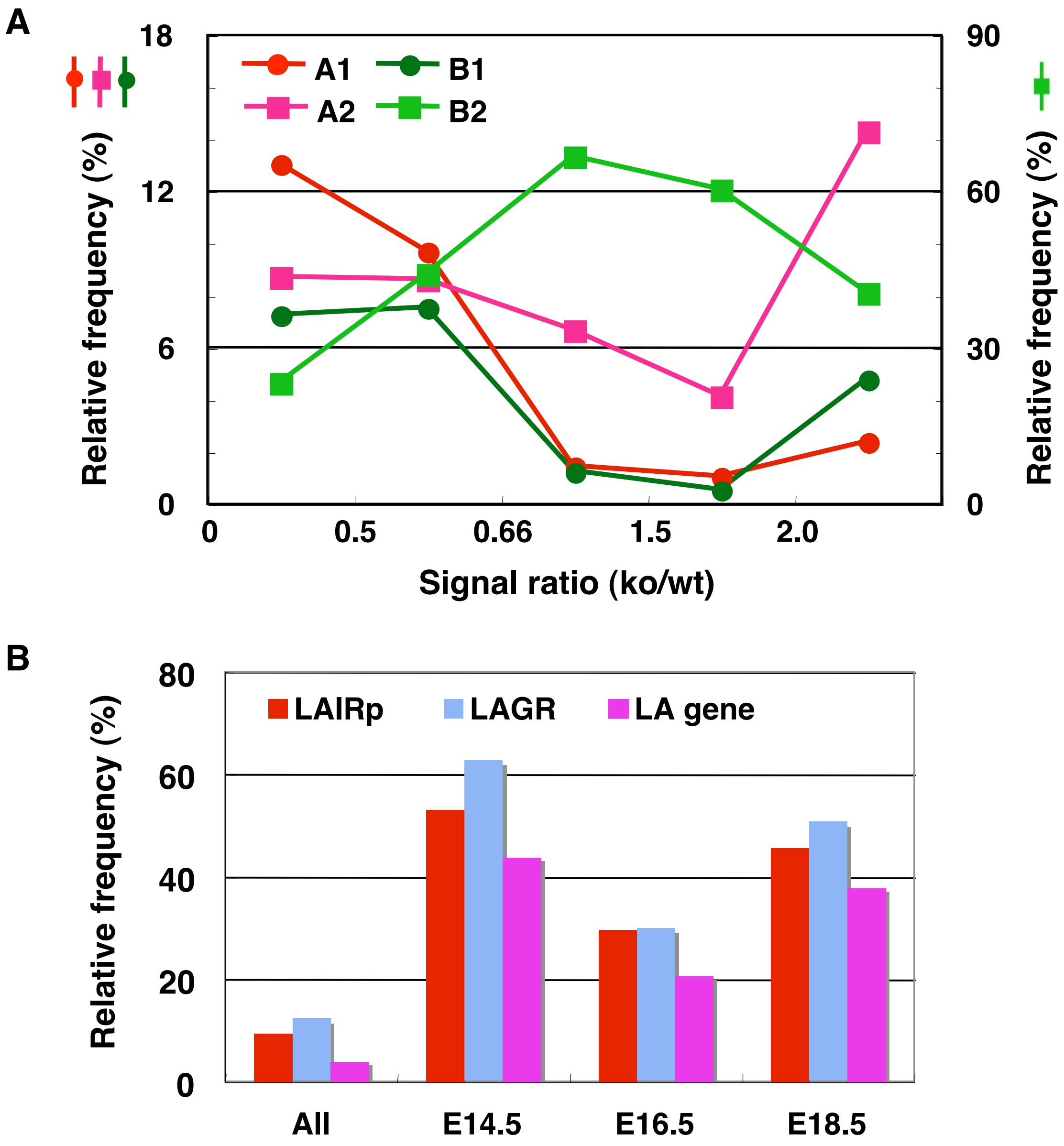

Figure S10. Similarity of expression patterns and genomic locations of relevant gene groups between embryonic brain and cultured granule cells. Our results were compared to the effects of topo IIβ depletion on the gene expression in mouse embryonic brain [7]. Their array data were obtained from GEO (Accession: GDS2702) or from the supplementary tables. **A**, The 12,433 probes on the array (Affymetrix GeneChip MG-U74A) were converted to RefSeq using g:Convert at the g:Profiler website (http://biit.cs.ut.ee/gprofiler/). Corresponding items (10,410) were then extracted from rat exRefSeq, low-signal probes ($P > 0.05$) were removed, and the remaining probes (6,299) were used for the analysis. Data sets for three embryonic days (E14.5, E16.5, E18.5) were averaged in wild type (wt) and the knockout mice ($top2b^{\Delta 2/\Delta 2}$), respectively, and the ratio (average signal intensity)^{ko}/(average signal intensity)^{wt} was calculated. In here, the ratio is sorted into 5 levels and the percentage of gene groups in each level were plotted. The results imply that the genes down-regulated by ICRF-193 in the post-mitotic granule cells (A1 and B1) are also down-regulated in the topo IIβ-depleted embryonic brain, whereas constitutively expressed genes (B2) are more or less unaffected by the gene knockout. **B**, With respect to three embryonic days (E14.5, E16.5, E18.5), probes differentially expressed in the topo IIβ-knockout (≥ 1.7-fold, $P \leq 0.01$) were extracted from the supplementary tables (Table S2-S4 of Ref. 7). Down-regulated probes were then selected, converted to rat exRefSeq, and relative frequencies of LAIR-proximal (LAIRp), LAGR, and LA genes were plotted in the graph. As a reference, those in all arrayed genes (All) are also shown. The results indicate that an extremely large proportion of down-regulated genes belong to LAIR-proximal, LAGR, and LA gene categories.

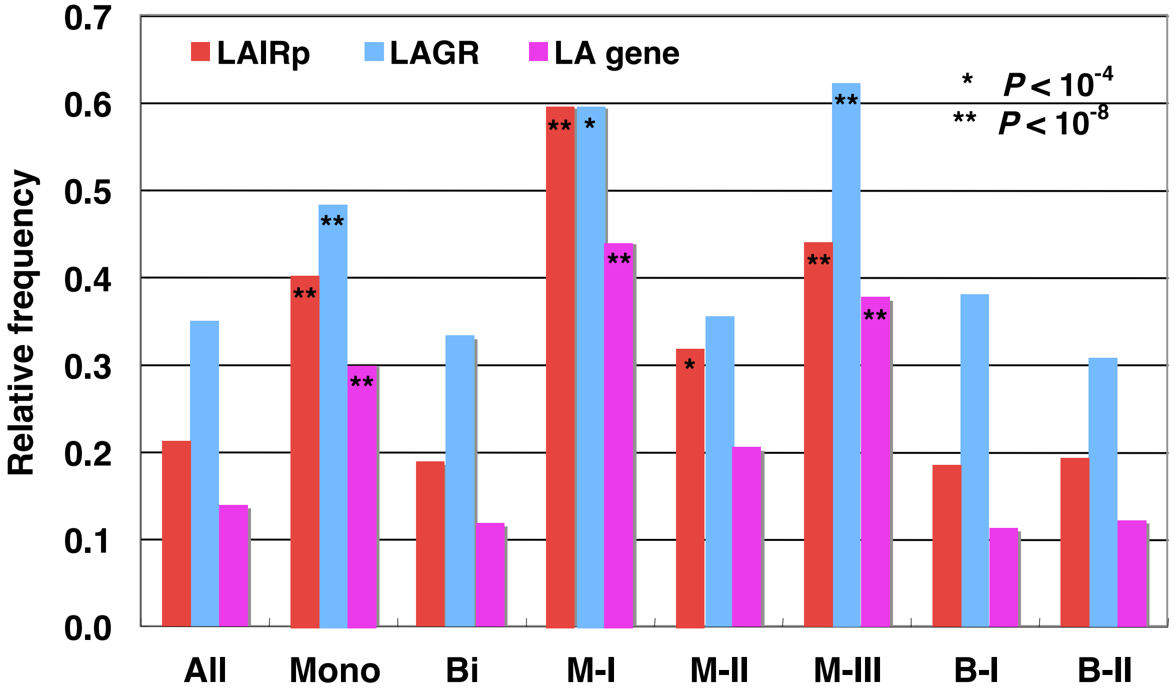

Figure S11. A high incidence of LA genes in monoallelically expressed autosomal genes. Considerably large proportion (5-10%) of human autosomal genes display random monoallelic transcription genome-wide [25]. Functional similarity of monoallelic genes and LA genes made us examine whether these genes also occupy similar positions in the genome. All the genes analyzed in the report (565 monoallelic and 4,407 biallelic) were uploaded to g:Orth at the g:Profiler website to sift out rat homologues. Corresponding items (505 monoallelic and 4,028 biallelic) were then extracted from rat exRefSeq and sorted into LAIR-proximal (LAIRp), LAGR, and LA gene. Monoallelic (Mono) and biallelic (Bi) genes had been classified into three (M-I, M-II, M-III) and two (B-I, B-II) classes, respectively, in the order of decreasing reliability (see the original report for details). The frequency data were plotted in the graph and statistical significance was calculated by the chi-square test against "All". The results indicate, with high statistical confidence, that a large proportion of monoallelic but not biallelic genes are LA genes.

Table S1. Genomic location of eTIP DNA clones

P1

Clone_number	eTIP_fraction	Chr	Start_clone	End_clone	Length_clone	GC_clone	Start_genome	End_genome	Length_genome	GC_genome	Type_genome	Unknown
1	P1	1	11,140,121	11,140,270	150	0.347	9,627,258	12,721,553	3,094,296	0.397	LAIR	
2	P1	1	46,342,347	46,342,589	243	0.481	45,129,729	46,364,322	1,234,594	0.431	LGIR	
3	P1	1	83,967,383	83,967,817	435	0.566	83,951,222	83,974,231	23,010	0.505	SGGR	
4	P1	1	106,662,967	106,663,512	546	0.344	106,461,235	107,249,830	788,596	0.415	LAIR	
5	P1	1	116,705,408	116,705,714	307	0.619	116,674,546	116,751,277	76,732	0.391	SAIR	
6	P1	1	120,161,549	120,161,653	105	0.324	120,129,217	120,195,434	66,218	0.452	LGGR	
7	P1	1	123,256,591	123,257,375	785	0.462	123,195,570	123,599,916	404,347	0.454	LGIR	
8	P1	1	134,329,628	134,329,886	259	0.463	134,003,873	134,380,697	376,825	0.436	LAGR	
9	P1	1	176,847,878	176,848,135	258	0.682	176,841,408	176,848,184	6,777	0.510	SGGR	
10	P1	1	184,766,742	184,767,315	574	0.467	184,762,784	184,789,365	26,582	0.526	SGGR	
11	P1	1	185,279,904	185,280,421	518	0.490	185,085,592	185,329,616	244,025	0.500	LGIR	
12	P1	1	206,002,897	206,002,971	75	0.547	205,975,267	206,006,198	30,932	0.494	SGIR	
13	P1	1	210,028,981	210,029,064	84	0.643	209,889,061	210,029,821	140,761	0.537	LGGR	
14	P1	1	234,190,504	234,191,277	774	0.481	234,147,881	234,556,764	408,884	0.430	LGIR	
15	P1	1	239,179,775	239,180,055	281	0.367	239,113,239	240,308,576	1,195,338	0.398	LAIR	
16	P1	1	244,818,012	244,818,521	510	0.402	243,799,047	244,827,001	1,027,955	0.405	LAGR	
17	P1	1	245,942,631	245,942,999	370	0.526	245,894,220	246,004,349	110,130	0.469	LGGR	
18	P1	2	18,956,132	18,956,888	757	0.452	18,674,539	19,269,425	594,887	0.385	LAGR	
19	P1	2	35,590,205	35,590,663	459	0.451	35,487,578	35,745,919	258,342	0.413	LAGR	
20	P1	2	36,841,617	36,841,847	231	0.420	36,591,963	37,986,805	1,394,843	0.416	LAIR	
21	P1	2	39,635,159	39,635,356	198	0.424	39,620,558	41,376,421	1,755,864	0.407	LAIR	
22	P1	2	46,438,522	46,438,876	355	0.456	46,407,853	46,469,312	61,460	0.419	SAIR	
23	P1	2	50,985,923	50,986,259	337	0.487	50,871,339	51,423,434	552,096	0.393	LAIR	
24	P1	2	66,304,562	66,305,324	763	0.328	62,631,406	66,379,904	3,748,499	0.372	LAIR	
25	P1	2	82,000,492	82,001,165	674	0.455	80,336,402	82,182,634	1,846,233	0.392	LAIR	
26	P1	2	94,983,307	94,983,885	579	0.463	94,870,286	95,118,885	248,600	0.466	LGIR	
27	P1	2	99,442,016	99,443,578	1,563	0.393	98,679,864	100,312,864	1,633,001	0.391	LAIR	
28	P1	2	120,704,931	120,705,321	391	0.463	120,338,197	120,913,932	575,736	0.437	LGIR	
29	P1	2	135,723,209	135,723,439	231	0.494	132,901,269	138,293,442	5,392,174	0.385	LAIR	
30	P1	2	145,395,829	145,396,289	461	0.351	145,355,305	145,684,649	329,345	0.404	LAIR	
31	P1	2	175,924,263	175,925,254	467	0.485	175,858,985	175,924,815	65,831	0.442	LGGR	
32	P1	2	191,445,729	191,446,345	617	0.501	191,435,731	191,455,586	19,856	0.481	SGGR	
33	P1	2	200,680,271	200,681,209	939	0.472	200,672,172	200,889,321	217,150	0.471	LGGR	
34	P1	2	206,510,991	206,511,731	741	0.372	205,877,692	209,288,190	3,410,499	0.378	LAIR	
35	P1	2	223,384,319	223,384,883	564	0.405	223,034,992	223,560,587	525,596	0.398	LAIR	
36	P1	3	3,615,830	3,616,290	461	0.551	3,615,328	3,622,874	7,547	0.511	SGIR	
37	P1	3	3,675,427	3,675,894	468	0.613	3,673,355	3,677,878	4,524	0.585	SGGR	
38	P1	3	115,925,191	115,925,485	295	0.539	115,632,509	116,098,566	466,058	0.468	LGIR	
39	P1	3	151,951,778	151,952,168	391	0.547	151,830,772	152,286,807	456,036	0.438	LGIR	
40	P1	3	168,040,705	168,041,536	832	0.488	167,495,691	168,737,916	1,242,226	0.488	LGIR	
41	P1	4	2,397,392	2,397,844	453	0.536	2,345,211	2,410,072	64,862	0.473	SGIR	
42	P1	4	35,116,219	35,117,507	1,289	0.363	35,077,383	37,244,676	2,167,294	0.382	LAIR	
43	P1	4	59,780,334	59,780,367	34	0.471	59,530,449	59,968,678	438,230	0.454	LGGR	
44	P1	4	117,508,659	117,509,118	460	0.548	117,490,463	117,513,122	22,660	0.510	SGGR	
45	P1	4	119,408,778	119,409,136	359	0.365	119,036,041	119,484,497	448,457	0.406	LAGR	
46	P1	4	150,827,252	150,827,628	377	0.536	150,679,206	151,031,291	352,086	0.507	LGIR	
47	P1	4	157,614,580	157,614,979	400	0.478	157,563,323	157,687,868	124,546	0.505	LGIR	
48	P1	4	160,175,427	160,176,186	760	0.447	160,152,043	160,203,647	51,605	0.392	SAIR	
49	P1	4	173,321,168	173,321,540	373	0.402	172,965,070	173,430,098	465,029	0.443	LGGR	
50	P1	4	179,871,274	179,871,569	296	0.463	179,841,303	179,933,313	92,011	0.423	SGIR	
51	P1	4	185,431,956	185,432,228	273	0.451	185,348,398	185,503,894	155,497	0.462	LGGR	
52	P1	5	1,962,128	1,962,813	686	0.423	1,960,602	1,975,728	15,127	0.398	SAGR	
53	P1	5	43,310,768	43,311,050	283	0.304	41,596,816	44,728,130	3,131,315	0.387	LAIR	
54	P1	5	80,301,394	80,301,900	507	0.580	80,202,981	80,323,140	120,160	0.516	LGGR	
55	P1	5	112,673,248	112,673,582	335	0.406	112,242,647	113,200,449	957,803	0.371	LAIR	
56	P1	5	147,448,621	147,448,933	313	0.460	147,431,026	147,803,312	372,287	0.489	LGGR	
57	P1	5	156,453,035	156,453,582	548	0.562	156,452,515	156,499,633	47,119	0.552	SGGR	
58	P1	5	165,253,262	165,253,832	571	0.541	165,249,268	165,254,697	5,430	0.568	SGGR	
59	P1	6	16,312,401	16,312,457	57	0.439	15,358,479	18,047,448	2,688,970	0.385	LAIR	
60	P1	6	17,876,320	17,876,980	661	0.386	15,358,479	18,047,448	2,688,970	0.385	LAIR	
61	P1	6	49,669,946	49,670,570	625	0.493	49,527,306	50,203,632	676,327	0.447	LGGR	
62	P1	6	95,104,055	95,104,642	588	0.471	95,068,717	95,165,317	96,601	0.433	SGIR	
63	P1	6	103,408,675	103,408,793	119	0.546	103,366,169	103,455,157	88,989	0.434	LAGR	
64	P1	6	110,164,828	110,165,289	462	0.526	109,949,267	110,173,834	224,568	0.443	LGGR	
65	P1	6	130,732,627	130,733,317	691	0.508	130,254,869	132,294,664	2,039,796	0.469	LGIR	
66	P1	6	146,236,475	146,237,223	749	0.382	146,196,114	146,267,078	70,965	0.417	LAGR	
67	P1	7	16,762,744	16,763,074	331	0.369	16,551,104	18,168,741	1,617,638	0.413	LAIR	
68	P1	7	99,175,388	99,175,824	437	0.467	98,992,288	101,142,530	2,150,243	0.410	LAIR	
69	P1	7	111,853,531	111,853,907	378	0.528	111,578,865	112,656,236	1,077,372	0.498	LGIR	
70	P1	7	127,437,685	127,438,221	537	0.594	127,437,652	127,457,504	19,853	0.494	SGGR	
71	P1	8	46,356,261	46,356,948	688	0.551	46,346,151	46,748,259	402,109	0.486	LGIR	
72	P1	8	52,725,720	52,726,096	377	0.599	52,660,214	52,726,803	66,590	0.468	LGGR	
73	P1	9	1,173,201	1,174,192	989	0.569	1,168,389	1,470,897	302,509	0.461	LGIR	
74	P1	9	27,709,786	27,710,204	419	0.599	24,496,855	29,466,087	4,969,233	0.380	LAIR	
75	P1	9	48,168,702	48,169,593	892	0.360	47,823,072	52,169,481	4,346,410	0.378	LAIR	
76	P1	9	65,077,317	65,077,641	325	0.345	64,828,755	65,144,324	315,570	0.403	LAIR	
77	P1	9	90,364,008	90,364,417	410	0.520	90,359,703	90,364,363	4,661	0.508	SGIR	
78	P1	9	90,877,115	90,877,317	203	0.517	90,850,109	90,892,349	42,241	0.511	SGGR	
79	P1	9	107,288,153	107,289,011	859	0.477	107,204,234	107,371,916	167,683	0.450	LGGR	
80	P1	10	37,165,258	37,165,605	348	0.526	37,160,268	37,168,183	7,916	0.574	SGGR	
81	P1	10	53,461,970	53,462,399	430	0.419	53,204,595	53,631,484	426,890	0.423	LGIR	
82	P1	10	55,929,337	55,929,765	429	0.385	55,922,264	55,930,947	8,684	0.482	SGIR	
83	P1	10	74,249,568	74,249,907	340	0.509	74,179,740	74,324,741	145,002	0.441	LGIR	
84	P1	10	80,098,303	80,098,773	471	0.406	80,011,205	81,746,197	1,734,993	0.388	LAIR	
85	P1	10	100,995,281	100,996,198	918	0.404	100,594,418	102,401,840	1,807,423	0.421	LGIR	
86	P1	11	6,392,767	6,393,116	350	0.409	5,436,557	7,463,398	2,026,842	0.369	LAIR	
87	P1	11	22,188,237	22,188,853	617	0.392	20,911,423	24,310,703	3,399,281	0.380	LAIR	
88	P1	11	25,751,181	25,752,308	1,128	0.381	25,549,749	27,125,338	1,575,590	0.389	LAIR	
89	P1	11	31,909,936	31,910,334	399	0.549	31,760,231	31,948,618	188,388	0.469	LGGR	
90	P1	11	53,106,531	53,106,694	164	0.293	53,093,890	53,122,851	28,962	0.364	SAGR	
91	P1	11	63,790,433	63,790,806	375	0.414	63,786,641	63,824,946	38,306	0.448	SGGR	
92	P1	11	71,151,160	71,151,227	68	0.485	70,987,964	71,188,149	200,186	0.471	LGIR	
93	P1	11	79,321,176	79,321,598	423	0.482	79,183,807	79,589,974	406,168	0.429	LGIR	
94	P1	12	15,459,250	15,459,683	434	0.532	15,436,324	15,606,411	170,088	0.515	LGIR	
95	P1	12	21,619,532	21,619,911	380	0.511	21,605,995	21,626,080	20,086	0.514	SGGR	
96	P1	13	5,201,050	5,201,307	258	0.295	2,293,552	7,714,512	5,420,961	0.388	LAIR	
97	P1	14	63,585,685	63,586,137	453	0.360	63,565,633	63,603,594	37,962	0.358	SAGR	
98	P1	14	69,263,930	69,264,307	378	0.394	67,893,944	70,332,839	2,438,896	0.391	LAIR	
99	P1	14	74,774,575	74,774,949	375	0.397	74,483,863	76,412,243	1,928,381	0.392	LAIR	
100	P1	14	92,241,076	92,241,677	602	0.374	90,246,567	92,255,920	2,009,354	0.426	LGIR	
101	P1	15	25,410,596	25,410,988	393	0.443	25,303,258	25,696,016	392,759	0.415	LAIR	
102	P1	15	26,641,077	26,641,172	96	0.354	26,625,852	26,654,204	28,353	0.400	SAIR	
103	P1	15	65,790,235	65,790,359	125	0.448	64,178,532	66,407,040	2,228,509	0.373	LAIR	
104	P1	15	70,259,655	70,259,753	99	0.404	69,755,348	70,777,020	1,021,673	0.401	LAIR	
105	P1	15	87,023,292	87,023,632	341	0.399	87,003,789	87,095,722	91,934	0.442	SGIR	
106	P1	16	4,214,633	4,214,734	102	0.451	4,173,110	4,856,843	683,734	0.440	LAGR	
107	P1	16	45,337,454	45,337,663	210	0.519	41,395,540	46,648,845	5,253,306	0.379	LAIR	
108	P1	16	51,956,834	51,957,205	372	0.406	51,878,723	52,149,282	270,560	0.434	LGIR	
109	P1	17	5,994,694	5,994,854	161	0.335	5,935,909	6,221,256	285,348	0.425	LGIR	
110	P1	17	42,708,178	42,708,606	429	0.497	42,180,429	42,987,773	807,345	0.414	LAIR	
111	P1	17	51,174,664	51,174,817	154	0.448	51,097,883	51,194,505	96,623	0.424	SGIR	
112	P1	18	29,725,705	29,725,932	228	0.566	29,688,206	29,954,677	266,472	0.405	LAGR	
113	P1	18	34,798,157	34,798,529	373	0.397	33,297,640	35,095,405	1,797,766	0.397	LAIR	
114	P1	18	46,353,794	46,354,346	553	0.373	45,780,542	46,359,851	579,310	0.388	LAIR	
115	P1	18	54,521,414	54,522,088	675	0.456	54,476,267	54,535,657	59,391	0.412	SAIR	
116	P1	18	72,551,186	72,551,399	214	0.556	72,435,958	72,714,371	278,414	0.500	LGGR	
117	P1	18	78,545,566	78,546,370	805	0.460	77,943,623	78,932,010	988,388	0.456	LGIR	
118	P1	19	23,021,782	23,022,157	376	0.426	23,016,221	23,072,979	56,759	0.448	SGIR	
119	P1	19	27,911,139	27,911,224	86	0.581	27,727,068	28,484,803	757,736	0.409	LAGR	
120	P1	19	35,809,407	35,809,984	578	0.516	35,790,051	35,811,921	21,871	0.517	SGGR	
121	P1	19	44,604,940	44,605,314	375	0.387	44,574,533	45,862,608	1,288,076	0.456	LGIR	
122	P1	19	52,522,159	52,522,545	387	0.517	52,273,647	52,531,571	257,925	0.532	LGIR	
123	P1	19	53,221,727	53,222,052	326	0.589	53,213,500	53,387,651	174,152	0.460	LGGR	
124	P1	20	7,116,355	7,116,689	335	0.645	7,086,073	7,123,750	37,678	0.520	SGGR	
125	P1	20	28,056,863	28,057,138	276	0.525	28,055,736	28,094,032	38,297	0.511	SGGR	
126	P1	20	40,014,319	40,014,711	393	0.318	38,934,573	40,734,781	1,800,209	0.381	LAIR	
127	P1	20	52,564,750	52,565,390	641	0.437	50,001,761	53,258,695	3,256,935	0.396	LAIR	
128	P1	20	54,698,179	54,698,691	513	0.472	54,629,605	54,897,875	268,271	0.379	LAGR	
129	P1	X	24,595,624	24,596,236	613	0.413	24,512,914	24,661,805	148,892	0.396	LAIR	
130	P1	X	40,750,166	40,750,390	225	0.382	40,719,992	40,877,881	157,890	0.402	LAGR	
131	P1	X	65,164,790	65,165,150	361	0.404	65,156,808	65,181,274	24,467	0.350	SAGR	
132	P1	X	69,739,206	69,739,452	247	0.364	69,448,959	71,746,813	2,297,855	0.369	LAGR	
133	P1	X	103,308,996	103,309,490	495	0.463	103,292,293	103,391,023	98,731	0.359	LAGR	
134	P1	X	152,378,216	152,378,572	357	0.457	150,134,410	152,741,777	2,607,368	0.379	LAIR	
135	P1	X	157,400,647	157,401,084	438	0.457	157,319,431	157,632,371	312,941	0.412	LAIR	
136	P1	4_random	1,292,601	1,293,037	437	0.501						chrUn
137	P1	Un	38,640,149	38,640,775	627	0.488						chrUn
138	P1	Un	63,528,006	63,528,642	887	0.437						chrUn
139	P1	Un_random	3,302,144	3,302,763	768	0.466						chrUn
140	P1	NA	NA	NA	310	0.477						LINE
141	P1	NA	NA	NA	393	0.374						LINE
142	P1	NA	NA	NA	446	0.390						LINE
143	P1	NA	NA	NA	406	0.436						LINE
144	P1	NA	NA	NA	402	0.438						LINE
145	P1	NA	NA	NA	141	0.383						LINE
146	P1	NA	NA	NA	167	0.359						LINE
147	P1	NA	NA	NA	302	0.533						LINE
148	P1	NA	NA	NA	373	0.397						LINE
149	P1	NA	NA	NA	483	0.546						LINE
150	P1	NA	NA	NA	796	0.449						LINE
151	P1	NA	NA	NA	313	0.530						LINE
152	P1	NA	NA	NA	240	0.546						LINE
153	P1	NA	NA	NA	149	0.403						LINE
154	P1	NA	NA	NA	360	0.503						LINE
155	P1	NA	NA	NA	45	0.600						LINE
156	P1	NA	NA	NA	282	0.422						LINE
157	P1	NA	NA	NA	335	0.359						satellitel
158	P1	NA	NA	NA	161	0.348						satellitel
159	P1	NA	NA	NA	295	0.342						satellitel
160	P1	NA	NA	NA	104	0.385						satellitel
161	P1	Un	38,447,233	38,448,267	1,035	0.348						satellitel
162	P1	NA	NA	NA	34	0.500						Simple_repeat
163	P1	NA	NA	NA	35	0.486						Simple_repeat

Column label	Description
Clone_number	serial number of eTIP clones
eTIP_fraction	eTIP DNA fraction used for cloning
Chr	chromosome number for the eTIP clone location
Start_clone	genomic position of the clone start
End_clone	genomic position of the clone end
Length_clone	sequence length of the clone
GC_clone	GC content of the clone
Start_genome	start of subgenomic region containing the cloned site
End_genome	end of subgenomic region containing the cloned site
Length_genome	length of subgenomic region containing the cloned site
GC_genome	GC content of subgenomic region containing the cloned site
Type_genome	type of subgenomic region containing the cloned site
Unknown	clones unable to locate (repeats and unknown genomic location)

NA = not applicable

P2

Clone_number	eTIP_fraction	Chr	Start_clone	End_clone	Length_clone	GC_clone	Start_genome	End_genome	Length_genome	GC_genome	Type_genome	Unknown
164	P2	1	12,581,609	12,581,641	56	0.394	9,627,258	12,721,553	3,094,296	0.397	LAIR	
165	P2	1	18,746,980	18,747,233	254	0.378	18,203,383	18,885,521	682,139	0.394	LAGR	
166	P2	1	63,271,714	63,272,197	484	0.312	63,254,542	63,294,304	39,763	0.385	SAIR	
167	P2	1	70,614,216	70,614,359	144	0.431	70,552,957	70,732,772	179,816	0.377	LAIR	
168	P2	1	70,762,466	70,763,143	678	0.344	70,758,622	71,174,171	415,550	0.375	LAIR	
169	P2	1	93,098,700	93,099,385	686	0.379	92,692,488	93,408,682	716,195	0.420	LAIR	
170	P2	1	93,309,740	93,310,112	373	0.397	92,692,488	93,408,682	716,195	0.420	LAIR	
171	P2	1	105,682,257	105,682,673	417	0.341	101,876,542	106,039,069	4,162,528	0.386	LAIR	
172	P2	1	107,258,953	107,258,972	20	0.450	107,249,831	107,285,248	35,418	0.396	SAGR	
173	P2	1	112,686,119	112,686,333	215	0.479	111,819,879	116,672,886	4,853,008	0.379	LAIR	
174	P2	1	141,072,563	141,072,985	423	0.381	141,038,893	141,230,359	191,467	0.436	LGIR	
175	P2	1	141,968,446	141,968,556	325	0.387	141,940,212	141,992,737	52,526	0.377	LAGR	
176	P2	1	182,831,211	182,832,224	1,014	0.426	182,506,461	184,660,053	2,153,593	0.439	LGIR	
177	P2	1	214,779,344	214,779,987	644	0.363	214,756,742	214,785,598	28,857	0.404	SAIR	
178	P2	1	216,921,674	216,921,772	100	0.535	216,852,785	216,928,790	76,006	0.384	SAIR	
179	P2	1	252,210,139	252,210,253	115	0.487	252,164,828	252,221,749	56,922	0.495	LGGR	
180	P2	2	16,717,698	16,717,911	214	0.439	15,176,414	18,674,538	3,498,125	0.395	LAIR	
181	P2	2	91,201,749	91,202,094	346	0.347	89,812,388	92,111,971	2,299,584	0.392	LAIR	
182	P2	2	99,422,324	99,422,725	402	0.405	98,679,864	100,312,864	1,633,001	0.391	LAIR	
183	P2	2	108,272,501	108,272,951	451	0.297	107,633,835	111,132,906	3,499,072	0.388	LAIR	
184	P2	2	108,904,225	108,904,363	139	0.367	107,633,835	111,132,906	3,499,072	0.388	LAIR	
185	P2	2	132,548,688	132,549,386	699	0.330	131,456,048	132,839,236	1,383,189	0.387	LAIR	
186	P2	2	134,598,468	134,598,839	372	0.409	132,901,269	138,293,442	5,392,174	0.385	LAIR	
187	P2	2	145,253,683	145,254,003	321	0.424	144,789,735	145,355,304	565,570	0.392	LAGR	
188	P2	2	164,233,456	164,234,020	565	0.350	163,661,939	164,279,415	617,477	0.369	LAIR	
189	P2	2	166,145,015	166,145,581	567	0.347	166,091,511	166,309,373	217,863	0.386	LAGR	
190	P2	2	166,915,426	166,915,735	310	0.284	166,807,229	166,938,118	130,890	0.395	LAIR	
191	P2	2	185,290,873	185,291,164	292	0.401	185,105,648	185,326,557	220,910	0.379	LAGR	
192	P2	2	207,796,624	207,796,875	252	0.421	205,877,692	209,288,190	3,410,499	0.378	LAIR	
193	P2	2	215,276,642	215,276,854	213	0.333	214,894,918	215,781,871	886,954	0.387	LAGR	
194	P2	2	219,809,154	219,809,281	207	0.484	219,692,238	219,951,013	258,776	0.432	LGIR	
195	P2	2	238,160,918	238,161,654	737	0.342	237,327,480	238,969,798	1,642,319	0.386	LAIR	
196	P2	2	248,496,369	248,496,439	71	0.465	247,253,288	249,779,206	2,525,919	0.399	LAIR	
197	P2	2	256,656,022	256,656,338	317	0.347	256,242,265	256,760,567	518,303	0.396	LAGR	
198	P2	2	257,456,571	257,456,966	395	0.366	256,760,568	257,777,856	1,017,289	0.385	LAIR	
199	P2	3	61,068,409	61,068,806	398	0.382	60,374,723	61,450,744	1,076,022	0.404	LAIR	
200	P2	3	67,849,320	67,849,354		0.686	67,843,944	67,865,179	21,236	0.470	SGIR	
201	P2	3	81,570,301	81,570,370	217	0.371	78,741,226	81,707,710	2,966,485	0.394	LAIR	
202	P2	3	81,610,981	81,611,272	292	0.370	78,741,226	81,707,710	2,966,485	0.394	LAIR	
203	P2	3	82,841,222	82,841,411	190	0.274	82,310,096	86,661,217	4,351,122	0.381	LAIR	
204	P2	3	94,097,725	94,097,948	584	0.348	92,681,850	94,534,306	1,852,457	0.380	LAIR	
205	P2	3	97,632,956	97,633,342	387	0.336	97,631,845	97,726,396	94,552	0.429	SGIR	
206	P2	3	110,113,325	110,113,611	287	0.484	109,623,786	111,795,502	2,171,717	0.408	LAIR	
207	P2	3	122,514,253	122,514,565	313	0.288	122,497,124	122,540,486	43,363	0.386	SAGR	
208	P2	3	129,140,163	129,141,010	848	0.400	128,480,746	130,601,607	2,120,862	0.400	LAGR	
209	P2	4	11,404,609	11,404,696	120	0.364	10,704,307	12,465,088	1,760,782	0.390	LAIR	
210	P2	4	12,548,865	12,548,925	61	0.426	12,495,729	12,785,189	289,461	0.402	LAIR	
211	P2	4	17,771,850	17,772,063	214	0.449	17,572,123	17,790,701	218,579	0.367	LAGR	
212	P2	4	46,512,288	46,512,596	311	0.511	44,953,399	47,771,964	2,818,566	0.401	LAIR	
213	P2	4	53,455,653	53,455,799	217	0.408	52,523,352	54,207,332	1,683,981	0.387	LAIR	
214	P2	4	86,410,854	86,411,239	386	0.355	86,360,844	86,411,751	50,908	0.397	SAIR	
215	P2	4	100,806,926	100,807,749	824	0.383	98,049,362	104,179,441	6,130,080	0.404	LAIR	
216	P2	4	105,877,454	105,877,928	475	0.419	105,868,489	105,894,762	26,274	0.448	SGGR	
217	P2	4	109,277,625	109,277,883	259	0.463	108,134,835	111,020,804	2,885,970	0.385	LAIR	
218	P2	4	111,913,233	111,913,514	282	0.507	111,020,805	112,166,900	1,146,096	0.394	LAGR	
219	P2	4	146,649,307	146,649,747	441	0.336	146,577,419	147,515,951	938,533	0.391	LAGR	
220	P2	4	169,759,602	169,760,311	710	0.310	169,642,903	169,800,449	157,547	0.390	LAIR	
221	P2	5	9,154,132	9,154,212	81	0.432	9,137,084	9,172,186	35,103	0.356	SAGR	
222	P2	5	9,512,719	9,512,845	127	0.299	9,306,082	9,561,278	255,197	0.403	LAIR	
223	P2	5	40,316,574	40,316,877	304	0.339	40,307,897	40,340,207	32,311	0.379	SAGR	
224	P2	5	42,154,088	42,154,428	341	0.393	41,596,816	44,728,130	3,131,315	0.387	LAIR	
225	P2	5	71,566,286	71,566,626	341	0.396	71,316,652	71,764,431	447,780	0.423	LGIR	
226	P2	6	14,980,663	14,980,844	182	0.412	14,048,116	15,358,478	1,310,363	0.402	LAGR	
227	P2	6	16,674,677	16,675,129	453	0.530	15,358,479	18,047,448	2,688,970	0.385	LAIR	
228	P2	6	19,474,550	19,474,737	189	0.410	18,047,894	19,790,014	1,742,121	0.383	LAIR	
229	P2	6	58,206,039	58,206,244	206	0.413	57,766,838	59,213,075	1,446,238	0.390	LAIR	
230	P2	6	62,223,888	62,224,164	277	0.401	61,233,773	62,283,462	1,049,690	0.381	LAIR	
231	P2	6	68,672,548	68,672,854	307	0.414	66,323,274	69,219,841	2,896,568	0.377	LAIR	
232	P2	6	71,316,051	71,316,115	92	0.477	70,685,391	71,429,729	744,339	0.404	LAIR	
233	P2	6	86,348,798	86,349,023	227	0.460	86,318,640	86,383,762	65,123	0.375	LAGR	
234	P2	6	95,477,339	95,477,628	290	0.652	95,473,617	95,478,497	4,881	0.521	SGGR	
235	P2	6	118,998,680	118,998,861	182	0.286	115,476,462	119,573,131	4,096,670	0.383	LAIR	
236	P2	6	137,374,577	137,374,955	379	0.570	137,356,748	137,426,439	69,692	0.508	SGIR	
237	P2	6	141,603,421	141,603,870	450	0.347	140,581,587	142,910,248	2,328,662	0.383	LAIR	
238	P2	7	7,281,144	7,281,480	337	0.421	7,206,550	7,348,975	142,426	0.366	LAIR	
239	P2	7	14,831,416	14,831,447	32	0.344	14,452,376	15,551,963	1,099,588	0.384	LAIR	
240	P2	7	34,422,502	34,422,911	410	0.420	34,051,829	35,024,153	972,325	0.407	LAIR	
241	P2	7	34,536,679	34,536,992	314	0.404	34,051,829	35,024,153	972,325	0.407	LAIR	
242	P2	7	45,393,104	45,393,195	92	0.413	44,522,812	45,459,921	937,110	0.380	LAIR	
243	P2	7	53,168,766	53,168,939	174	0.362	51,899,728	53,612,617	1,712,890	0.380	LAIR	
244	P2	7	75,060,848	75,060,917	76	0.429	74,777,733	75,292,501	514,769	0.381	LAGR	
245	P2	7	75,992,794	75,992,885	92	0.478	75,550,155	76,231,210	681,056	0.406	LAIR	
246	P2	7	108,084,291	108,084,454	164	0.494	106,648,973	109,190,687	2,541,715	0.392	LAIR	
247	P2	7	111,690,458	111,690,663	206	0.369	111,578,865	112,656,236	1,077,372	0.498	LGIR	
248	P2	7	129,430,705	129,430,804	100	0.460	129,371,305	129,490,977	119,673	0.429	LAGR	
249	P2	8	17,646,694	17,646,875	182	0.357	17,542,213	17,687,843	145,631	0.377	LAIR	
250	P2	8	65,635,702	65,636,048	347	0.429	65,550,128	66,065,273	515,146	0.485	LGIR	
251	P2	8	73,673,602	73,673,979	378	0.492	73,071,254	73,836,648	765,395	0.457	LGGR	
252	P2	8	89,577,984	89,578,135	152	0.336	89,210,469	90,518,833	1,308,365	0.396	LAIR	
253	P2	9	4,325,814	4,326,034	221	0.367	4,112,650	4,652,000	539,351	0.400	LAGR	
254	P2	9	40,586,050	40,586,417	368	0.492	40,012,912	41,875,036	1,862,125	0.413	LAIR	
255	P2	9	40,775,721	40,776,250	530	0.400	40,012,912	41,875,036	1,862,125	0.413	LAIR	
256	P2	9	49,033,072	49,033,301	230	0.343	47,823,072	52,169,481	4,346,410	0.378	LAIR	
257	P2	9	76,255,839	76,256,158	320	0.369	74,995,931	76,860,584	1,864,654	0.422	LGIR	
258	P2	9	95,729,966	95,729,994	29	0.207	95,073,189	96,252,103	1,178,915	0.382	LAIR	
259	P2	9	95,940,258	95,940,507	250	0.356	95,073,189	96,252,103	1,178,915	0.382	LAIR	
260	P2	9	96,789,543	96,789,567	25	0.360	96,721,636	96,834,986	113,351	0.369	LAGR	
261	P2	9	97,312,207	97,312,461	255	0.455	97,301,058	97,313,664	12,607	0.424	SGIR	
262	P2	9	104,555,957	104,556,924	968	0.415	104,548,520	104,578,392	29,873	0.413	SAGR	
263	P2	10	2,600,275	2,600,355	207	0.395	2,177,760	3,577,373	1,399,614	0.437	LGIR	
264	P2	10	8,054,948	8,055,880	933	0.355	7,232,029	8,214,342	982,314	0.430	LGIR	
265	P2	10	52,602,864	52,603,235	372	0.395	52,554,151	52,904,206	350,056	0.441	LGIR	
266	P2	11	3,268,056	3,268,466	411	0.387	3,026,113	3,811,195	785,083	0.386	LAIR	
267	P2	11	12,348,479	12,348,743	268	0.392	11,722,381	12,662,855	940,475	0.397	LAIR	
268	P2	11	29,179,352	29,179,632	281	0.363	29,011,014	29,181,414	170,401	0.412	LAIR	
269	P2	11	39,114,346	39,114,567	228	0.374	38,359,827	40,608,858	2,249,032	0.383	LAIR	
270	P2	11	42,623,220	42,623,278	59	0.322	42,594,112	42,625,520	31,409	0.390	SAIR	
271	P2	11	49,590,708	49,591,287	580	0.438	49,480,414	49,689,285	208,872	0.391	LAGR	
272	P2	11	60,414,284	60,414,594	311	0.450	60,227,995	60,928,794	700,800	0.406	LAGR	
273	P2	11	62,643,394	62,643,647	254	0.469	60,928,795	63,559,183	2,630,389	0.392	LAIR	
274	P2	11	65,833,595	65,833,965	371	0.399	65,761,763	65,867,905	106,143	0.381	LAGR	
275	P2	13	1,693,772	1,694,057	286	0.336	1,190,787	2,293,551	1,102,765	0.391	LAGR	
276	P2	13	17,301,558	17,301,708	151	0.358	17,009,581	18,151,050	1,141,470	0.380	LAIR	
277	P2	13	18,239,293	18,239,593	301	0.352	18,233,199	19,395,318	1,162,120	0.378	LAIR	
278	P2	13	20,041,914	20,042,666	749	0.365	19,401,497	22,858,970	3,457,474	0.386	LAIR	
279	P2	13	23,698,274	23,698,476	203	0.350	22,858,971	23,916,252	1,057,282	0.385	LAGR	
280	P2	13	23,879,425	23,879,815	391	0.373	22,858,971	23,916,252	1,057,282	0.385	LAGR	
281	P2	13	42,374,869	42,375,356	488	0.283	42,207,526	43,132,635	925,110	0.404	LAGR	
282	P2	13	54,752,024	54,752,788	765	0.437	53,819,068	55,962,592	2,143,525	0.387	LAIR	
283	P2	13	54,988,441	54,988,627	187	0.353	53,819,068	55,962,592	2,143,525	0.387	LAIR	
284	P2	13	59,857,680	59,858,377	698	0.312	58,643,855	60,598,420	1,954,566	0.370	LAIR	
285	P2	13	59,903,255	59,903,695	441	0.293	58,643,855	60,598,420	1,954,566	0.370	LAIR	
286	P2	13	62,981,454	62,981,677	224	0.402	61,038,331	64,149,799	3,111,469	0.375	LAIR	
287	P2	13	76,328,229	76,328,658	430	0.365	75,664,145	76,360,873	696,729	0.413	LAGR	
288	P2	14	1,116,601	1,116,688	88	0.466	1,115,860	1,123,654	7,795	0.374	SAGR	
289	P2	14	58,085,135	58,085,301	167	0.359	57,327,280	61,634,236	4,306,957	0.381	LAIR	
290	P2	14	58,670,725	58,671,626	902	0.320	57,327,280	61,634,236	4,306,957	0.381	LAIR	
291	P2	14	65,043,209	65,043,618	410	0.361	64,377,768	65,470,390	1,092,623	0.386	LAIR	
292	P2	14	94,742,914	94,743,099	186	0.409	93,328,556	97,093,674	3,765,119	0.384	LAIR	
293	P2	14	108,906,538	108,906,622	85	0.353	107,471,200	109,221,990	1,750,791	0.388	LAIR	
294	P2	15	10,573,190	10,573,308	119	0.454	9,369,339	10,703,502	1,334,164	0.444	LGIR	
295	P2	15	30,817,060	30,817,374	315	0.381	30,651,211	31,774,922	1,123,712	0.465	LGIR	
296	P2	15	54,733,882	54,734,268	387	0.380	54,283,606	55,478,482	1,194,877	0.406	LAIR	
297	P2	15	66,140,830	66,141,233	404	0.408	64,178,532	66,407,040	2,228,509	0.373	LAIR	
298	P2	15	78,644,430	78,645,372	943	0.364	76,754,631	79,073,534	2,318,904	0.381	LAIR	
299	P2	15	95,586,502	95,586,869	368	0.307	95128682	96419592	1290911	0.368	LAIR	
300	P2	15	95,615,590	95,615,929	340	0.400	95128682	96419592	1290911	0.368	LAIR	
301	P2	15	97,145,458	97,145,830	373	0.391	96427546	99932678	3505133	0.384	LAIR	
302	P2	15	104,385,938	104,386,160	223	0.368	104173571	104930116	756546	0.423	LAGR	
303	P2	16	21,164,617	21,164,764	148	0.459	20265572	21907226	1641655	0.444	LGIR	
304	P2	16	27,942,724	27,942,968	245	0.453	26716331	28433103	1716773	0.430	LGIR	
305	P2	16	29,191,091	29,191,358	268	0.336	28643665	29337208	693544	0.388	LAIR	
306	P2	16	29,609,044	29,609,074	31	0.290	29337209	29751934	414726	0.389	LAGR	
307	P2	16	66,046,535	66,046,654	120	0.450	64959680	67102372	2142693	0.410	LAIR	
308	P2	16	89,589,196	89,589,238	43	0.395	86217019	89925334	3708316	0.390	LAIR	
309	P2	17	2,279,390	2,279,794	405	0.491	1396429	2559789	1163361	0.428	LGIR	
310	P2	17	43,693,344	43,693,514	171	0.339	43674618	43727703	53086	0.367	SAIR	
311	P2	17	48,091,105	48,091,171	67	0.448	47926578	48209431	282854	0.434	LAGR	
312	P2	17	53,187,560	53,187,908	349	0.344	53163918	53404917	241000	0.401	LAIR	
313	P2	17	58,214,868	58,215,514	647	0.360	57928563	58539655	611093	0.395	LAIR	
314	P2	17	81,595,290	81,595,706	417	0.398	80023658	82719186	2695529	0.410	LAIR	
315	P2	17	89,962,265	89,962,325	61	0.377	89161298	90583187	1421890	0.409	LAIR	
316	P2	18	11,506,409	11,506,604	196	0.342	8268463	11855312	3586850	0.391	LAIR	
317	P2	18	36,481,856	36,482,626	771	0.361	36379307	36537147	157841	0.399	LAIR	
318	P2	18	41,041,514	41,041,947	434	0.355	40981869	41054118	72250	0.382	LAGR	
319	P2	18	83,326,698	83,327,517	820	0.329	83180499	83380394	199896	0.387	LAIR	
320	P2	19	3,249,281	3,249,566	286	0.486	2381813	5690641	3308829	0.379	LAIR	
321	P2	19	33,868,240	33,868,278	120	0.333	32880251	34888985	2008735	0.399	LAIR	
322	P2	20	52,577,552	52,577,793	242	0.380	50001761	53258695	3256935	0.396	LAIR	
323	P2	X	26,311,696	26,311,988	293	0.382	26307058	26328589	21532	0.434	SGIR	
324	P2	X	31,813,306	31,813,524	219	0.374	31297945	33078597	1780653	0.389	LAIR	
325	P2	X	47,862,175	47,862,407	233	0.446	46478925	47870064	1391140	0.402	LAGR	
326	P2	X	58,033,741	58,033,890	150	0.393	56878683	58251535	1372853	0.405	LAIR	
327	P2	X	58,374,075	58,374,244	170	0.371	58251536	58495865	244330	0.354	LAGR	
328	P2	X	60,106,619	60,106,668	50	0.380	59224346	61133130	1908785	0.385	LAIR	
329	P2	X	66,669,789	66,669,972	184	0.375	65566059	67575764	2009706	0.398	LAIR	
330	P2	X	95,262,551	95,262,942	392	0.370	95253942	95266384	12443	0.363	SAGR	
331	P2	X	111,834,022	111,834,346	325	0.360	110631154	112317953	1686800	0.385	LAIR	
332	P2	X	112,077,014	112,077,633	620	0.371	110631154	112317953	1686800	0.385	LAIR	
333	P2	X	114,585,452	114,586,338	887	0.379	113711362	116715195	3003834	0.387	LAIR	
334	P2	X	116,814,618	116,814,667	50	0.440	116715196	117331820	616625	0.372	LAGR	
335	P2	X	124,093,799	124,094,213	415	0.357	123660716	124385499	724784	0.429	LGIR	
336	P2	X	124,465,884	124,466,464	581	0.460	124456247	124467339	11093	0.400	SAGR	
337	P2	X	144,414,889	144,414,921	92	0.455	144272966	144606918	333953	0.419	LAGR	
338	P2	X	145,001,879	145,002,036	158	0.614	144606919	145601395	994477	0.400	LAIR	
339	P2	X	154,058,568	154,058,891	324	0.361	152746273	154829391	2083119	0.389	LAIR	
340	P2	X	154,278,843	154,278,987	145	0.352	152746273	154829391	2083119	0.389	LAIR	
341	P2	Un	8,653,051	8,653,186	136	0.485						chrUn
342	P2	Un	28,565,093	28,565,476	384	0.645						chrUn
343	P2	NA	NA	NA	450	0.391						LINE
344	P2	NA	NA	NA	557	0.386						LINE
345	P2	NA	NA	NA	128	0.352						LINE
346	P2	NA	NA	NA	372	0.395						LINE
347	P2	NA	NA	NA	271	0.358						LINE
348	P2	NA	NA	NA	195	0.589						LINE
349	P2	NA	NA	NA	224	0.429						LINE
350	P2	NA	NA	NA	285	0.400						LINE
351	P2	NA	NA	NA	293	0.300						LINE
352	P2	NA	NA	NA	325	0.631						LINE
353	P2	NA	NA	NA	332	0.581						LINE
354	P2	NA	NA	NA	341	0.457						LINE
355	P2	NA	NA	NA	341	0.396						LINE
356	P2	NA	NA	NA	341	0.396						LINE
357	P2	NA	NA	NA	342	0.540						LINE
358	P2	NA	NA	NA	342	0.449						LINE
359	P2	NA	NA	NA	373	0.552						LINE
360	P2	NA	NA	NA	373	0.397						LINE
361	P2	NA	NA	NA	373	0.399						LINE
362	P2	NA	NA	NA	459	0.370						LINE
363	P2	NA	NA	NA	283	0.399						LINE
364	P2	NA	NA	NA	504	0.401						multi
365	P2	NA	NA	NA	75	0.307						satellitel
366	P2	NA	NA	NA	296	0.453						satellitel
367	P2	NA	NA	NA	473	0.531						satellitel
368	P2	NA	NA	NA	375	0.365						satellitel
369	P2	NA	NA	NA	276	0.391						satellitel
370	P2	NA	NA	NA	35	0.486						Simple_repeat
371	P2	NA	NA	NA	45	0.489						Simple_repeat
372	P2	NA	NA	NA	46	0.500						Simple_repeat
373	P2	NA	NA	NA	51	0.490						Simple_repeat
374	P2	NA	NA	NA	34	0.500						Simple_repeat
375	P2	NA	NA	NA	34	0.500						Simple_repeat
376	P2	NA	NA	NA	34	0.500						Simple_repeat
377	P2	NA	NA	NA	43	0.488						Simple_repeat
378	P2	NA	NA	NA	368	0.364						SINE&LINE
379	P2	NA	NA	NA	299	0.378						SINE&LINE

Table S2. eTIP PCR primers

Clone_number	Genomic_position			Primer_sequence_forward	Primer_sequence_reverse	Amplification_target
123	chr19	53221727	53222052	GGGTGTCTACCTTGGGCGA	AGCCTCAAGTTTTGATTCCCC	chr19:53221865-53222007
32	chr2	191445729	191446345	GAGAGGTGCCCGAGTCAATG	AGGAAGATGTGCCCGAAGAA	chr2:191445970-191446127
31	chr2	175924263	175925254	CCCCTCACTTGTTAATTTGATGC	TTTCTTTACCTCGCTGACCAGTC	chr2:175924271-175924465
51	chr4	185431956	185432228	CGACTGGCCCACACAGTTT	CCGCACTCCTATACTTTGCGA	chr4:185431991-185432164
60	chr6	17876320	17876980	AGCTGGTCTCGAGGACTGATG	TTGGTGCAACTGGGTTCTGTT	chr6:17876336-17876501
103	chr15	65790235	65790359	ATTGGATGCAGCAAGAGGTCA	TGAACAAGAGAACCCCAGGG	chr2:36841662-36841815
98	chr14	69263930	69264307	GCTTATGCAGGTACATGAGCTGC	CAGAACTACACGTACCAGGCTGTG	chr14:69264141-69264257
4	chr1	106662967	106663512	TTGACAATGCACCTGAAAGCTC	TCTCGGAGAAATGTTGATTCTTTG	chr1:106687302-106687444
254	chr9	40586050	40586417	ATCCTCCATTACAGCAACCAGTG	ATTGTTACAGACCTGCTGGAGAGA	chr9:40586051-40586207
208	chr3	129140163	129141010	TGGGCCTCTGTGAGAGAGATTATT	CCTCTCTTCCAAGTCCTACCCTTA	chr3:129140194-129140319
211	chr4	17771850	17772063	ATCAGCCTGAGTCAGCTTTCCT	CAGAGTTTCAAGTCCCAAAGACTG	chr4:17771851-17771951
299	chr15	95586502	95586869	AAAGGTCATGGAATACAGCACAGA	AAGAGAAGAGAGGTAACACGCCA	chr15:95586578-95586718
168	chr1	70762466	70763143	GCACTAAAGCTATCCATTTGGCTC	TGGAATTTCTAGATGAAATGGGTG	chr1:70762881-70763000
166	chr1	63272038	63272142	TGGCTTCTATCTTGATTCTGAGGA	AGCTCGCACAACCTCCATAAGT	chr1:63272038-63272142

Column label	Description
Clone_number	serial number of eTIP clones
Genomic_position	chromosome number for the clone location
	sequence start of the clone
	sequence end of the clone
Primer_sequence_forward	nucleotide sequence of forward primer
Primer_sequence_reverse	nucleotide sequence of reverse primer
Amplification_target	genomic position of the target

Table S3. RT-qPCR primers

		Forward_primer			Reverse_primer		
Gene_expression_group	Target	Length	%GC	Primer_sequence	Length	%GC	Primer_sequence
A1	*Camk2d*	21	52.0	GTCAACAGTGCCACGTCTTCA	21	52.0	GCATGGAACATCCGTCCAGTA
A1	*Itpr1*	20	50.0	CAAATGATGATGCTGCTGCC	21	57.0	CGTGTGAGCCTCTAACATGGC
A1	*Stx1a*	20	55.0	TCCTTTCTGGACCCCAACCT	20	60.0	TGCTCCTAGCACACCAGCAG
A1	*Pkia*	20	55.0	GACGATGTGGCGAATCTCCT	20	55.0	CAGGCCAGGTGATTTCAACC
A1	*Gabrb2*	24	54.2	ACTGGAAAGCTCAATGGCATGGGC	24	54.2	TGTCCCAACTGCGTGCCAACCTTA
A1	*Rrad*	20	45.0	AGCGACTGCAGCTGGAACTC	22	50.0	GGTCCGTCTTCTATACCACCAA
A2	*Gabra6*	20	50.0	TCCCCTGATGCCTTAGTCAA	21	42.9	TCGGGAAATGTCATCAAAAGC
A2	*Dnm1*	19	57.9	TGCCCCTCTGTGGTATTGC	19	52.6	TGGAGGTGGCACATTGGAG
A2	*Syp*	22	50.0	AGACATGGACGTGGTGAATCAG	21	57.0	CCACCACCTCTCAGAGTTCGA
A2	*Gabra1*	20	50.0	CACACCCCATCAATAGGTTC	20	55.0	GACAGAGGCAGTAAGGCAGA
A2	*Grm4*	20	55.0	CAGGACCAACGGACACTTGA	20	55.0	GCTGACTGTGAGGTGCCAAA
B2	*Matr3*	21	52.0	TTGCTGCTGCTACCCAGTCTT	21	57.0	CTGGCCTGGTCTGTATCTCCA
B2	*Ncl*	20	55.0	CAAAACCCACGGAGAGTCCA	20	55.0	GTGTGGGAACTGCAGCCTTT
B2	*Cat*	21	47.6	TGCCAAGGGAAAAGCTAACCT	21	42.9	TCGGGAAATGTCATCAAAAGC
B2	*Actb*	19	57.9	AACACCCCAGCCATGTACG	20	50.0	ATGTCACGCACGATTTCCCT
B2	*Top2b*	20	45.0	AGTAGAAACGGCTTGCAAAG	20	45.0	CTACATAGCTGCGAAATCCA
N	*Ndst3*	20	65.0	CGTCAGACCGAGCGTACTCC	19	63.0	CATCCAGGGACCAGGCATC
N	*Stxbp5l*	21	52.0	CTCGGCATACGGAATAGTTGC	20	55.0	AGAGGTCAATGGTCCCCATG
N	*Slc39a12*	21	48.0	TGAGCTACCCAAAGGCAATGT	21	48.0	CAAAATTGTGCAGGCCATCTC
N	*Ndst4*	20	55.0	TGTCTCGGGAAGAGCAAAGG	20	55.0	TTCGAGAGCTCCACGTTGTG
N	*Kctd8*	21	52.0	GTGGCAATCTGAACTGCTCCA	21	52.0	ATCAGTTAGGCGGTGACATGG

Table S4. Antibodies used for Western blotting

Target		Source	Host	Type	1st_Ab_conc
Camk2d	calcium/calmodulin-dependent protein kinase type IIδ	TransGenic Inc.	rabbit	Poly	1/3,000x
Itpr1	inositol 1,4,5-trisphosphate receptor type 1	Millipore	rabbit	Poly	0.15 μg/ml
Stx1a	syntaxin 1A	*1	mouse	Mono (clone mAb 6D2)	0.1 μg/ml
Gabra6	$GABA_A$ receptor, α6 subunit	Millipore	rabbit	Poly	1 μg/ml
Dnm1	dynamin 1	BD Transduction Lab.	mouse	Mono (clone 41)	0.125 μg/ml
Syp	synaptophysin	Roche Applied Science	mouse	Mono (clone SY38)	3 μg/ml
Matr3	matrin 3	*2	rabbit	Poly	1/50,000x
Ncl	nucleolin (C23)	*3	rabbit	Poly	0.2 μg/ml
Cat	catalase	Rockland Immunochemicals	rabbit	Poly	1 μg/ml
Top2b	DNA topoisomerase IIβ	*4	mouse	Mono (clone 3B6)	0.3 μg/ml

*1. kind gift of Dr. Masami Takahashi (Yoshida, A., Oho, C., Omori, A., Kuwahara, R., Ito, T. & Takahashi, M. HPC-1 is associated with synaptotagmin and omega-conotoxin receptor. J Biol Chem 267, 24925-8 (1992).)

*2. kind gift of Dr. Ryozo Kuwano (Niigata University, Japan)

*3. Tsutsui, K., Tsutsui, K., Hosoya, O., Sano, K. & Tokunaga, A. Immunohistochemical analyses of DNA topoisomerase II isoforms in developing rat cerebellum. J Comp Neurol 431, 228-39 (2001).

*4. Tsutsui, K., Tsutsui, K., Sano, K., Kikuchi, A. & Tokunaga, A. Involvement of DNA topoisomerase IIβ in neuronal differentiation. J Biol Chem 276, 5769-78 (2001).
