# Supplementary figures and images for: Topoisomerase IIβ Activates a Subset of Neuronal Genes that Are Repressed in AT-Rich Genomic Environment
Source: PLoS One. 2008 Dec 31;3(12):e4103. doi: 10.1371/journal.pone.0004103 (PMC2605559; doi:10.1371/journal.pone.0004103)

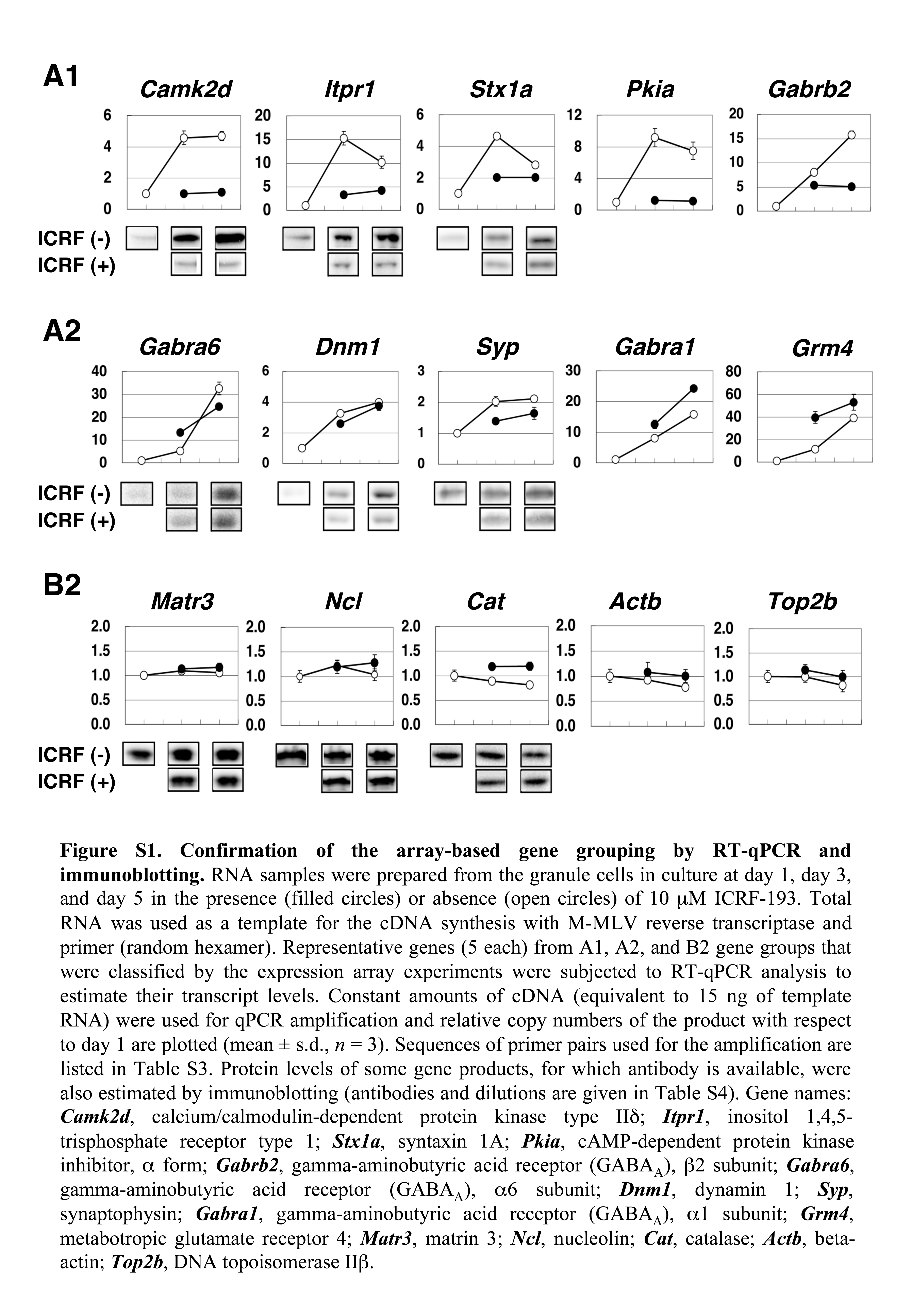

Supplement: Figure S1 — Confirmation of the array-based gene grouping by RT-qPCR and immunoblotting (2.58 MB TIF) [file pone.0004103.s003.tif]

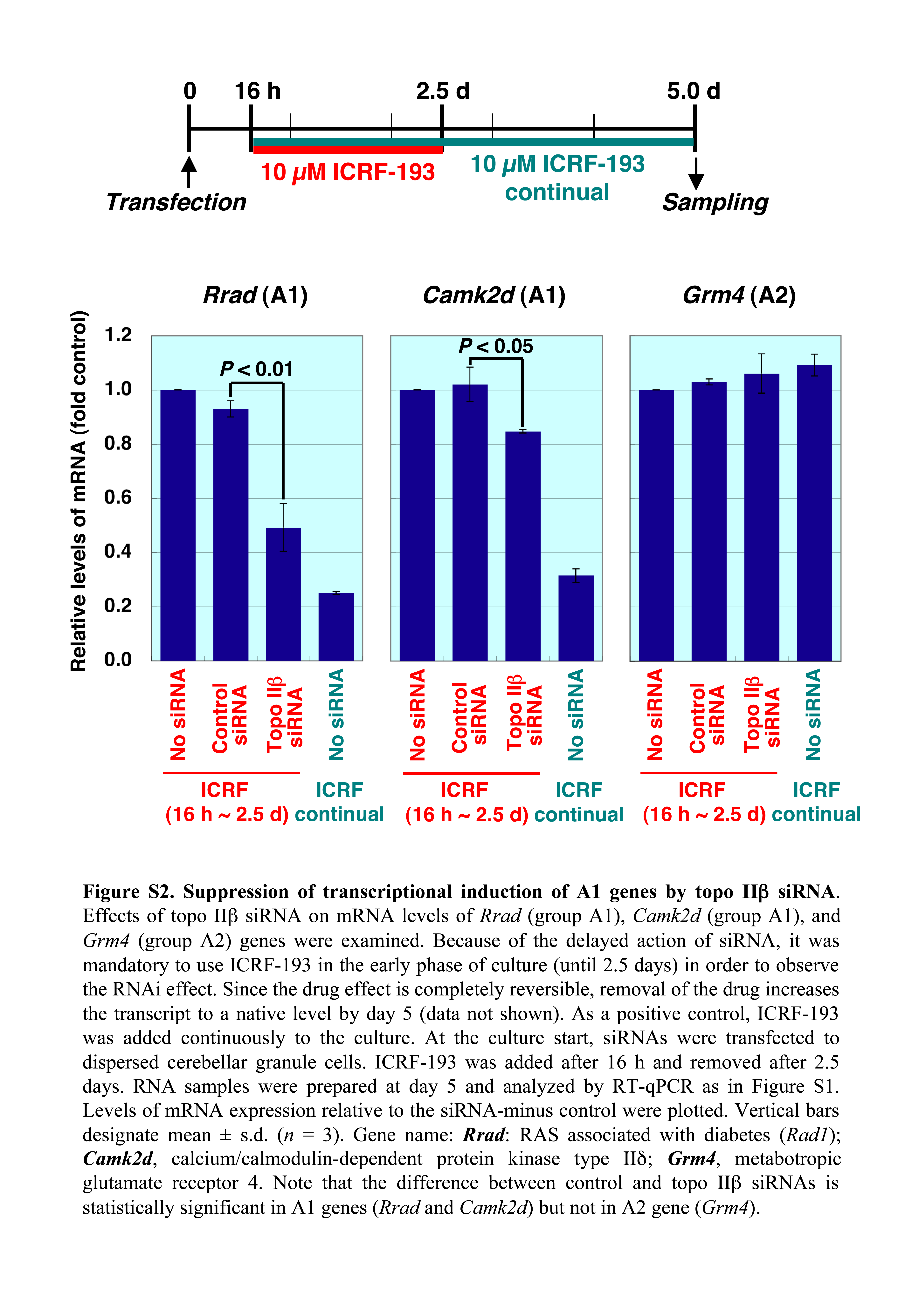

Supplement: Figure S2 — Suppression of transcriptional induction of A1 genes by topo ΙΙβ siRNA (1.51 MB TIF) [file pone.0004103.s004.tif]

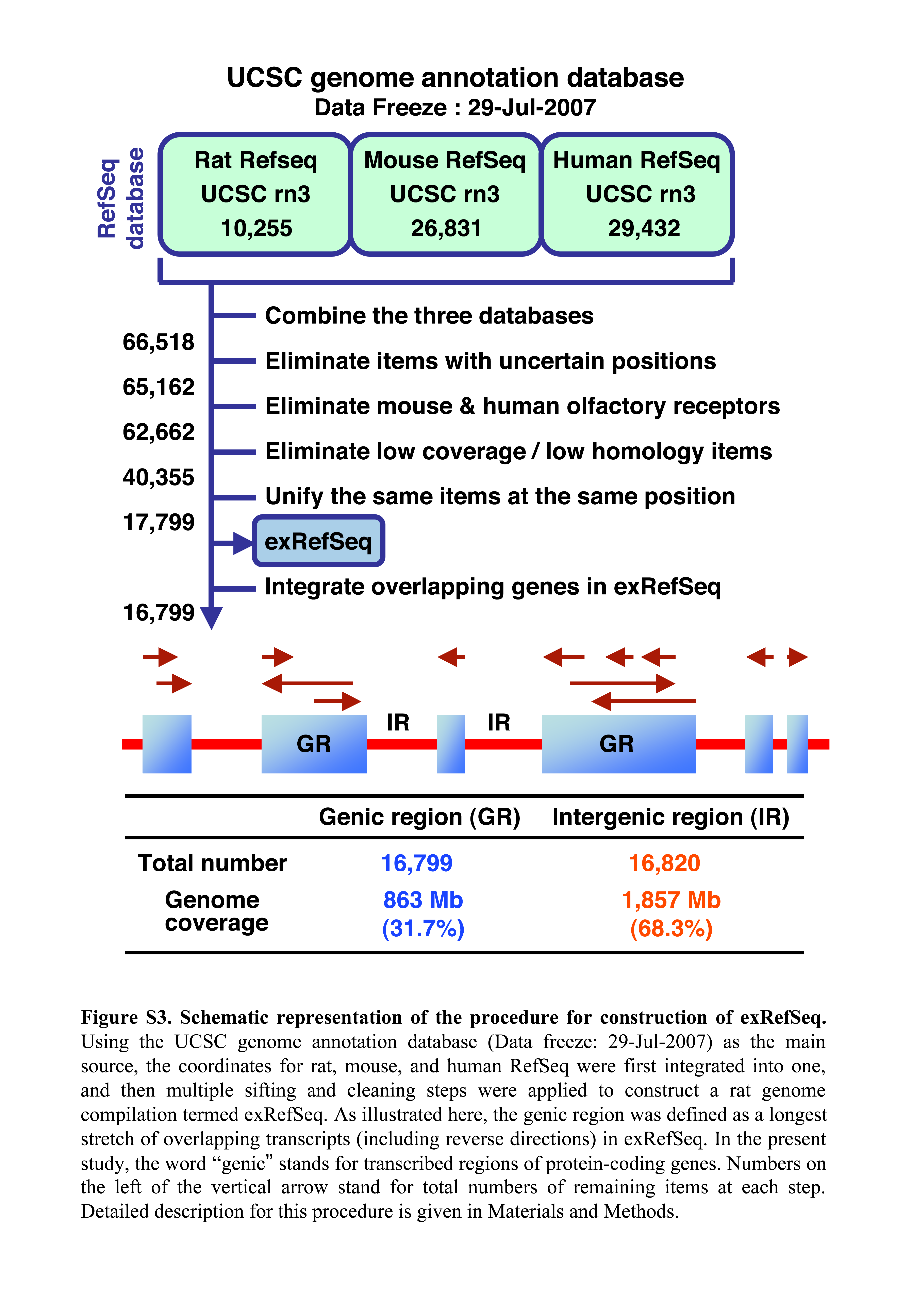

Supplement: Figure S3 — Schematic representation of the procedure for construction of exRefSeq (1.36 MB TIF) [file pone.0004103.s005.tif]

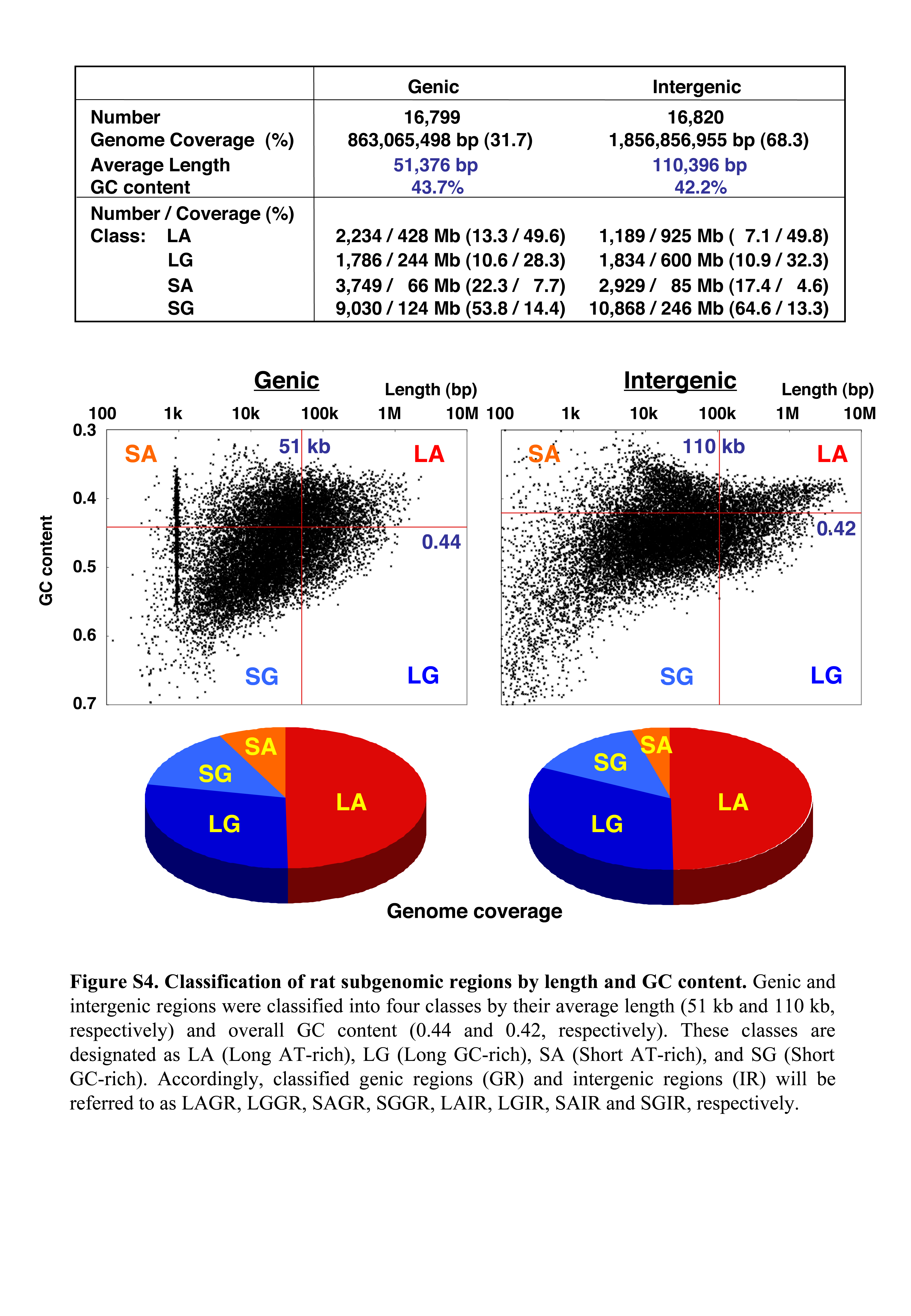

Supplement: Figure S4 — Classification of rat subgenomic regions by length and GC content (3.02 MB TIF) [file pone.0004103.s006.tif]

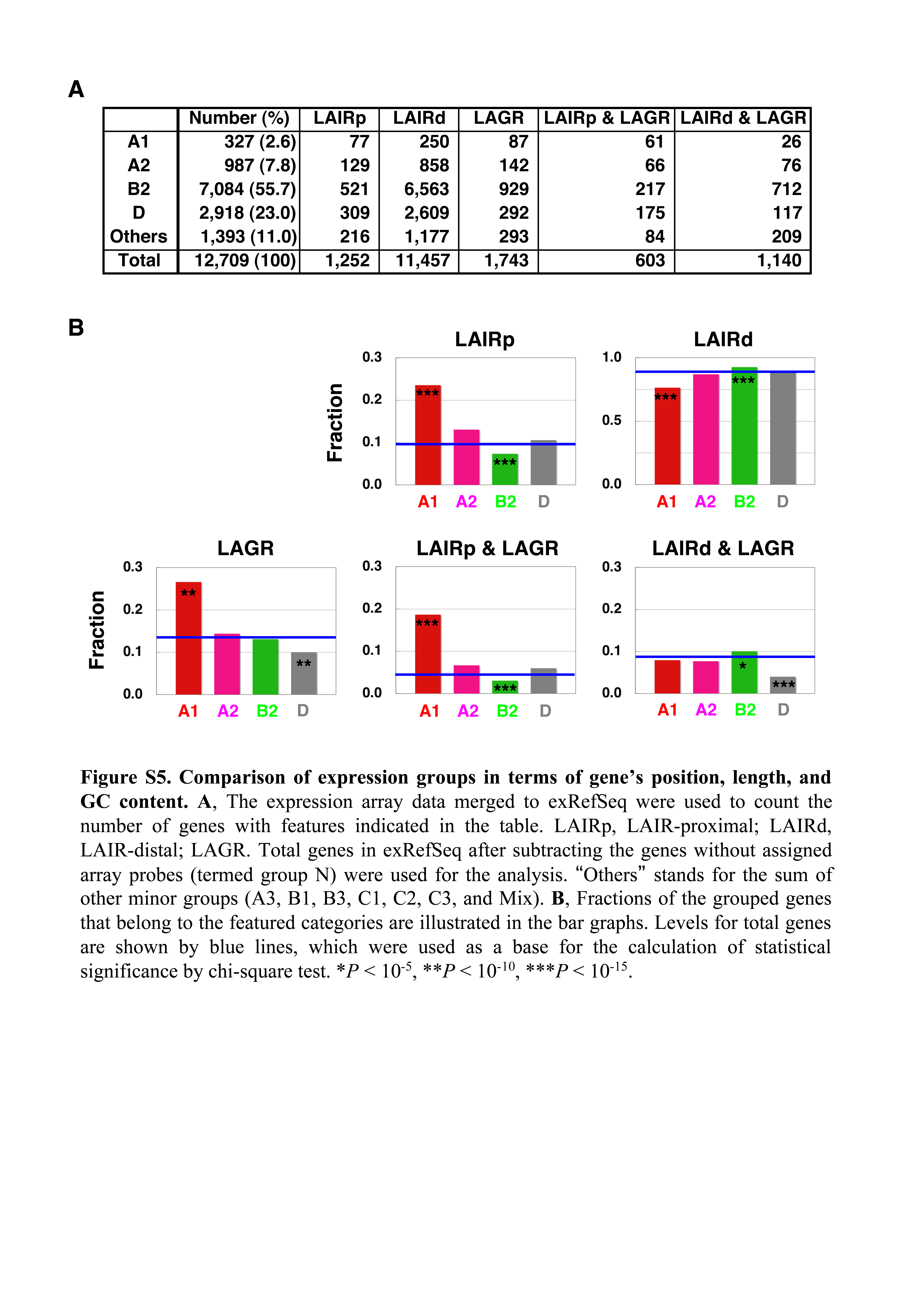

Supplement: Figure S5 — Comparison of expression groups in terms of gene's position, length, and GC content (1.12 MB TIF) [file pone.0004103.s007.tif]

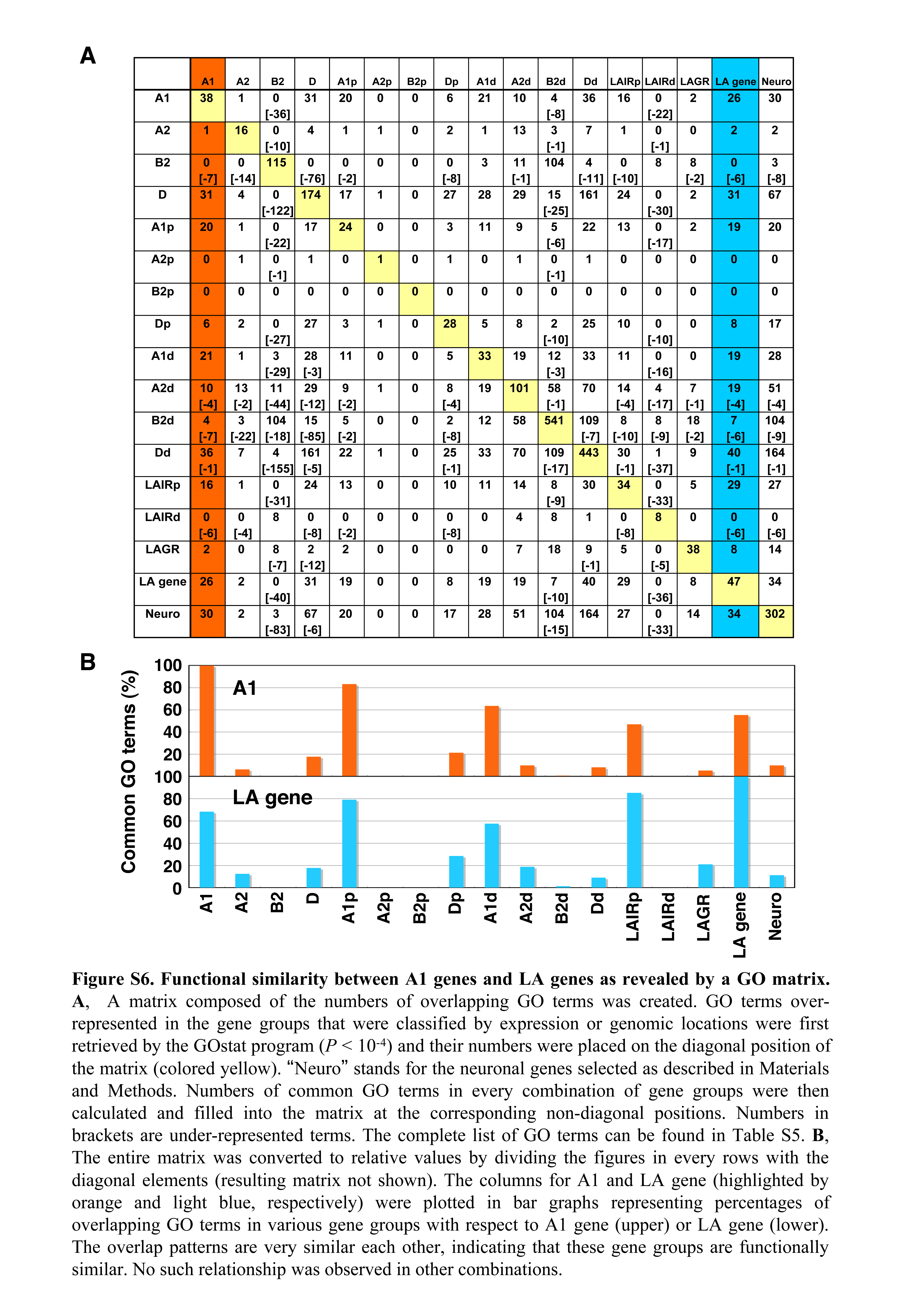

Supplement: Figure S6 — Functional similarity between A1 genes and LA genes as revealed by a GO matrix (1.70 MB TIF) [file pone.0004103.s008.tif]

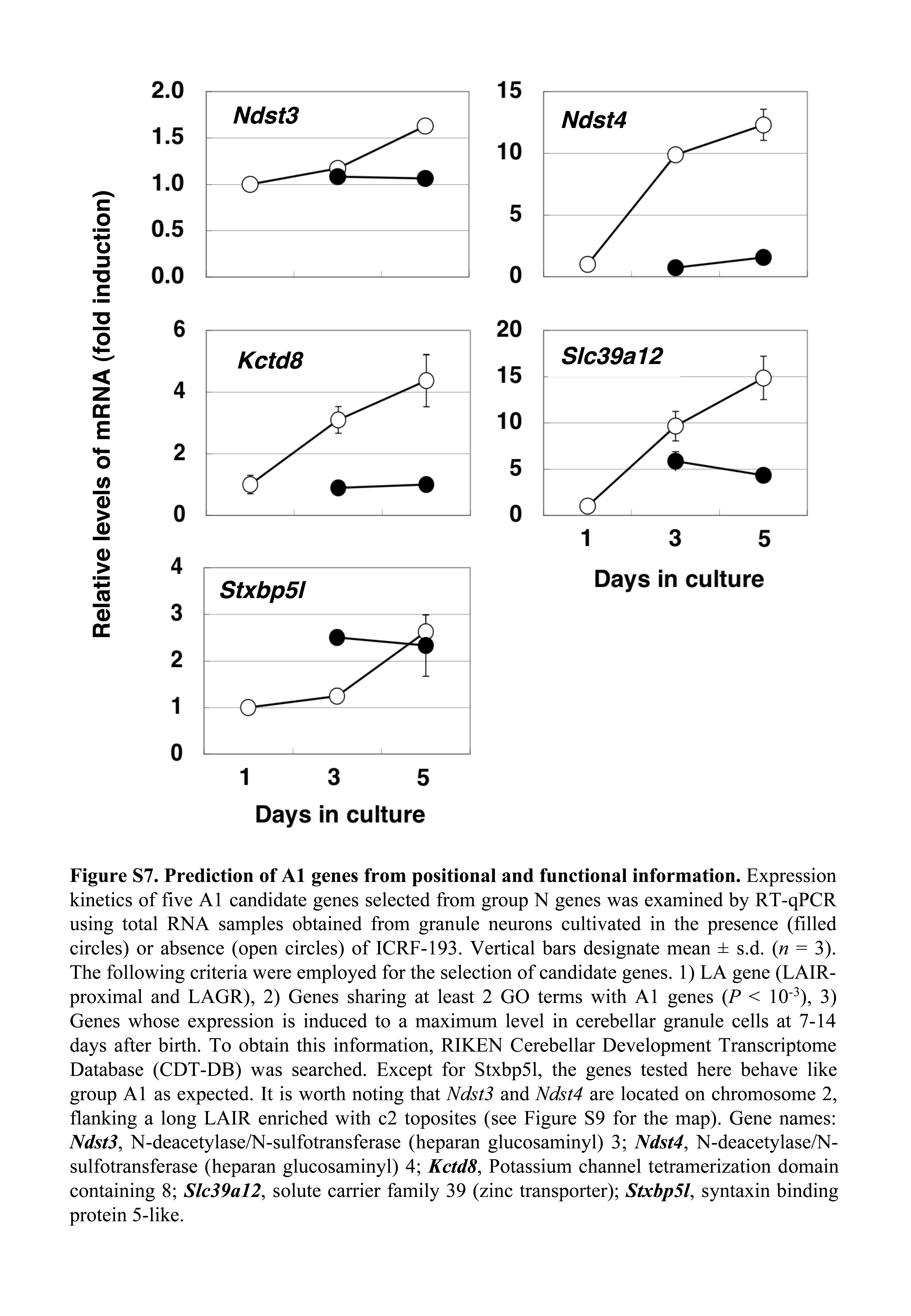

Supplement: Figure S7 — Prediction of A1 genes from positional and functional information (1.65 MB TIF) [file pone.0004103.s009.tif]

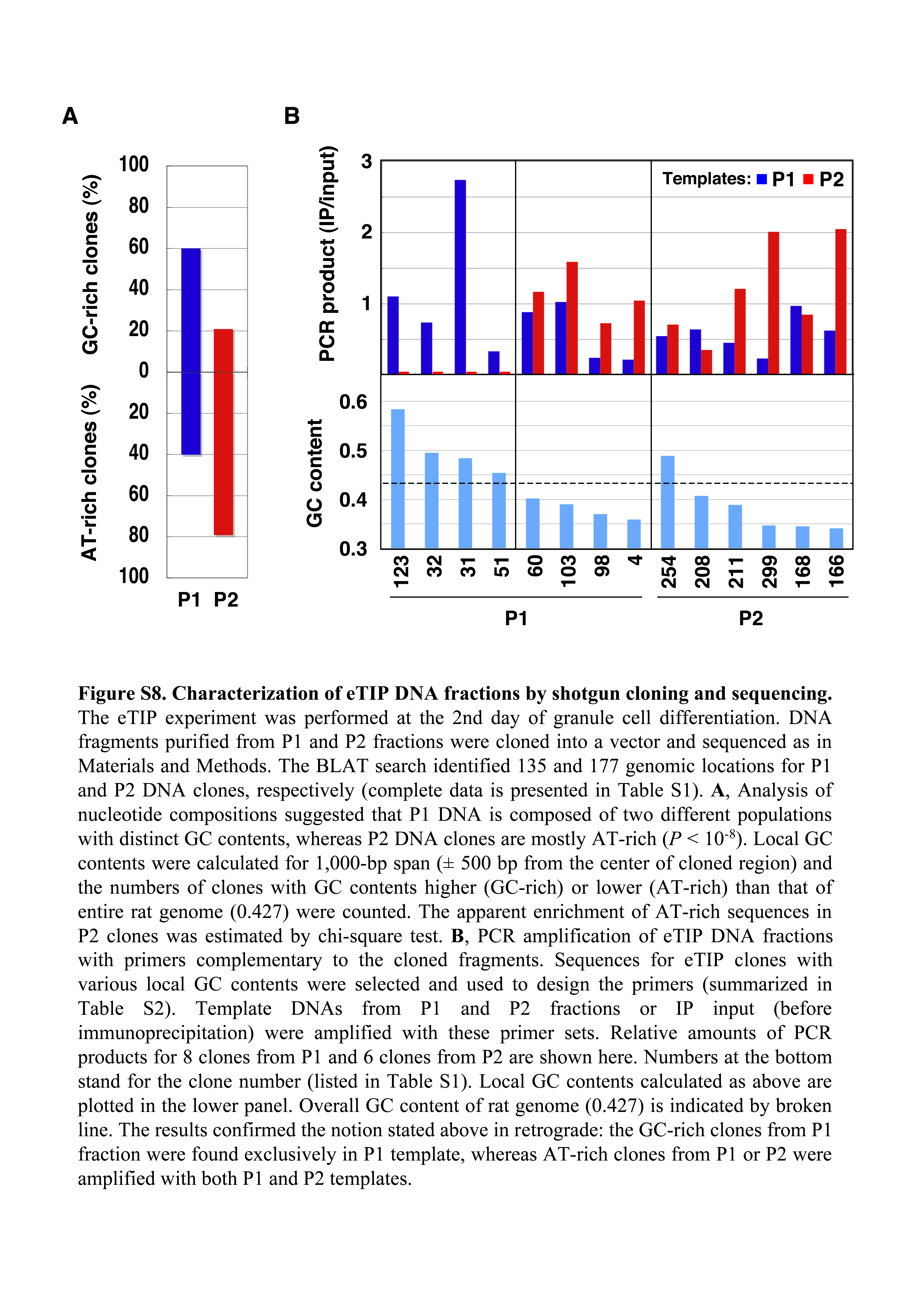

Supplement: Figure S8 — Characterization of eTIP DNA fractions by shotgun cloning and sequencing (1.57 MB TIF) [file pone.0004103.s010.tif]

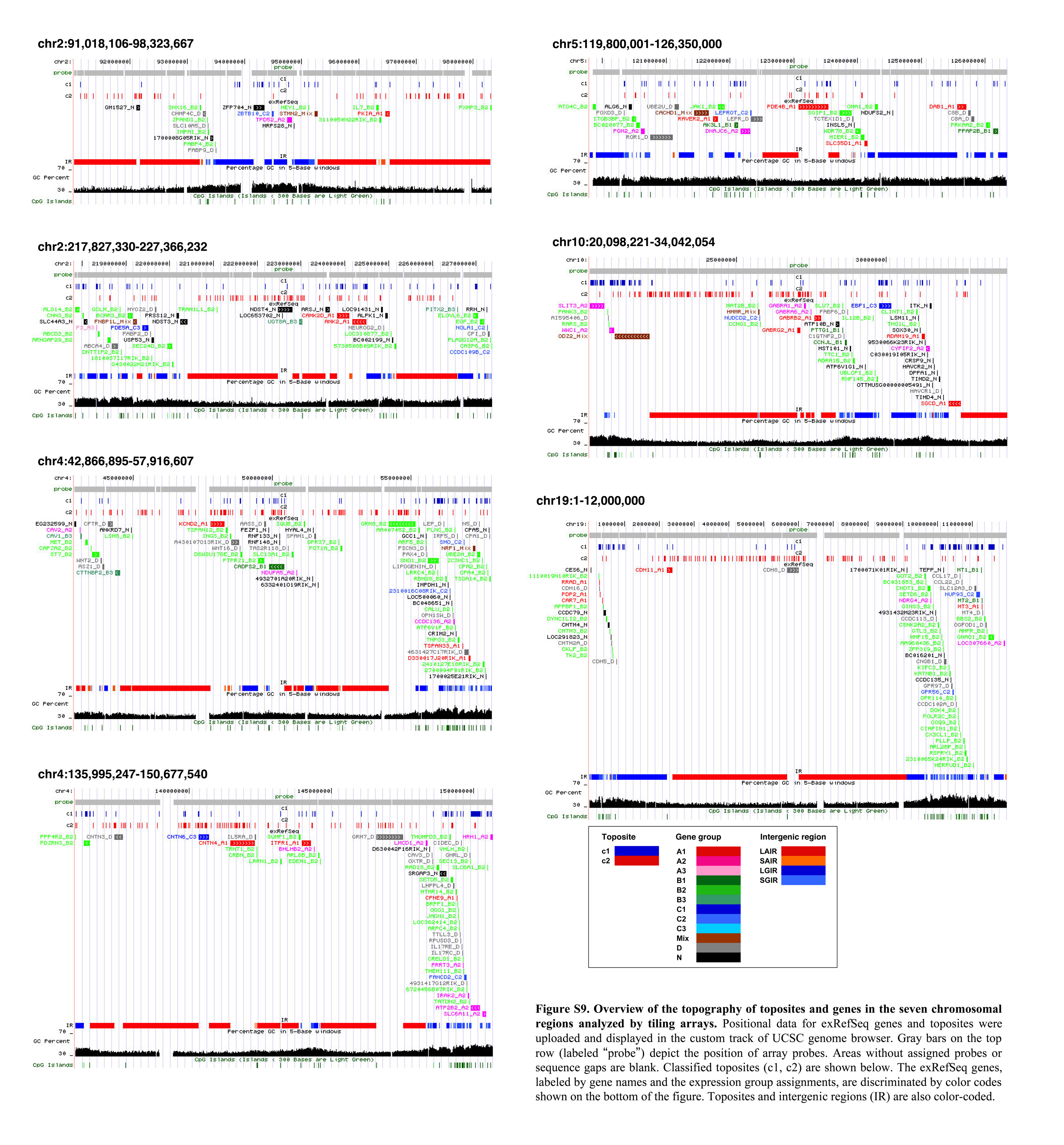

Supplement: Figure S9 — Overview of the topography of toposites and genes in the seven chromosomal regions analyzed by tiling arrays (7.31 MB TIF) [file pone.0004103.s011.tif]

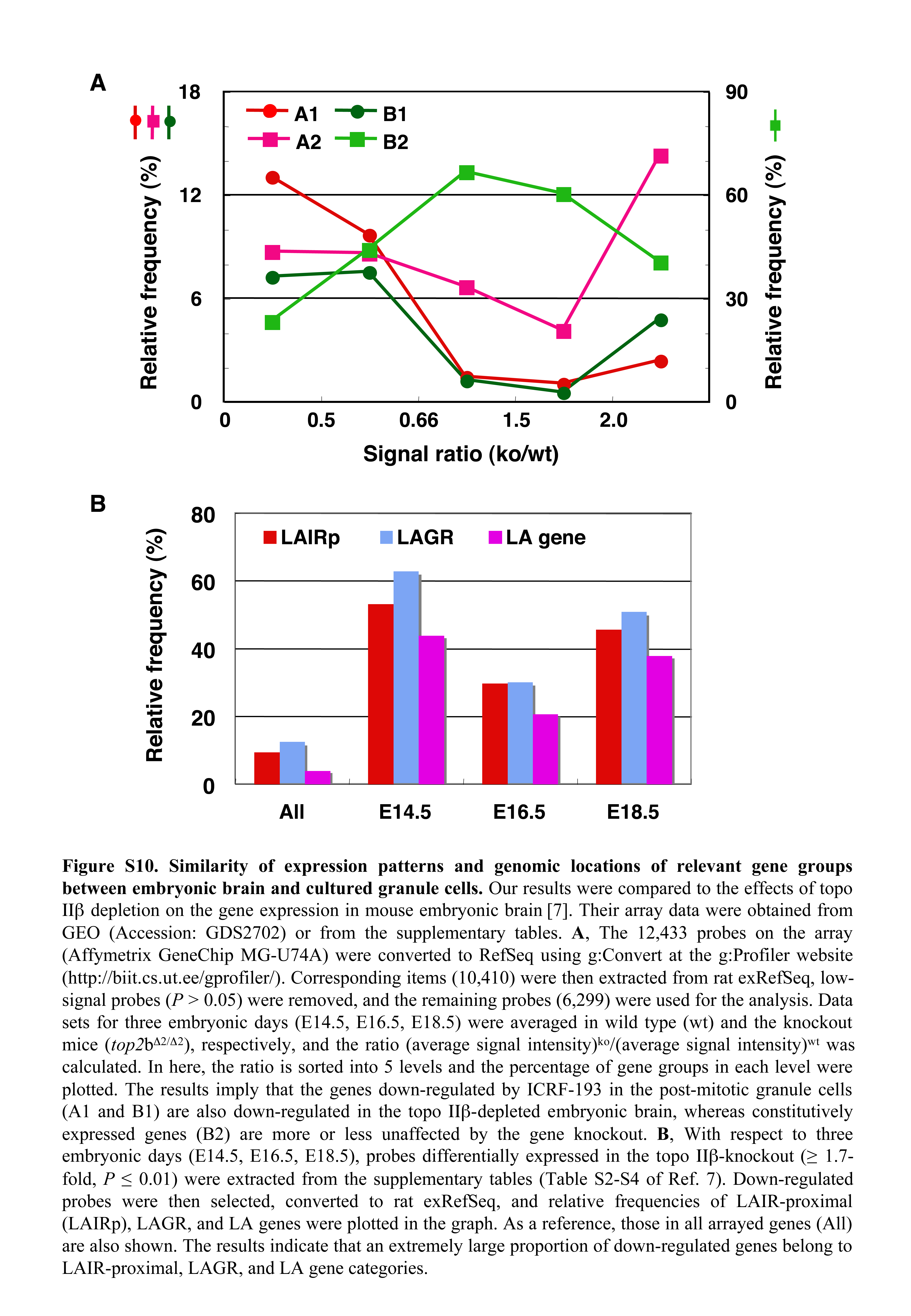

Supplement: Figure S10 — Similarity of expression patterns and genomic locations of relevant gene groups between embryonic brain and cultured granule cells (1.45 MB TIF) [file pone.0004103.s012.tif]

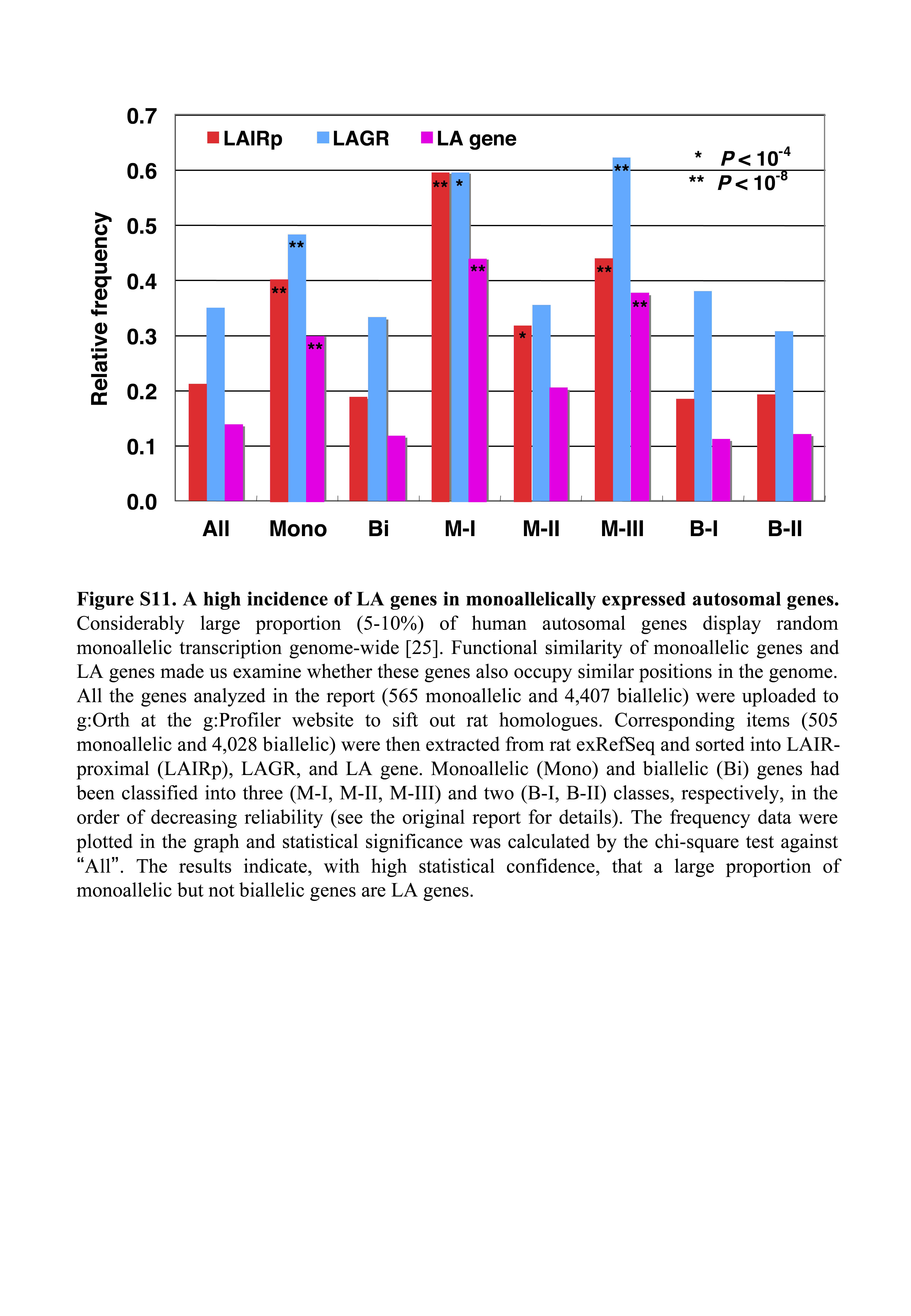

Supplement: Figure S11 — A high incidence of LA genes in monoallelically expressed autosomal genes (1.10 MB TIF) [file pone.0004103.s013.tif]
